# A cascade double 1,4-addition/intramolecular annulation strategy for expeditious assembly of unsymmetrical dibenzofurans

**DOI:** 10.1038/s42004-021-00478-2

**Published:** 2021-03-25

**Authors:** Xinwei He, Ruxue Li, Pui Ying Choy, Mengqing Xie, Jiahui Duan, Qiang Tang, Yongjia Shang, Fuk Yee Kwong

**Affiliations:** 1 Key Laboratory of Functional Molecular Solids, Ministry of Education, College of Chemistry and Materials Science, Anhui Normal University, Wuhu, 241000 PR China; 2 State Key Laboratory of Synthetic Chemistry and Department of Chemistry, The Chinese University of Hong Kong, New Territories, Shatin, Hong Kong SAR PR China

**Keywords:** Sustainability, Synthetic chemistry methodology

## Abstract

Existing synthetic routes for accessing dibenzofuran core have intrinsic regioselectivity, limiting the substitution patterns available in heteropolycyclic arene products. Here we report a double 1,4-conjugate addition/intramolecular annulation cascade reaction between propargylamines and two equivalents of imidazolium methylides that allows efficient access of structurally versatile dibenzofurans. This transition metal-free protocol proceeds smoothly under bench-top air atmosphere and offers easy manipulation of substituents on the dibenzofuran core, and also provides good-to-excellent product yields with good functional group tolerance, particularly the –Br and –Cl groups which are often incompatible with existing metal-catalyzed C–C and/or C–O bond ring-forming processes. It is worth noting that ladder-type π-systems with all-arene quarternary carbon structure can be straightforwardly generated upon simple late-stage functionalization.

## Abstract (web summary)

Existing routes to dibenzofurans have intrinsic regioselectivity, limiting the substitution patterns available in the products. Here a double 1,4-conjugate addition-annulation cascade between propargylamines and imidazolium methylides provides direct access to dibenzofurans with a complementary substitution pattern.

## Introduction

**Fig. 1:**
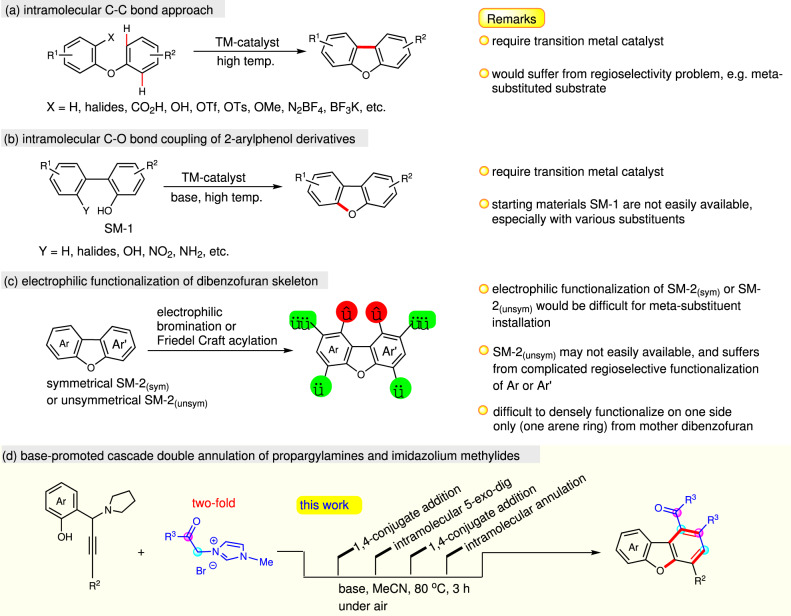
Modern approaches for accessing dibenzofuran skeletons. A brief glance of modern synthetic methods for preparing multiple arene-substituted dibenzofurans. **a** Recent examples of intramolecular cyclization of non-substituted or *ortho*-substituted aryloxybenzenes in constructing dibenzofuran framework under transition metals catalyst systems. **b** Intramolecular *O*-arylation of *ortho*-arylphenols for making dibenzofuran skeleton. **c** Direct electrophilic functionalization of dibenzofuran molecules. **d** A cascade reaction between propargylic amine and imidazolium methylide to access dibenzofuran skeletons (this work).

Dibenzofuran-containing heterocycle constitutes an important structural motif in many natural products, pharmaceutically intermediates, and functional materials^1–7^. Indeed, these flanked-arenes furans are shown to have particularly appealing characteristics, such as antibiotics, antimalarial, antiallergy, anti-inflammatory, and anticancer activities^8–11^. They also express their versatility in photoelectronic materials, especially in phosphorescent organic light-emitting diodes (PhOLEDs)^12–14^, and chemical probes in biological process investigations^15–17^. Given the significance of these unique benzofuran scaffolds, efforts have been made extensively toward the exploration of a new transition metal-catalyzed coupling approach allowing modern access to dibenzofuran with fascinating structural complexity. Representative synthetic strategies in this context are intramolecular ring closure pathways for achieving targeted dibenzofurans, either from a starting material of diaryl ether or *ortho*-arylphenol, via an aromatic carbon–carbon or carbon–oxygen bond-forming process, respectively (Fig. 1). Recent examples of intramolecular cyclization of non-substituted (X = H) or *ortho*-substituted aryloxybenzenes (X = halides, CO_2_H, OH, OTf, BF_3_K and etc.) were shown to be successful in constructing dibenzofuran framework under palladium^18–35^, silver^36^, rhodium^37^, or gold^38^ catalyst systems (Fig. 1a). In addition to C−C bond formation protocol, intramolecular *O*-arylation of *ortho*-arylphenols also exhibited as an alternative synthetic scheme for making dibenzofuran skeleton (Fig. 1b)^39–49^.

Nevertheless, employing expensive transition metal catalysts and associated ligands, and particularly limited direct availability of substrate **SM-1** with various substituents, would hamper their generality for synthesizing unsymmetrical dibenzofuran with wanted substitution patterns. Despite the advancement of transition metal-catalyzed strategy, direct functionalization of dibenzofuran molecule is also adoptable (Fig. 1c)^50^. However, the availability of pattern-desirable starting material **SM-2** may not be straightforward, and in principle, the regioselectivity of electrophilic substitution is not favorable at the *meta*-position of arene to the oxygen atom in dibenzofurans. Recently, impressive synthetic method establishments were disclosed in which they do not require transition metal catalysts, for instance, the diazotization-comprised cyclization of *ortho*-(aryloxy)aniline promoted by visible light^51^ or NaNO_2_/TFA;^52,53^ the substrate-dependent arylation of phenols with bromonitroarenes;^54^ the reaction of 2-aryl-3-nitrochromenes with pyridinium ylides;^55^ and the three-component reaction between 2-hydroxy-*β*-nitrostyrenes, sulfur ylides, and alkynes for the synthesis of dibenzofuran acrylates^56,57^.

*ortho*-Quinone methide (*o*-QM) is a versatile reagent for a variety of polycycle syntheses^58^. In particular, the alkyne-containing *o*-QM, namely alkynyl *ortho*-quinone methide (*o*-AQM) displays unique feature of possible *exo*- or *endo*-cyclization towards the alkynyl moiety^59–66^. In continuing our recent interest in investigating complex polycyclic structure assembly^67,68^ and *o*-AQM chemistry^69–71^, we are intrigued whether the acyl carbene motif can be doubly incorporated to *o*-AQM in achieving the densely-substituted arene on one side of dibenzofuran framework.

Herein, we report a cascade reaction between propargylic amines and imidazolium methylides (Fig. 1d). This base-promoted protocol allows modular assembly of dibenzofurans with a complementary substitution pattern, and exhibits good functional group tolerance, particularly tolerating -Br and -Cl groups which offer potential for late-stage product modification via cross-coupling technology. It is worth noting that this method is operationally simple, as the reaction can be conveniently performed under bench-top air atmosphere, and is complementary to frequently Br-group-incompatible transition metal-catalyzed annulative C_(Ar)_−C_(Ar’)_ and C_(Ar)_−O bond construction reactions.

## Results and discussion

### Reaction optimization

Propargylic amine **1a** and imidazolium salt **2a** were chosen as the prototypical substrates for our initial reaction condition investigations (Table 1). Putting KO*t*-Bu in the reaction system allowed the formation of desired product **3aa** (entries 1–3). Upon comparing imidazolium salt **2a** to other similar reaction partners, e.g., **2-quin**, **2-*iso*-quin**, **2-*p*-NMe**_**2**_**py,** and **2-py**, imidazolium salt **2a** gave the best result (entry 2 vs entries 4–7). To our delight, mixing the commonly used bases provided better outcomes, particularly the KO*t*-Bu/K_2_CO_3_ and KO*t*-Bu/KOH combinations (entries 8–13). Further investigation of the base stoichiometry offered better product yields (entries 14–16). Having the fruitful base combination, we next surveyed the regularly used solvents (entries 17–23). MeCN and DMF were found to be the best solvents of choice (entries 15 and 17). We intended to use MeCN for further screening owing to its operational simplicity. Room temperature conditions did not allow the reaction to proceed (entry 24). Further variation of reaction temperatures revealed that 80 °C is the optimal condition (entries 25–26). Extension of reaction time did not find beneficial to the product yield (entry 28). Thus, the optimal reaction conditions were found to be KO*t*-Bu/KOH in MeCN at 80 °C for 3 h. It is worthy to note that this cascade reaction proceed-well under bench-top air atmosphere.

**Table 1:** Optimization of reaction conditions^a^. Propargylic amine **1a** and imidazolium salt **2a** were used as the prototypical substrates for initial reaction condition investigations.


entry	base (equiv.)	solvent	T./°C	yield/%^b^
1	KO*t*-Bu (0.5)	MeCN	80	15
2	KO*t*-Bu (1.0)	MeCN	80	32
3	KO*t*-Bu (1.5)	MeCN	80	17
4^c^	KO*t*-Bu (1.0)	MeCN	80	18
5^d^	KO*t*-Bu (1.0)	MeCN	80	15
6^e^	KO*t*-Bu (1.0)	MeCN	80	23
7^f^	KO*t*-Bu (1.0)	MeCN	80	26
8	KO*t*-Bu (1.0)/NaOH (1.0)	MeCN	80	35
9	KO*t*-Bu (1.0)/NaHCO_3_ (1.0)	MeCN	80	32
10	KO*t*-Bu (1.0)/NaOEt (1.0)	MeCN	80	23
11	KO*t*-Bu (1.0)/K_2_CO_3_ (1.0)	MeCN	80	43
12	KO*t*-Bu (1.0)/KOH (1.0)	MeCN	80	45
13	KO*t*-Bu (1.0)/K_2_S_2_O_8_ (1.0)	MeCN	80	20
14	KO*t*-Bu (1.0)/K_2_CO_3_ (2.0)	MeCN	80	68
15	KO*t*-Bu (1.0)/KOH (2.0)	MeCN	80	83
16	KO*t*-Bu (1.0)/KOH (3.0)	MeCN	80	74
17	KO*t*-Bu (1.0)/KOH (2.0)	DMF	80	82
18	KO*t*-Bu (1.0)/KOH (2.0)	DMSO	80	75
19	KO*t*-Bu (1.0)/KOH (2.0)	THF	80	19
20	KO*t*-Bu (1.0)/KOH (2.0)	EtOH	80	9
21	KO*t*-Bu (1.0)/KOH (2.0)	toluene	80	20
22	KO*t*-Bu (1.0)/KOH (2.0)	DCE	80	n.d.^*i*^
23	KO*t*-Bu (1.0)/KOH (2.0)	DCM	80	23
24	KO*t*-Bu (1.0)/KOH (2.0)	MeCN	rt	n.r.^*j*^
25	KO*t*-Bu (1.0)/KOH (2.0)	MeCN	45	58
26	KO*t*-Bu (1.0)/KOH (2.0)	MeCN	90	67
27^g^	KO*t*-Bu (1.0)/KOH (2.0)	MeCN	80	68
28^h^	KO*t*-Bu (1.0)/KOH (2.0)	MeCN	80	82

^a^Reaction conditions: **1a** (0.2 mmol), **2a** (0.4 mmol), base (equivalences were with respect to **1a**) were stirred in solvent for 3 h under bench-top air atmosphere. ^b^Isolated yields were reported. ^c^**2-quin** was used instead of **2a**. ^d^**2-iso-quin** was used instead of **2a**. ^e^**2-*p*-NMe**_**2**_**py** was used instead **2a**. ^f^**2-py** was used instead of **2a**. ^g^Reaction time was 2 h. ^h^Reaction time was 5 h. ^i^Not determined. ^j^No reaction.

### Substrate scope

**Fig. 2:**
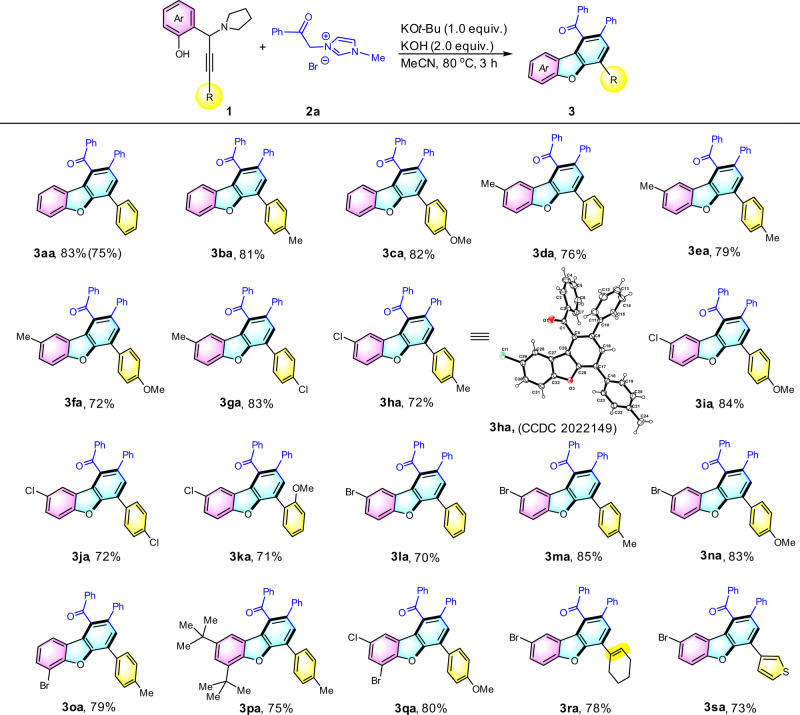
Substrate scope of propargylamines. Reaction conditions: **1** (0.2 mmol), **2a** (0.4 mmol), KO*t*-Bu (0.2 mmol), KOH (0.4 mmol) were stirred in acetonitrile (2 mL) at 80 °C for 3 h under bench-top air atmosphere. Isolated yields were reported. Isolated yield of 10-fold scale in parenthesis.

With the optimized reaction conditions in hand, we next investigated the scope of propargylic amine with various substituents (Fig. 2). In general, the desired dibenzofurans were delivered in good-to-excellent yields. No significant electronic effect of the alkynyl arene moiety at the propargylic scaffold was found (products **3da**, **3ea**, **3fa**, and **3ga**). Similarly, the electronically-varied substituents (e.g., –Me and –Cl) at the *para*-position of the phenolic moiety did not affect the product yields (products **3ea** vs **3ha**). Structure of **3ha** was unambiguously characterized by single-crystal X-ray crystallography (see Supplementary Fig. 1 and Table 1, Supplementary Data 1 for CIF file). The –Br substituent at *para*- and *ortho*-position of the phenolic arene unit remained intact during the course of the reaction (products **3la**, **3ma**, **3na**, and **3oa**). This –Br group compatibility is of high attractiveness as this group can be further straightforwardly functionalized using established cross-coupling technology. In addition to halo-substituents, the steric bulky *tert*-butyl group at the *ortho*-phenolic-position was found to be tolerable towards the ring-forming process (product **3pa**). Cyclohexenyl and thienyl units remained untouched in this cascade annulation and furnished the expected products **3ra** and **3sa** in 78% and 73% yields, respectively.

**Fig. 3:**
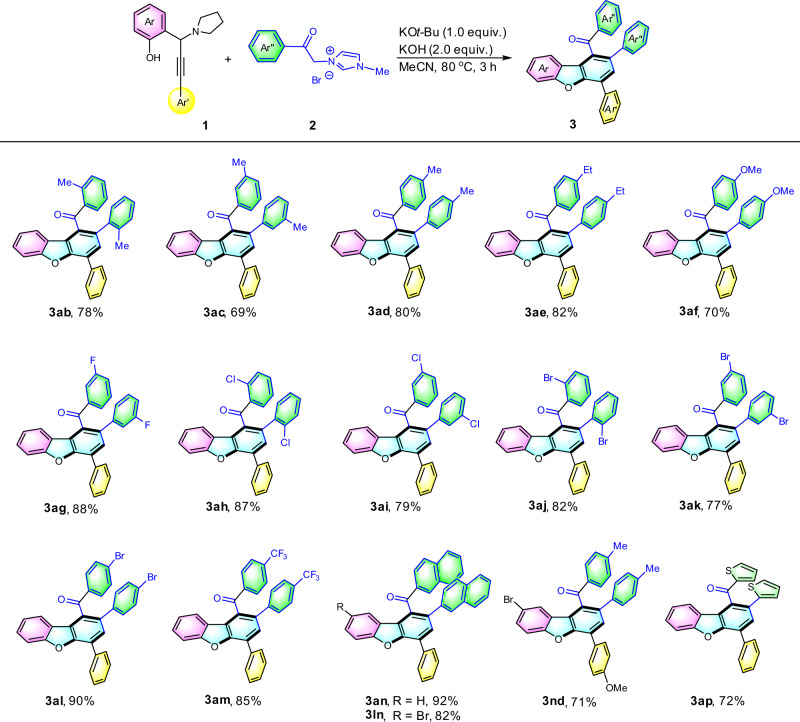
Substrate scope of propargylamines and 1-methyl-1*H*-imidazolium methylides. Reaction conditions: **1** (0.2 mmol), **2** (0.4 mmol), KO*t*-Bu (0.2 mmol), KOH (0.4 mmol) were stirred in acetonitrile (2 mL) at 80 °C for 3 h under bench-top air atmosphere. Isolated yields were reported.

The scope of the reaction was further tested using various benzoyl imidazolium methylide derivatives (Fig. 3). Essentially no steric influence of the arene at **2** was found and the desired products were afforded in good yields (products **3ab**, **3ac**, **3ad**, and **3ae**). Substrates bearing halo groups, e.g., fluoro, chloro, and bromo were successfully employed in this reaction, yielding the resulting products **3ag**-**3al** in 77% to 90% yields. Highly electron-withdrawing substituent –CF_3_ was compatible, affording the corresponding products **3am**. With appropriate imidazolium methylide, the π-extended structure was able to be constructed in 92% yield (product **3an**).

### Mechanistic studies

**Fig. 4:**
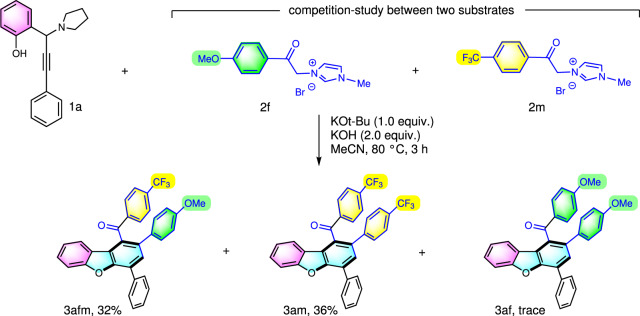
A competition experiment. A competition-study between two electronically different imidazolium methylides **2f** and **2m** were performed.

A competition-study between two electronically different imidazolium methylides **2f** and **2m** were performed (Fig. 4). Two main products **3afm** and **3am** were isolated in 32% and 36% yields, respectively. This experiment clearly reflected that the electronically poor methylide **2m** underwent deprotonation faster and thus subsequent 1,4-conjugate addition to *o*-AQM. The product of **3af** was not detected essentially that further added to this mechanistic proposal.

**Fig. 5:**
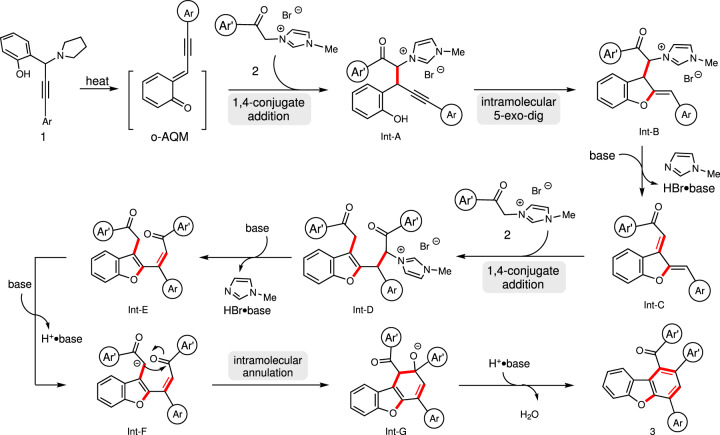
A proposed mechanism. A hypothetical reaction mechanism for the formation of multiple arene-substituted dibenzofurans is presented.

A plausible reaction mechanism is proposed in Fig. 5. The alkynyl *o*-quinone methide (*o*-AQM) intermediate is generated upon releasing of piperidine via intramolecular 1,5-*H* shift under basic conditions^58^. Subsequent 1,4-conjugate addition of imidazolium methylide **2** furnishes adduct **Int-A**. Intramolecular 5-*exo-dig* annulation of **Int-A** gives intermediate **Int-B**, which subsequently forms **Int-C** via a facile *β*-H elimination. The second 1,4-conjugate addition of another molecule of **2** delivers **Int-D**, and then converts to **Int-E** under an elimination process. The **Int-F** is formed upon deprotonation and generates **Int-G** via a second annulation step. Rearomatization of **Int-G** finally furnishes product **3**.

### Synthetic transformations and applications

**Fig. 6:**
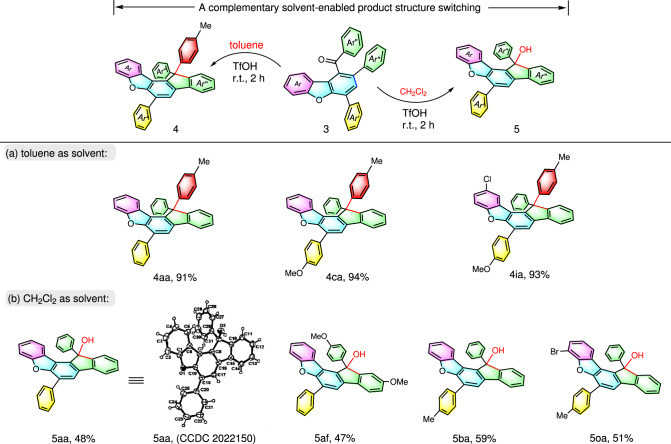
Possible late-stage elaborations dibenzofuran 3. Reaction conditions: dibenzofurans **3** (0.2 mmol), TfOH (0.6 mmol) were stirred in toluene (2 mL, synthesis of compound **4** for 1 h) or in DCM (2 mL, synthesis of compound **5** for 2 h) at room temperature. Isolated yields were reported.

Pentacene has been successful in advancing organic material applications^72^. The high charge carrier mobility of acenes is believed to be originated from the highly ordered π-π-interaction stacking between adjacent molecules^73^. Nevertheless, the stability of this pentacene towards practical application still has room for improvement. Recently, the ladder-type heteroacenes was reported to have both high charge mobility and stability under ambient conditions^74^. With regard to this impressive finding, we are interested to devise an investigation for assembling fluorene-containing spiro-ladder-type heteroacenes^75^. The all-arene quaternary carbon unit would be useful for easy management of distinctive spatial arrangement of the π-system for desirable mobility-tuning^76,77^. To our delight, the initial attempt of using superacid-promoted synthetic strategy^78,79^ was successful in delivering a spiro-ring-fused system (Fig. 6). In the presence of TfOH in toluene at room temperature, a series of spiro-ladder-type molecules **4aa**, **4ca**, and **4ia** were able to be obtained in 91%, 94%, and 93% yields, respectively (Fig. 6). It is interesting to show that toluene served as both reagent and solvent for this transformation. Conversely, upon alternating the solvent to dichloromethane, the serendipitous products **5aa**, **5af**, **5ba**, and **5oa** were formed (the tertiary alcohol product **5aa** was unambiguously confirmed by single-crystal X-ray crystallography, see Supplementary Fig. 2 and Table 2, Supplementary Data 2 for CIF file). These products indeed offer high opportunity for further functionalization via a simple –OH group transformation^80,81^, and thus allows rich entities for new material investigations.

In conclusion, we have succeeded in showing a doubly 1,4-conjugate addition/annulation cascade process for facile access of structurally versatile dibenzofurans. This transition metal-free protocol proceeds smoothly under bench-top air atmosphere and realizes easy manipulation of substituents on one of the flanked-arenes of dibenzofuran core. The present modular method exhibits good product framework diversity and complexity, decent product yields, and allows good functional group tolerance, particularly the –Br and –Cl groups where they are often found impermissible in existing Pd-catalyzed aromatic C–C or C–O bond ring-forming processes. It is worthy to note that materially attractive spiro-ladder-type π-system with all-arene quaternary carbon feature can also be simply attained via a one-step subsequent functionalization.

## Methods

### General procedures for the synthesis of propargylamines 1

To a 25 mL round-bottom flask equipped with a magnetic stir bar were added pyrrolidine (1.2 mmol), aldehyde (1.0 mmol), acetylene (1.2 mmol), copper (I) iodide (10 mol%), and toluene (3 mL). The mixture was degassed and backfilled with nitrogen, and then stirred in an oil bath preheated to 100 °C for 5 h (monitored by TLC). After the reaction completed (as determined using TLC), the reaction mixture was cooled to room temperature, diluted with CH_2_Cl_2_ (10 mL), and filtered through a thin pad of silica gel. The filter cake was washed with CH_2_Cl_2_, and the combined filtrate was concentrated in a vacuum. The crude product was purified by flash column chromatography on silica gel to afford the corresponding propargylamines.

### General procedure for the synthesis of imidazolium methylides 2

1-Methyl-1*H*-imidazole (410 mg, 5.0 mmol) was added to 2-bromoethanones derivatives (5.0 mmol) in dry acetonitrile (5 mL). After stirring for 2 to 5 h at room temperature, the precipitate formed was filtered off and washed with acetonitrile to afford the desired imidazolium methylides **2**, which can be used to next reaction without further purification. Other ylides used were synthesized according to these procedures.

### General procedures for the synthesis of dibenzofurans 3

A mixture of propargylamines **1** (0.2 mmol), imidazolium methylides **2** (0.4 mmol), potassium *t*-butoxide (0.2 mmol), and potassium hydroxide (0.4 mmol) were added to a resealable screw-capped Schlenk tube under air atmosphere. Acetonitrile (2 mL) was then added. The tube sealed with a Teflon-coated cap and the resulting mixture was stirred in an oil bath preheated to 80 °C for 3 h (monitored by TLC). Upon completion of the reaction, the reaction mixture was cooled to room temperature, and the solvent was removed under reduced pressure. The residue was purified using flash column chromatography with a silica gel (200–300 mesh), using ethyl acetate and petroleum ether (1:20, v/v) as the elution solvent to give desired products **3**. For NMR spectra, see Supplementary Figures 3–72).

### General procedure for the competition experiment (synthesis of 3afm, and 3am)

A mixture of 2-(3-phenyl-1-(pyrrolidin-1-yl)prop-2-yn-1-yl)phenol (**1a**) (0.2 mmol, 0.56 g), 3-(2-(4-methoxyphenyl)-2-oxoethyl)-1-methyl-1*H*-imidazol-3-ium bromide (**2** **f**) (0.4 mmol), 1-methyl-3-(2-oxo-2-(4-(trifluoromethyl)phenyl)ethyl)-1*H*-imidazol-3-ium bromide (**2m**) (0.4 mmol), potassium *t*-butoxide (0.2 mmol, 0.22 g), and potassium hydroxide (0.4 mmol, 0.22 g) were added to a resealable screw-capped Schlenk tube. Acetonitrile (3 mL) was then added. The tube sealed with a Teflon-coated cap and the resulting mixture was stirred in an oil bath preheated to 80 °C for 3 h (monitored by TLC). Upon completion of the reaction, the reaction mixture was cooled to room temperature, and the solvent was removed under reduced pressure. The residue was purified using flash column chromatography with a silica gel (200-300 mesh), using ethyl acetate and petroleum ether (1:20, v/v) as the elution solvent to give desired product **3fm** and **3am** in 32% and 36% yield, respectively. For NMR spectra, see Supplementary Figures 73, 74).

### 10-Fold scale synthesis of compound 3aa

A mixture of 2-(3-phenyl-1-(pyrrolidin-1-yl)prop-2-yn-1-yl)phenol (**1a**) (2.0 mmol, 0.56 g), 1-methyl-3-(2-oxo-2-phenylethyl)-1*H*-imidazol-3-ium bromide (**2a**) (4.0 mmol, 1.12 g), potassium *t*-butoxide (2.0 mmol, 0.22 g), and potassium hydroxide (4.0 mmol, 0.22 g) were added to a resealable screw-capped Schlenk tube. Acetonitrile (10 mL) was then added. The tube sealed with a Teflon-coated cap and the resulting mixture was stirred in an oil bath preheated to 80 °C for 3 h (monitored by TLC). Upon completion of the reaction, the reaction mixture was cooled to room temperature, and the solvent was removed under reduced pressure. The residue was purified using flash column chromatography with a silica gel (200–300 mesh), using ethyl acetate and petroleum ether (1:20, v/v) as the elution solvent to give desired product **3aa** in 75% yield.

### General procedures for the synthesis of compound 4

Dibenzofuran **3** (0.2 mmol) was mixed with TfOH (0.6 mmol) in a round bottom flask. Toluene (2 mL) was then added. The resulting mixture was stirred at room temperature (25 °C) for 1 h (monitored by TLC). Upon completion of the reaction, the solvent was removed under reduced pressure. The residue was purified using flash column chromatography with a silica gel (200–300 mesh), using ethyl acetate and petroleum ether as the elution solvent to give desired product **4**. For NMR spectra, see Supplementary Figures 75–80).

### General procedures for the synthesis of compound 5

Dibenzofuran **3** (0.2 mmol) was mixed with TfOH (0.6 mmol) in a round bottom flask. Dichloromethane (2 mL) was then added. The resulting mixture was stirred at room temperature (25 °C) for 2 h (monitored by TLC). Upon completion of the reaction, the solvent was removed under reduced pressure. The residue was purified using flash column chromatography with a silica gel (200–300 mesh), using ethyl acetate and petroleum ether as the elution solvent to give desired product **5**. For NMR spectra, see Supplementary Figures 81–88).

## Supplementary information

Supplementary Information

Description of Additional Supplementary Files

Supplementary Data 1

Supplementary Data 2

## Data Availability

The data sets generated and analyzed during the current study are included in the Supplementary Information File and also available from the corresponding authors on request. For full methods, see Supplementary Methods. For ^1^H NMR, and ^13^C NMR spectra see Supplementary Figs. 3–88 and the GC-MS spectra for mechanistic investigation see Supplementary Figs. 89–95. For X-ray crystallographic figures, see Supplementary Fig. 1 for compound **3****ha** and Fig. 2 for compound **5aa**. The X-ray crystallographic coordinates for structures reported in this Article have been deposited at the Cambridge Crystallographic Data Centre (CCDC), under deposition numbers CCDC 2022149 (**3****ha –** Supplementary Data 1) and 2022150 (**5aa –** Supplementary Data 2). These data can be obtained free of charge from The Cambridge Crystallographic Data Centre via www.ccdc.cam.ac.uk/data_request/cif. The coordinates for the corresponding structures are available in Supplementary Tables 1 and 2.
