# Supplementary material for: A cascade double 1,4-addition/intramolecular annulation strategy for expeditious assembly of unsymmetrical dibenzofurans
Source: Commun Chem. 2021 Mar 25;4:42. doi: 10.1038/s42004-021-00478-2 (PMC9814151; doi:10.1038/s42004-021-00478-2)
Supplement: Supplementary file 1 — Supplementary Information [file 42004_2021_478_MOESM1_ESM.pdf]

Electronic Supplementary Information

A cascade double 1,4-addition/intramolecular annulation strategy for expeditious assembly of unsymmetrical dibenzofurans

Xinwei He[1,2,*], Ruxue Li[1], Pui Ying Choy[2], Mengqing Xie[1], Jiahui Duan[1], Qiang Tang[1], Yongjia Shang[1,*], and Fuk Yee Kwong[2,*]

[1]Key Laboratory of Functional Molecular Solids, Ministry of Education, College of Chemistry and Materials Science, Anhui Normal University, Wuhu 241000, P.R. China

[2]State Key Laboratory of Synthetic Chemistry and Department of Chemistry, The Chinese University of Hong Kong, New Territories, Shatin, Hong Kong SAR, P.R. China

Email: xinweihe@mail.ahnu.edu.cn, shyj@mail.ahnu.edu.cn, fykwong@cuhk.edu.hk

Table of contents

1. **General consideration** 2
2. **General procedures for the synthesis of propargylamines 1** 2
3. **General procedure for the synthesis of imidazolium methylides 2** 3
4. **General procedure for the synthesis of dibenzofurans 3** 3
5. **10-Fold scale synthesis of compound 3aa** 4
6. **General procedures for the synthesis of compound 3afm** 4
7. **General procedures for the synthesis of compound 4** 5
8. **General procedures for the synthesis of compound 5** 5
9. **Characterization data for all compounds** 6
10. **X-ray crystallographic data of compound 3ha** 28
11. **X-ray crystallographic data of compound 5aa** 30
12. **^{1}H and ^{13}C NMR spectra for all compounds** 32
13. **GC-MS spectra for mechanistic investigations** 75
14. **Supplementary References** 79

Supplementary Methods

1. General consideration

Unless otherwise specified, all reagents and starting materials were purchased from commercial sources and used as received without purification. The solvents were purified and dried using standard procedures.[1] The chromatography solvents were technical grade and distilled prior to use. Flash chromatography was performed using 200-300 mesh silica gel with the indicated solvent system according to standard techniques. The ^{1}H and ^{13}C NMR spectra were recorded on a 400 MHz or 500 MHz and 100 MHz or 125 MHz NMR spectrometers, unless otherwise specified. Chemical shifts (δ) in parts per million were reported relative to the residual signals of chloroform (7.26 ppm for ^{1}H and 77.0 ppm for ^{13}C), and all ^{13}C NMR were recorded with proton broadband decoupling and indicated as $^{13}C\{^{1}H\}$ NMR. Multiplicities are described as s (singlet), d (doublet), t (triplet), q (quartet), or m (multiplet), and the coupling constants (*J*) are reported in Hertz (Hz). HRMS analysis with a quadrupole time-of-flight mass spectrometer yielded ion mass/charge (m/z) ratios in atomic mass units. IR spectra were measured as dry films (KBr), and the peaks are reported in terms of wave number (cm^{-1}). The melting points were measured using SGWX-4 melting point apparatus.

2. General procedures for the synthesis of propargylamines 1

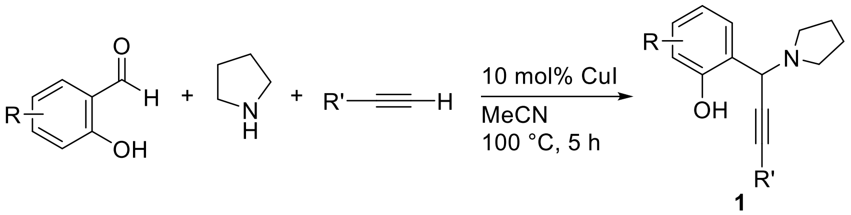

1

To a 25 mL round-bottom flask equipped with a magnetic stir bar were added pyrrolidine (1.2 mmol), aldehyde (1.0 mmol), acetylene (1.2 mmol), copper (I) iodide (10 mol%) and toluene (3 mL). The mixture was degassed and backfilled with nitrogen, and then stirred in an oil bath preheated to 100 °C for 5 h (monitored by TLC). After the reaction completed (as determined using TLC), the reaction mixture was cooled to

room temperature, diluted with CH_2Cl_2 (10 mL) and filtered through a thin pad of silica gel. The filter cake was washed with CH_2Cl_2, and the combined filtrate was concentrated in vacuum. The crude product was purified by flash column chromatography on silica gel to afford the corresponding propargylamines.[2]

3. General procedure for the synthesis of imidazolium methylides 2

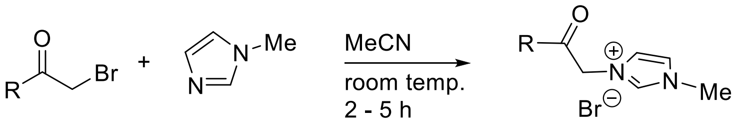

2

1-Methyl-1*H*-imidazole (410 mg, 5.0 mmol) was added to 2-bromoethanones derivatives (5.0 mmol) in dry acetonitrile (5 mL). After stirring for 2 to 5 h at room temperature, the precipitate formed was filtered off and washed with acetonitrile to afford the desired imidazolium methylides **2**, which can be used to next reaction without further purification. Other ylides used were synthesized according to these procedures.

4. General procedure for the synthesis of dibenzofurans 3

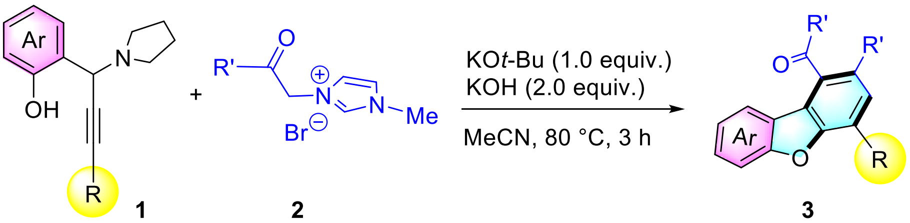

1 **2** **3**

A mixture of propargylamines **1** (0.2 mmol), imidazolium methylides **2** (0.4 mmol), potassium *t*-butoxide (0.2 mmol), and potassium hydroxide (0.4 mmol) were added to a resealable screw-capped Schlenk tube under air atmosphere. Acetonitrile (2 mL) was then added. The tube sealed with a Teflon-coated cap and the resulting mixture was stirred in an oil bath preheated to 80 °C for 3 h (monitored by TLC). Upon completion of the reaction, the reaction mixture was cooled to room temperature, and the solvent was removed under reduced pressure. The residue was purified using flash column chromatography with a silica gel (200-300 mesh), using ethyl acetate and

petroleum ether (1:20, v/v) as the elution solvent to give desired products **3**.

5. 10-Fold scale synthesis of compound 3aa

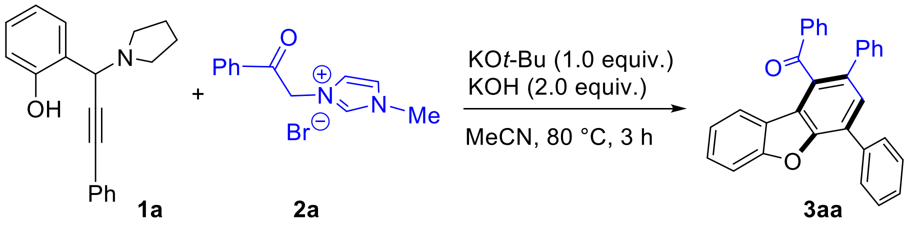

A mixture of 2-(3-phenyl-1-(pyrrolidin-1-yl)prop-2-yn-1-yl)phenol (**1a**) (2.0 mmol, 0.56 g), 1-methyl-3-(2-oxo-2-phenylethyl)-1*H*-imidazol-3-ium bromide (**2a**) (4.0 mmol, 1.12 g), potassium *t*-butoxide (2.0 mmol, 0.22 g), and potassium hydroxide (4.0 mmol, 0.22 g) were added to a resealable screw-capped Schlenk tube. Acetonitrile (10 mL) was then added. The tube sealed with a Teflon-coated cap and the resulting mixture was stirred in an oil bath preheated to 80 °C for 3 h (monitored by TLC). Upon completion of the reaction, the reaction mixture was cooled to room temperature, and the solvent was removed under reduced pressure. The residue was purified using flash column chromatography with a silica gel (200-300 mesh), using ethyl acetate and petroleum ether (1:20, v/v) as the elution solvent to give desired product **3aa** in 75% yield.

6. General procedures for the synthesis of compound 3afm

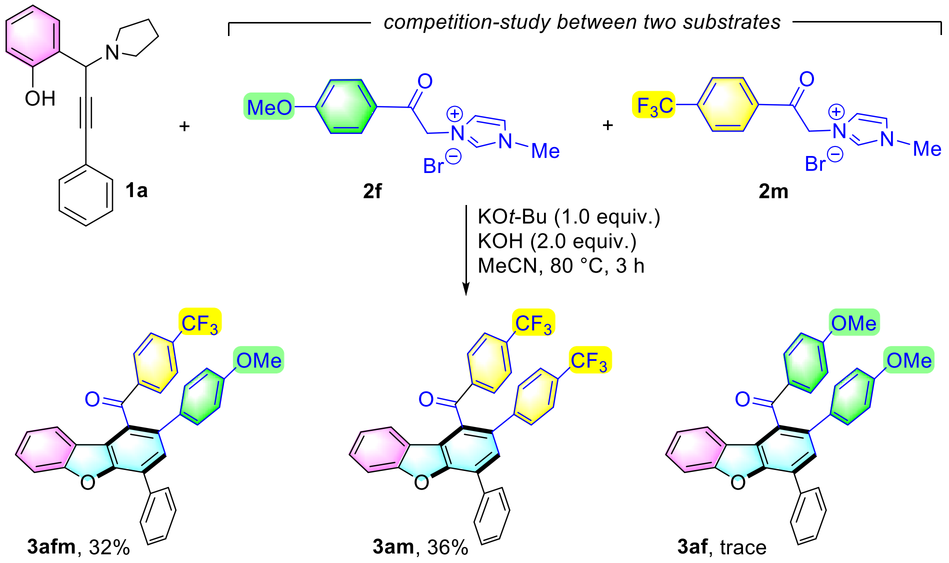

A mixture of 2-(3-phenyl-1-(pyrrolidin-1-yl)prop-2-yn-1-yl)phenol (**1a**) (0.2 mmol, 0.056 g), 3-(2-(4-methoxyphenyl)-2-oxoethyl)-1-methyl-1*H*-imidazol-3-ium bromide (**2f**) (0.4 mmol, 0.12 g), 1-methyl-3-(2-oxo-2-(4-(trifluoromethyl)phenyl)ethyl)-1*H*-imidazol-3-ium bromide (**2m**) (0.4 mmol, 0.14 g), potassium *t*-butoxide (0.2 mmol, 0.22 g), and potassium hydroxide (0.4 mmol, 0.022 g) were added to a resealable screw-capped Schlenk tube. Acetonitrile (3 mL) was then added. The tube sealed with a Teflon-coated cap and the resulting mixture was stirred in an oil bath preheated to 80 °C for 3 h (monitored by TLC). Upon completion of the reaction, the reaction mixture was cooled to room temperature, and the solvent was removed under reduced pressure. The residue was purified using flash column chromatography with a silica gel (200-300 mesh), using ethyl acetate and petroleum ether (1:20, v/v) as the elution solvent to give desired product **3fm** and **3am** in 32% and 36% yield, respectively.

7. General procedures for the synthesis of compound 4

Dibenzofuran **3** (0.2 mmol) was mixed with TfOH (0.6 mmol) in a round bottom flask. Toluene (2 mL) was then added. The resulting mixture was stirred at room temperature (25) °C for 1 h (monitored by TLC). Upon completion of the reaction, the reaction mixture was cooled to room temperature, and the solvent was removed under reduced pressure. The residue was purified using flash column chromatography with a silica gel (200-300 mesh), using ethyl acetate and petroleum ether as the elution solvent to give desired product **4**.

8. General procedures for the synthesis of compound 5

Dibenzofuran **3** (0.2 mmol) was mixed with TfOH (0.6 mmol) in a round bottom flask. dichloromethane (2 mL) was then added. The resulting mixture was stirred at room temperature (25) °C for 2 h (monitored by TLC). Upon completion of the reaction, the reaction mixture was cooled to room temperature, and the solvent was removed under reduced pressure. The residue was purified using flash column chromatography with a silica gel (200-300 mesh), using ethyl acetate and petroleum ether as the elution

solvent to give desired product **5**.

9. Characterization data for all compounds

(2,4-Diphenyldibenzo[*b,d*]furan-1-yl)(phenyl)methanone (Figure 2, compound 3aa)

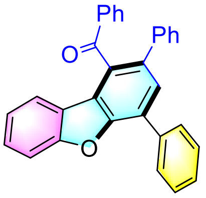

mp: 218–219 °C; TLC (ethyl acetate:petroleum ether, 1:20 v/v): R_f = 0.6; white solid; ^{1}H NMR (400 MHz, $CDCl_3$): δ 8.09–7.98 (m, 2H), 7.84–7.77 (m, 2H), 7.74 (s, 1H), 7.69–7.57 (m, 4H), 7.54–7.40 (m, 5H), 7.37–7.26 (m, 3H), 7.26–7.16 (m, 3H); ^{13}C{^{1}H} NMR (100 MHz, $CDCl_3$): δ 197.96, 156.91, 152.68, 139.90, 137.18, 135.77, 133.51, 131.53, 129.77, 129.59, 129.03, 128.87, 128.74, 128.47, 128.39, 128.32, 127.84, 127.38, 126.91, 123.08, 122.81, 122.73, 122.66, 111.87; HRMS (m/z): $[M+H]^+$ calcd. for $C_{31}H_{21}O_2$, 425.1536; found, 425.1531.

Phenyl(2-phenyl-4-(*p*-tolyl)dibenzo[*b,d*]furan-1-yl)methanone (Figure 2, compound 3ba)

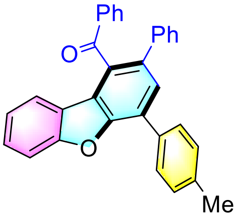

mp: 188–189 °C; TLC (ethyl acetate:petroleum ether, 1:20 v/v) R_f = 0.5; white solid; ^{1}H NMR (400 MHz, $CDCl_3$): δ 7.90 (d, J = 8.0 Hz, 2H), 7.77–7.74 (m, 2H), 7.69 (s, 1H), 7.62–7.58 (m, 2H), 7.46–7.43 (m, 1H), 7.42–7.37 (m, 5H), 7.29–7.27 (m, 1H), 7.25–7.22 (m, 2H), 7.22–7.11 (m, 3H), 2.48 (s, 3H); ^{13}C{^{1}H} NMR (100 MHz, $CDCl_3$): δ 198.04, 156.87, 152.66, 139.96, 138.34, 137.18, 135.73, 133.47, 132.81, 131.18, 129.76, 129.59, 129.56, 128.87, 128.51, 128.43, 128.29, 127.76, 127.32, 126.91, 123.01, 122.74, 122.72, 122.63, 111.85, 21.39; HRMS (m/z): $[M+H]^+$ calcd. for $C_{32}H_{23}O_2$, 439.1693; found, 439.1696.

(4-(4-Methoxyphenyl)-2-phenyldibenzo[*b,d*]furan-1-yl)(phenyl)methanone (Figure 2, compound 3ca)

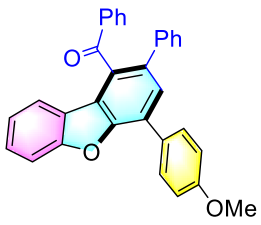

mp: 155–156 °C; TLC (ethyl acetate: petroleum ether, 1:20 v/v); R_f = 0.4 to afford a light yellow solid in 82% yield (73 mg). ^{1}H NMR (400 MHz, $CDCl_3$): δ 8.00–7.91 (m, 2H), 7.80–7.72 (m, 2H), 7.66 (s, 1H), 7.64–7.56 (m, 2H), 7.46–7.35 (m, 4H), 7.28 (s, 1H), 7.25–7.15 (m, 5H), 7.14–7.09 (m, 2H), 3.91 (s, 3H); ^{13}C{^{1}H} NMR (100 MHz, $CDCl_3$): δ 198.05, 159.80, 156.85, 152.55, 139.99, 137.22, 135.78, 133.45, 130.87, 130.19, 129.76, 129.56, 128.42, 128.09, 127.75, 127.32, 126.59, 123.01, 122.77, 122.73, 122.65, 114.31, 111.83, 55.45; HRMS (m/z): $[M+H]^+$ calcd for $C_{32}H_{23}O_3$, 455.1642; found, 455.1640.

(8-Methyl-2,4-diphenyldibenzo[*b,d*]furan-1-yl)(phenyl)methanone (Figure 2, compound 3da)

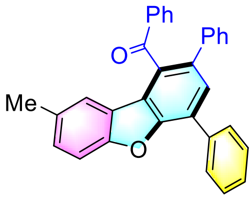

mp: 197–198 °C; TLC (ethyl acetate:petroleum ether, 1:20 v/v); R_f = 0.6; white solid; ^{1}H NMR (400 MHz, $CDCl_3$): δ 7.99 (d, J = 7.2 Hz, 2H), 7.74 (d, J = 7.2 Hz, 2H), 7.67 (s, 1H), 7.60–7.55 (m, 2H), 7.50–7.45 (m, 2H), 7.40–7.36 (m, 3H), 7.29–7.27 (m, 1H), 7.26–7.21 (m, 3H), 7.20–7.14 (m, 2H), 2.35 (s, 3H); ^{13}C{^{1}H} NMR (100 MHz, $CDCl_3$): δ 198.08, 155.28, 152.93, 139.96, 137.33, 135.82, 135.62, 133.36, 132.57, 131.41, 129.73, 129.58, 129.00, 128.81, 128.49, 128.37, 128.30, 128.26, 127.28, 126.81, 122.78, 122.65, 122.46, 111.34, 21.38; HRMS (m/z): $[M+H]^+$ calcd. for $C_{32}H_{23}O_2$, 439.1693; found, 439.1720.

(8-Methyl-2-phenyl-4-(p-tolyl)dibenzo[*b,d*]furan-1-yl)(phenyl)methanone (Figure 2, compound 3ea)

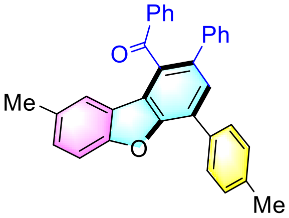

mp: 220–221°C; TLC (ethyl acetate:petroleum ether, 1:20 v/v) R_f = 0.4; light yellow solid; ^{1}H NMR (400 MHz, $CDCl_3$): δ 7.91–7.87 (m, 2H), 7.76–7.72 (m, 2H), 7.65 (s, 1H), 7.48 (d, J = 8.4 Hz, 1H), 7.43–7.28 (m, 7H), 7.26–7.12 (m, 5H), 2.47 (s, 3H), 2.35 (s, 3H); ^{13}C{^{1}H} NMR (100 MHz, $CDCl_3$): δ 198.18, 155.26, 152.93, 140.04, 138.27, 137.35, 135.61, 133.35, 132.87, 132.51, 131.10, 129.75, 129.58, 129.56, 128.97, 128.86, 128.37, 128.29, 128.25, 127.25, 126.84, 122.71, 122.45, 111.34, 21.40, 21.38; HRMS (m/z): $[M+H]^+$ calcd. for $C_{33}H_{25}O_2$, 453.1849; found, 453.1844.

(4-(4-Methoxyphenyl)-8-methyl-2-phenyldibenzo[*b,d*]furan-1-yl)(phenyl)methanone (Figure 2, compound 3fa)

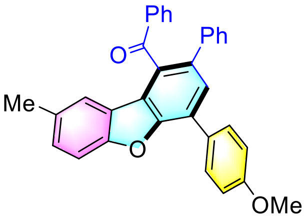

mp: 189–190 °C; TLC (ethyl acetate: petroleum ether, 1:20 v/v); R_f = 0.4; light yellow solid ; ^{1}H NMR (400 MHz, $CDCl_3$): δ 7.97–7.92 (m, 2H), 7.75–7.71 (m, 2H), 7.63 (s, 1H), 7.49 (d, J = 8.4 Hz, 1H), 7.44–7.35 (m, 4H), 7.29–7.26 (m, 1H), 7.26–7.18 (m, 4H), 7.18–7.14 (m, 1H), 7.13–7.09 (m, 2H), 3.92 (s, 3H), 2.35 (s, 3H); ^{13}C{^{1}H} NMR (100 MHz, $CDCl_3$): δ 198.18, 159.75, 155.24, 152.82, 140.07, 137.39, 135.66, 133.33, 132.51, 130.79, 130.18, 129.74, 129.57, 128.95, 128.35, 128.25, 128.17, 128.01, 127.25, 126.52, 122.72, 122.71, 122.48, 114.28, 111.31, 55.45, 21.40; HRMS (m/z): $[M+H]^+$ calcd. for $C_{33}H_{25}O_3$, 469.1798; found, 469.1802.

(4-(4-Chlorophenyl)-8-methyl-2-phenyldibenzo[*b,d*]furan-1-yl)(phenyl)methanone (Figure 2, compound 3ga)

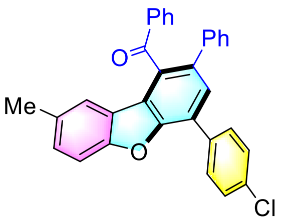

mp: 214–215 °C; TLC (ethyl acetate:petroleum ether, 1:20 v/v); R_f = 0.5; white solid ;^{1}H NMR (400 MHz, $CDCl_3$): δ 7.91–7.82 (m, 2H), 7.78–7.61 (m, 4H), 7.55–7.47 (m, 2H), 7.44–7.33 (m, 5H), 7.30–7.26 (m, 2H), 7.24–7.16 (m, 3H), 2.48 (s, 3H); $^{13}C\{^1H\}$ NMR (100 MHz, $CDCl_3$): δ 197.65, 155.60, 139.71, 138.56, 137.04, 136.28, 133.56, 132.44, 131.20, 130.72, 129.75, 129.63, 129.53, 129.19, 128.84, 128.43, 128.34, 127.46, 127.24, 125.37, 124.78, 121.81, 115.95, 113.33, 21.39; HRMS (m/z): $[M+H]^+$ calcd. for $C_{32}H_{22}ClO_2$, 473.1303; found, 473.1295.

(8-Chloro-2-phenyl-4-(*p*-tolyl)dibenzo[*b,d*]furan-1-yl)(phenyl)methanone (Figure 2, compound 3ha)

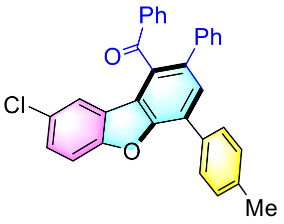

mp: 221–222 °C; TLC (ethyl acetate:petroleum ether, 1:20 v/v), R_f = 0.5;white solid;^{1}H NMR (400 MHz, $CDCl_3$): δ 7.88 (d, J = 8.0 Hz, 2H), 7.76–7.68 (m, 3H), 7.59–7.51 (m, 2H), 7.44–7.34 (m, 6H), 7.28–7.26 (m, 1H), 7.25–7.10 (m, 4H), 2.48 (s, 3H); $^{13}C\{^1H\}$ NMR (100 MHz, $CDCl_3$): δ 197.67, 155.20, 153.31, 139.71, 138.55, 137.02, 136.22, 133.57, 132.46, 131.21, 129.75, 129.63, 129.54, 129.15, 128.84, 128.50, 128.44, 128.35, 127.96, 127.46, 127.25, 124.20, 122.38, 121.97, 112.86, 21.39; HRMS (m/z): $[M+H]^+$ calcd. for $C_{32}H_{22}ClO_2$, 473.1303; found, 473.1299.

(8-Chloro-4-(4-methoxyphenyl)-2-phenyldibenzo[*b,d*]furan-1-yl)(phenyl)methanone (Figure 2, compound 3ia)

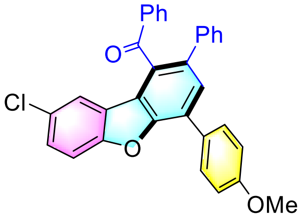

mp: 230–231 °C; TLC (ethyl acetate:petroleum ether, 1:20 v/v); R_f = 0.5; light yellow solid; ^{1}H NMR (400 MHz, $CDCl_3$): δ 7.93 (d, J = 8.8 Hz, 2H), 7.74–7.68 (m, 3H), 7.60–7.52 (m, 2H), 7.43–7.35 (m, 4H), 7.28–7.26 (m, 1H), 7.26–7.18 (m, 3H), 7.18–7.13 (m, 1H), 7.13–7.08 (m, 2H), 3.92 (s, 3H); $^{13}C\{^1H\}$ NMR (100 MHz, $CDCl_3$): δ 197.67, 159.92, 155.18, 153.21, 139.76, 137.07, 136.27, 133.55, 130.89, 130.17, 129.75, 129.54, 128.87, 128.50, 128.42, 128.34, 127.95, 127.73, 127.46, 126.94, 124.23, 122.41, 121.99, 114.36, 112.83, 55.46; HRMS (m/z): $[M+H]^+$ calcd. for $C_{32}H_{22}ClO_3$, 489.1252; found, 489.1259.

(8-Chloro-2-phenyl-4-(*p*-tolyl)dibenzo[*b,d*]furan-1-yl)(phenyl)methanone (Figure 2, compound 3ja)

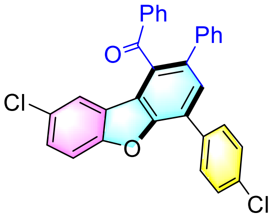

mp: 197–198 °C; TLC (ethyl acetate:petroleum ether, 1:20 v/v); R_f = 0.6; light yellow solid; ^{1}H NMR (400 MHz, $CDCl_3$): δ 7.95–7.88 (m, 2H), 7.74–7.66 (m, 3H), 7.59–7.50 (m, 4H), 7.45–7.39 (m, 2H), 7.36–7.26 (m, 4H), 7.25–7.17 (m, 3H); $^{13}C\{^1H\}$ NMR (100 MHz, $CDCl_3$): δ 197.41, 155.17, 139.45, 136.88, 136.31, 134.61, 133.80, 133.67, 131.86, 130.22, 129.72, 129.50, 129.12, 129.05, 128.71, 128.47, 128.40, 128.17, 127.59, 125.93, 124.04, 122.43, 112.85; HRMS (m/z): $[M+H]^+$ calcd. for $C_{31}H_{19}Cl_2O_2$, 493.0757; found, 493.0767.

(8-Chloro-4-(2-methoxyphenyl)-2-phenyldibenzo[*b,d*]furan-1-yl)(phenyl)methanone (Figure 2, compound 3ka)

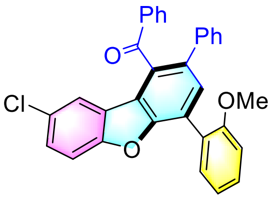

mp: 177–178 °C; TLC (ethyl acetate:petroleum ether, 1:20 v/v); R_f = 0.5; white solid; ^{1}H NMR (400 MHz, CDCl$_3$): δ 7.77–7.71 (m, 2H), 7.66 (s, 1H), 7.60–7.54 (m, 2H), 7.49–7.40 (m, 3H), 7.38–7.33 (m, 3H), 7.29–7.27 (m, 1H), 7.25–7.22 (m, 1H), 7.21–7.17 (m, 2H), 7.17–7.11 (m, 3H), 3.87 (s, 3H); ^{13}C{^{1}H} NMR (100 MHz, CDCl$_3$): δ 197.80, 157.12, 155.19, 153.93, 139.80, 137.06, 135.47, 133.51, 131.59, 131.28, 130.07, 129.80, 129.59, 128.40, 128.28, 128.26, 127.72, 127.30, 124.66, 124.51, 124.40, 122.30, 121.36, 120.83, 112.71, 111.58, 55.78; HRMS (m/z): [M+H]$^+$ calcd. for $C_{32}H_{22}ClO_3$, 489.1252; found, 489.1251.

(8-Bromo-2,4-diphenyldibenzo[*b,d*]furan-1-yl)(phenyl)methanone (Figure 2, compound 3la)

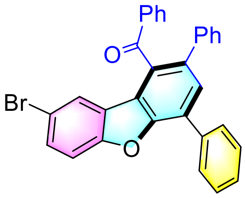

mp: 148–149 °C; TLC (ethyl acetate:petroleum ether, 1:20 v/v), R_f = 0.6; white solid; ^{1}H NMR (400 MHz, CDCl$_3$): δ 7.99–7.93 (m, 2H), 7.75–7.68 (m, 4H), 7.60–7.53 (m, 3H), 7.52–7.47 (m, 2H), 7.42–7.35 (m, 3H), 7.29–7.27 (m, 1H), 7.25–7.11 (m, 4H); ^{13}C{^{1}H} NMR (100 MHz, CDCl$_3$): δ 197.56, 155.62, 153.12, 139.63, 137.01, 136.30, 135.39, 133.59, 130.78, 129.74, 129.54, 129.41, 128.99, 128.90, 128.54, 128.45, 128.36, 127.50, 127.21, 125.39, 124.75, 121.87, 116.01, 113.33; HRMS (m/z): [M+H]$^+$ calcd. for $C_{31}H_{20}BrO_2$, 581.9825; found, 581.9818.

(8-Bromo-2-phenyl-4-(*p*-tolyl)dibenzo[*b,d*]furan-1-yl)(phenyl)methanone (Figure 2, compound 3ma)

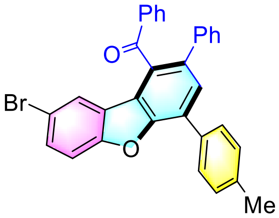

mp: 226–227 °C; TLC (ethyl acetate:petroleum ether, 1:20 v/v);, R_f = 0.5; white solid ; ^{1}H NMR (400 MHz, $CDCl_3$): δ 7.95–7.92 (m, 2H), 7.75–7.71 (m, 2H), 7.63 (s, 1H), 7.56–7.47 (m, 3H), 7.44–7.40 (m, 1H), 7.38–7.34 (m, 3H), 7.31–7.24 (m, 2H), 7.25–7.20 (m, 3H), 7.20–7.16 (m, 1H), 2.35 (s, 3H); $^{13}C\{^1H\}$ NMR (100 MHz, $CDCl_3$): δ 197.92, 155.23, 152.74, 139.77, 137.21, 135.71, 134.34, 134.21, 133.45, 132.74, 131.74, 130.25, 129.72, 129.54, 129.18, 129.04, 128.41, 128.31, 128.16, 127.39, 125.52, 122.91, 122.53, 122.50, 111.32, 21.40; HRMS (m/z): $[M+H]^+$ calcd. for $C_{32}H_{22}BrO_2$, 517.0798; found, 517.0807.

(8-Bromo-4-(4-methoxyphenyl)-2-phenyldibenzo[*b,d*]furan-1-yl)(phenyl)methanone (Figure 2, compound 3na)

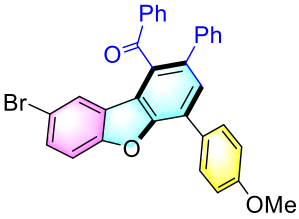

mp: 230–231 °C; TLC (ethyl acetate:petroleum ether, 1:20 v/v), R_f = 0.5; light yellow solid ;^{1}H NMR (400 MHz, $CDCl_3$): δ 7.95–7.90 (m, 2H), 7.78–7.68 (m, 4H), 7.59–7.49 (m, 2H), 7.49–7.30 (m, 4H), 7.29–7.27 (m, 1H), 7.24–7.20 (m, 2H), 7.18–7.07 (m, 3H), 3.92 (s, 3H); $^{13}C\{^1H\}$ NMR (100 MHz, $CDCl_3$): δ 197.65, 159.93, 155.59, 153.02, 139.76, 137.10, 136.34, 133.54, 130.90, 130.70, 130.18, 129.75, 129.54, 128.91, 128.42, 128.34, 127.71, 127.46, 126.92, 125.40, 124.82, 121.83, 115.96, 114.62, 114.36, 113.31, 55.46; HRMS (m/z): $[M+H]^+$ calcd. for $C_{32}H_{22}BrO_3$, 533.0747; found, 533.0739.

(6-Bromo-2-phenyl-4-(*p*-tolyl)dibenzo[*b,d*]furan-1-yl)(phenyl)methanone (Figure 2, compound 3oa)

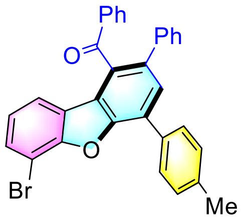

mp: 197–198 °C; TLC (ethyl acetate:petroleum ether, 1:20 v/v); R_f = 0.5; white solid; ^{1}H NMR (400 MHz, $CDCl_3$): δ 8.00 (d, J = 8.0 Hz, 2H), 7.82–7.71 (m, 3H), 7.64–7.55 (m, 2H), 7.48–7.37 (m, 5H), 7.34–7.26 (m, 3H), 7.25–7.06 (m, 3H), 2.51 (s, 2H); ^{13}C{^{1}H} NMR (125 MHz, $CDCl_3$): δ 191.27, 158.61, 157.81, 156.20, 138.45, 128.93, 126.17, 123.89, 123.64, 121.44, 121.37, 119.83, 112.18, 111.95, 79.88, 44.38; HRMS (m/z): $[M+H]^+$ calcd. for $C_{32}H_{22}BrO_2$, 517.0798; found, 517.0792.

(6,8-Di-*tert*-butyl-2-phenyl-4-(*p*-tolyl)dibenzo[*b,d*]furan-1-yl)(phenyl)methanone (Figure 2, compound 3pa)

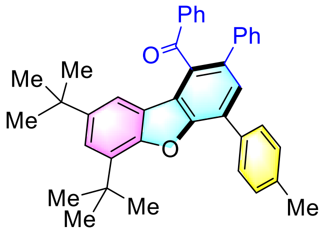

mp: 231–232 °C; TLC(ethyl acetate:petroleum ether, 1:20 v/v); R_f = 0.4; white solid;. ^{1}H NMR (400 MHz, $CDCl_3$): δ 7.97–7.92 (m, 2H), 7.87–7.82 (m, 2H), 7.69 (s, 1H), 7.47–7.37 (m, 6H), 7.36–7.26 (m, 4H), 7.25–7.19 (m, 2H), 2.48 (s, 3H), 1.56 (s, 9H), 1.20 (s, 9H); ^{13}C{^{1}H} NMR (100 MHz, $CDCl_3$): δ 197.91, 153.22, 152.28, 145.59, 140.29, 138.03, 137.50, 135.43, 134.01, 133.39, 133.01, 131.26, 129.80, 129.57, 129.49, 128.68, 128.59, 128.27, 127.98, 127.19, 126.19, 122.85, 122.45, 122.24, 116.84, 34.87, 34.60, 31.66, 29.95, 21.37; HRMS (m/z): $[M+H]^+$ calcd. for $C_{40}H_{39}O_2$, 551.2945; found, 551.2947.

(6-Bromo-8-chloro-4-(4-methoxyphenyl)-2-phenyldibenzo[*b,d*]furan-1-yl)(phenyl)methanone (Figure 2, compound 3qa)

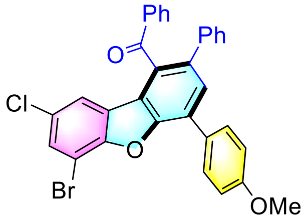

mp: 206–207 °C; TLC (ethyl acetate:petroleum ether, 1:20 v/v); R_f =0.5; light yellow solid;^{1}H NMR (400 MHz, $CDCl_3$): δ 8.03–7.97 (m, 2H), 7.75–7.67 (m, 3H), 7.62–7.53 (m, 2H), 7.43–7.39 (m, 1H), 7.37–7.26 (m, 3H), 7.25–7.19 (m, 3H), 7.19–7.15 (m, 1H), 7.14–7.10 (m, 2H), 3.92 (s, 3H); $^{13}C\{^1H\}$ NMR (100 MHz, $CDCl_3$): δ 197.35, 160.04, 152.93, 152.66, 139.54, 136.95, 136.86, 133.65, 131.06, 130.31, 130.11, 129.71, 129.51, 129.23, 128.96, 128.45, 128.39, 127.59, 127.24, 127.14, 124.99, 122.17, 121.52, 114.44, 104.90, 55.46; HRMS (m/z): $[M+H]^+$ calcd. for $C_{32}H_{21}BrClO_3$, 567.0357; found, 567.0352.

(8-Bromo-4-(cyclohex-1-en-1-yl)-2-phenyldibenzo[*b,d*]furan-1-yl)(phenyl)methanone (Figure 2, compound 3ra)

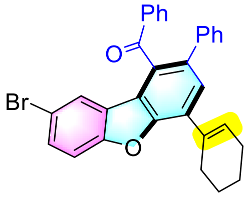

mp: 167–168 °C; TLC (ethyl acetate:petroleum ether, 1:20 v/v); R_f = 0.4; white solid;^{1}H NMR (400 MHz, $CDCl_3$): δ 7.71–7.64 (m, 3H), 7.53–7.46 (m, 3H), 7.41–7.38 (m, 1H), 7.33–7.29 (m, 2H), 7.25–7.19 (m, 3H), 7.19–7.06 (m, 2H), 6.71–6.60 (m, 1H), 2.72–2.62 (m, 2H), 2.42–2.33 (m, 2H), 1.92–1.86 (m, 2H); $^{13}C\{^1H\}$ NMR (100 MHz, $CDCl_3$): δ 197.77, 155.36, 153.02, 139.94, 137.18, 135.87, 133.42, 132.81, 130.56, 130.45, 129.71, 129.52, 129.41, 128.35, 128.24, 127.64, 127.30, 125.28, 124.74, 121.37, 115.78, 113.18, 28.10, 26.08, 23.00, 22.04; HRMS (m/z): $[M+H]^+$ calcd. for $C_{31}H_{23}BrO_2$, 507.0954; found, 507.0953.

(8-Bromo-2-phenyl-4-(thiophen-3-yl)dibenzo[*b,d*]furan-1-yl)(phenyl)methanone (Figure 2, compound 3sa)

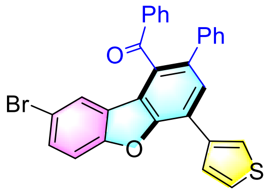

mp: 236–237 °C; TLC (ethyl acetate:petroleum ether, 1:20 v/v); R_f = 0.5; white solid; ^{1}H NMR (400 MHz, $CDCl_3$): δ 8.22–8.14 (m, 1H), 7.82 (s, 1H), 7.80 (dd, J = 5.2 Hz, 1.2 Hz, 1H), 7.74–7.66 (m, 3H), 7.58–7.55 (m, 1H), 7.54–7.50 (m, 2H), 7.43–7.34 (m, 3H), 7.27 (s, 1H), 7.25–7.20 (m, 3H), 7.20–7.16 (m, 1H); $^{13}C\{^1H\}$ NMR (100 MHz, $CDCl_3$): δ 197.49, 155.54, 139.65, 137.08, 136.33, 135.55, 133.54, 131.07, 130.81, 129.70, 129.53, 128.41, 128.35, 127.94, 127.51, 127.08, 126.13, 125.45, 124.81, 124.72, 122.00, 121.83, 116.11, 113.28; HRMS (m/z): $[M+H]^+$ calcd. for $C_{29}H_{18}BrO_2S$, 509.0205; found, 509.0203.

(4-Phenyl-2-(*o*-tolyl)dibenzo[b,d]furan-1-yl)(*o*-tolyl)methanone (Figure 3, compound 3ab)

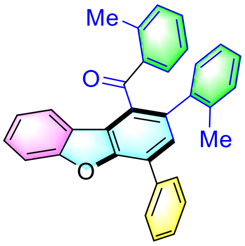

mp: 137–138 °C; TLC (ethyl acetate:petroleum ether, 1:20 v/v); R_f = 0.6; white solid; ^{1}H NMR (400 MHz, $CDCl_3$): δ 7.97 (d, J = 7.6 Hz, 2H), 7.79 (d, J = 8.0 Hz, 1H), 7.65 (d, J = 8.4 Hz, 1H), 7.56 (d, J = 7.6 Hz, 2H), 7.53 – 7.43 (m, 3H), 7.26 – 7.18 (m, 3H), 7.12 – 7.01 (m, 3H), 7.01 – 6.93 (m, 3H), 2.27 (s, 3H), 2.06 (s, 3H); $^{13}C\{^1H\}$ NMR (100 MHz, $CDCl_3$): δ 199.98, 156.92, 152.52, 138.93, 138.81, 138.28, 136.24, 135.74, 135.29, 134.07, 133.15, 131.58, 131.36, 130.58, 130.47, 129.83, 129.00, 128.80, 128.63, 128.30, 127.88, 127.65, 126.49, 125.09, 125.00, 123.10, 122.91, 122.77, 111.84, 20.43, 20.26; HRMS (m/z): $[M+H]^+$ calcd. for $C_{33}H_{25}O_2$, 453.1849; found, 453.1842.

(4-Phenyl-2-(*m*-tolyl)dibenzo[*b,d*]furan-1-yl)(*m*-tolyl)methanone (Figure 3, compound 3ac)

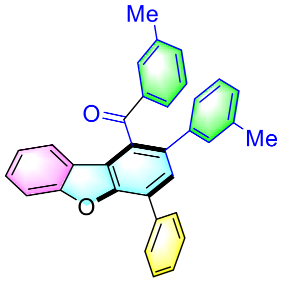

mp: 156–157 °C; TLC (ethyl acetate:petroleum ether, 1:20 v/v); R_f = 0.5; white solid; ^{1}H NMR (400 MHz, $CDCl_3$): δ 8.01 (d, *J* = 7.6 Hz, 2H), 7.71 (s, 1H), 7.64–7.58 (m, 4H), 7.58–7.50 (m, 2H), 7.50–7.41 (m, 2H), 7.25–7.22 (m, 1H), 7.22–7.13 (m, 4H), 7.13–7.06 (m, 1H), 7.02–6.95 (m, 1H), 2.28 (s, 3H), 2.25 (s, 3H); $^{13}C\{^1H\}$ NMR (100 MHz, $CDCl_3$): δ 198.07, 156.87, 152.61, 139.90, 138.13, 137.83, 137.20, 135.86, 135.83, 134.24, 131.68, 130.34, 130.06, 129.01, 128.83, 128.69, 128.31, 128.28, 128.13, 128.01, 127.73, 127.18, 126.74, 126.63, 123.01, 122.80, 122.75, 122.71, 111.80, 21.33, 21.20; HRMS (m/z): $[M+H]^+$ calcd. for $C_{33}H_{25}O_2$, 453.1849; found, 453.1841.

(4-Phenyl-2-(*p*-tolyl)dibenzo[*b,d*]furan-1-yl)(*p*-tolyl)methanone (Figure 3, compound 3ad)

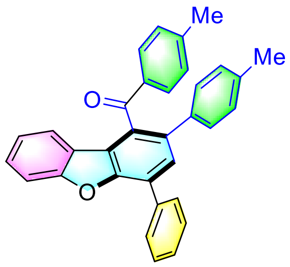

mp: 190–191 °C; TLC (ethyl acetate:petroleum ether, 1:20 v/v); R_f = 0.5; white solid; ^{1}H NMR (400 MHz, $CDCl_3$): δ 7.99 (d, *J* = 8.0 Hz, 2H), 7.74–7.66 (m, 3H), 7.62–7.55 (m, 3H), 7.53–7.46 (m, 2H), 7.44–7.39 (m, 1H), 7.32–7.26 (m, 2H), 7.18–7.13 (m, 1H), 7.13–7.01 (m, 4H), 2.32 (s, 3H), 2.28 (s, 3H); $^{13}C\{^1H\}$ NMR (100 MHz, $CDCl_3$): δ197.56, 156.80, 152.46, 144.55, 137.01, 135.86, 135.45, 134.67, 131.75, 129.98, 129.33, 129.07, 128.99, 128.82, 128.27, 127.63, 122.99, 122.78, 122.59, 122.54, 111.79, 21.78, 21.12; HRMS (m/z): $[M+H]^+$ calcd. for $C_{33}H_{25}O_2$, 453.1849; found, 453.1851.

(4-Ethylphenyl)(2-(4-ethylphenyl)-4-phenyldibenzo[*b,d*]furan-1-yl)methanone (Figure 3, compound 3ae)

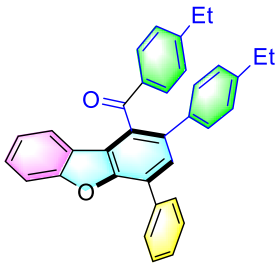

mp: 120–121 °C; TLC (ethyl acetate:petroleum ether, 1:20 v/v); R_f = 0.5; light yellow solid;^{1}H NMR (400 MHz, $CDCl_3$): δ 8.02–7.97 (m, 2H), 7.74–7.67 (m, 3H), 7.63–7.56 (m, 3H), 7.56–7.54 (m, 1H), 7.50–7.46 (m, 1H), 7.44–7.40 (m, 1H), 7.33–7.28 (m, 2H), 7.19–7.14 (m, 1H), 7.12–7.09 (m, 2H), 7.08–7.05 (m, 2H), 2.58 (q, *J* = 7.6 Hz, 4H), 1.17 (t, *J* =7.6 Hz, 6H); $^{13}C\{^1H\}$ NMR (100 MHz, $CDCl_3$): δ197.62, 156.83, 152.49, 150.44, 143.27, 137.26, 135.88, 135.65, 135.01, 131.81, 130.04, 129.48, 128.99, 128.81, 128.25, 127.99, 127.78, 127.62, 126.60, 122.96, 122.81, 122.68, 122.62, 111.76, 28.96, 28.44, 15.42, 14.95; HRMS (m/z): $[M+H]^+$ calcd. for $C_{35}H_{29}O_2$, 481.2162; found, 481.2156.

(4-Methoxyphenyl)(2-(4-methoxyphenyl)-4-phenyldibenzo[*b,d*]furan-1-yl)methanone (Figure 3, compound 3af)

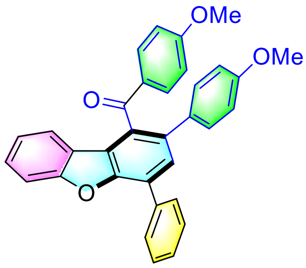

mp: 189–190 °C; TLC (ethyl acetate:petroleum ether, 1:20 v/v); R_f = 0.54; light yellow solid;^{1}H NMR (400 MHz, $CDCl_3$): δ 8.01–7.95 (m, 2H), 7.80–7.75 (m, 2H), 7.66 (s, 1H), 7.62–7.54 (m, 4H), 7.49–7.45 (m, 1H), 7.44–7.39 (m, 1H), 7.36–7.32 (m, 2H), 7.19–7.14 (m, 1H), 6.82–6.74 (m, 4H), 3.79 (s, 3H), 3.76 (s, 3H); $^{13}C\{^1H\}$ NMR (100 MHz, $CDCl_3$): δ 196.45, 163.88, 158.88, 156.81, 152.40, 135.89, 135.01, 132.45, 132.23, 131.74, 130.55, 130.19, 128.97, 128.81, 128.74, 128.24, 127.60, 126.49, 122.99, 122.81, 122.57, 122.52, 113.83, 113.79, 111.77, 55.43, 55.22; HRMS (m/z): $[M+H]^+$ calcd. for $C_{33}H_{25}O_4$, 485.1747; found, 485.1754.

(3-Fluorophenyl)(2-(3-fluorophenyl)-4-phenyldibenzo[*b,d*]furan-1-yl)methanone (Figure 3, compound 3ag)

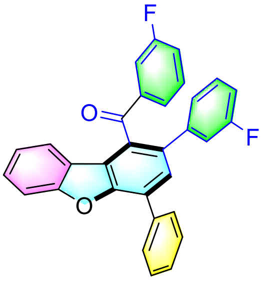

mp: 170–171 °C; TLC (ethyl acetate:petroleum ether, 1:20 v/v); R_f = 0.6; white solid; ^{1}H NMR (400 MHz, $CDCl_3$): δ 8.04–7.99 (m, 2H), 7.70 (s, 1H), 7.67–7.59 (m, 4H), 7.54–7.46 (m, 4H), 7.30–7.27 (m, 1H), 7.26–7.19 (m, 2H), 7.19–7.10 (m, 3H), 6.95–6.89 (m, 1H); ^{13}C{^{1}H} NMR (100 MHz, $CDCl_3$): δ 196.37, 162.6 (d, $J_{C\text{-}F}$ = 245 Hz), 162.51(d, $J_{C\text{-}F}$ = 245.5 Hz), 156.98, 152.89, 141.88 (d, $J_{C\text{-}F}$ = 7.8 Hz), 139.20 (d, $J_{C\text{-}F}$ = 6.2 Hz), 135.42, 134.44, 130.78, 130.24 (d, $J_{C\text{-}F}$ = 7.5 Hz), 129.96 (d, $J_{C\text{-}F}$ = 8.3 Hz), 129.02, 128.91, 128.58, 128.44, 128.16, 127.43, 125.65(d, $J_{C\text{-}F}$ = 2.7 Hz), 125.46(d, J = 2.8 Hz), 123.25, 123.01, 122.54, 122.43, 120.72(d, $J_{C\text{-}F}$ = 21.3 Hz), 116.69(d, $J_{C\text{-}F}$ = 23.8 Hz), 115.95(d, $J_{C\text{-}F}$ = 22.3 Hz), 114.47(d, $J_{C\text{-}F}$ = 20.9 Hz), 112.00; HRMS (m/z): $[M+H]^+$ calcd. for $C_{31}H_{19}F_2O_2$, 461.1348; found, 461.1343.

(2-Chlorophenyl)(2-(2-chlorophenyl)-4-phenyldibenzo[*b,d*]furan-1-yl)methanone (Figure 3, compound 3ah)

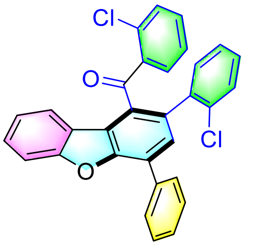

mp 165–166 °C; TLC (ethyl acetate:petroleum ether, 1:20 v/v); R_f = 0.6; white solid; ^{1}H NMR (400 MHz, $CDCl_3$): δ 8.14–8.03 (m, 3H), 7.77–7.73 (m, 1H), 7.68–7.62 (m, 3H), 7.61–7.53 (m, 2H), 7.42–7.27 (m, 4H), 7.25–7.14 (m, 4H), 7.14–7.06 (m, 1H); ^{13}C{^{1}H} NMR (100 MHz, $CDCl_3$): δ 196.21, 157.09, 153.12, 138.11, 135.46, 133.62, 133.17, 132.71, 132.52, 132.28, 132.14, 130.97, 130.40, 129.40, 129.31, 129.14, 129.03, 128.81, 128.52, 128.20, 127.52, 126.54, 126.23, 123.82, 123.55, 123.12, 122.81, 111.82, 112.00; HRMS (m/z): $[M+H]^+$ calcd. for $C_{31}H_{19}Cl_2O_2$, 493.0757; found, 493.0752.

(3-Chlorophenyl)(2-(3-chlorophenyl)-4-phenyldibenzo[*b,d*]furan-1-yl)methanone (Figure 3, compound 3ai)

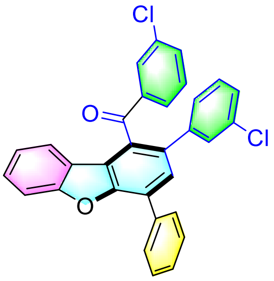

mp: 171–172 °C; TLC (ethyl acetate:petroleum ether, 1:20 v/v); R_f = 0.6; light yellow solid; ^{1}H NMR (400 MHz, $CDCl_3$): δ 8.04–7.97 (m, 2H), 7.69 (s, 1H), 7.66–7.57 (m, 4H), 7.55–7.44 (m, 4H), 7.31–7.25 (m, 1H), 7.25–7.20 (m, 2H), 7.19–7.09 (m, 3H), 6.96–6.84 (m, 1H); ^{13}C{^{1}H} NMR (100 MHz, $CDCl_3$): δ 196.41, 163.74, 161.29, 156.98, 152.90, 141.85, 139.24, 135.42, 134.46, 130.78, 130.29, 129.02, 128.92, 128.58, 128.17, 127.44, 125.67, 125.48, 123.25, 122.55, 120.84, 120.62, 116.57, 116.06, 114.59; HRMS (m/z): $[M+H]^+$ calcd. for $C_{31}H_{19}Cl_2O_2$, 493.0757; found, 493.0753.

(2-Bromophenyl)(2-(2-bromophenyl)-4-phenyldibenzo[*b,d*]furan-1-yl)methanone (Figure 3, compound 3aj)

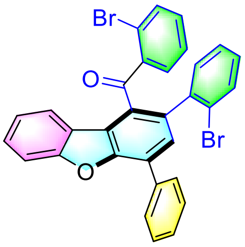

mp: 177–178 °C; TLC (ethyl acetate:petroleum ether, 1:20 V/V); R_f = 0.5; white solid; ^{1}H NMR (400 MHz, $CDCl_3$): δ 8.04–7.96 (m, 3H), 7.66 (d, J = 8.4 Hz, 1H), 7.59–7.53 (m, 3H), 7.53–7.44 (m, 2H), 7.40–7.35 (m, 2H), 7.34–7.26 (m, 2H), 7.25–7.17 (m, 2H), 7.12–6.99 (m, 3H); ^{13}C{^{1}H} NMR (100 MHz, $CDCl_3$): δ 196.71, 157.11, 153.07, 139.94, 139.78, 135.46, 135.24, 133.76, 132.67, 132.29, 132.21, 132.10, 131.30, 129.46, 129.14, 129.07, 128.79, 128.51, 128.18, 127.51, 127.22, 126.85, 124.10, 123.65, 123.58, 123.10, 122.84, 120.82, 111.79; HRMS (m/z) $[M+H]^+$ calcd. for $C_{31}H_{19}Br_2O_2$, 580.9746; found, 580.9741.

(3-Bromophenyl)(2-(3-bromophenyl)-4-phenyldibenzo[*b,d*]furan-1-yl)methanone (Figure 3, compound 3ak)

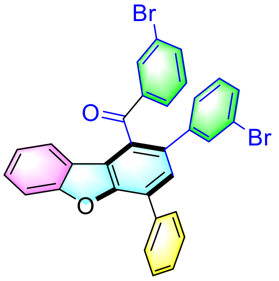

mp: 181–182 °C; TLC (ethyl acetate:petroleum ether, 1:20 v/v); R_f = 0.6; white solid; ^{1}H NMR (400 MHz, $CDCl_3$): δ 7.99 (d, J = 7.6 Hz, 2H), 7.87 (s, 1H), 7.67 (s, 1H), 7.65 (s, 1H), 7.64–7.60 (m, 3H), 7.59–7.52 (m, 3H), 7.52–7.43 (m, 2H), 7.38–7.26 (m, 2H), 7.26–7.05 (m, 3H); ^{13}C{^{1}H} NMR (100 MHz, $CDCl_3$): δ 196.14, 157.00, 152.98, 141.77, 138.85, 136.38, 135.36, 134.32, 132.43, 132.36, 130.59, 130.10, 129.84, 129.04, 128.92, 128.63, 128.40, 128.24, 127.60, 123.28, 123.15, 122.82, 122.64, 122.55, 122.40, 112.01; HRMS (m/z): $[M+H]^+$ calcd. for $C_{31}H_{19}Br_2O_2$, 580.9746; found, 580.9744.

(4-Bromophenyl)(2-(4-bromophenyl)-4-phenyldibenzo[*b,d*]furan-1-yl)methanone (Figure 3, compound 3al)

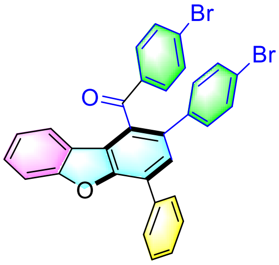

mp: 224–225 °C; TLC (ethyl acetate:petroleum ether. 1:20 v/v); R_f = 0.6; white solid; ^{1}H NMR (400 MHz, $CDCl_3$): δ 7.97 (d, J = 8.0 Hz, 2H), 7.65–7.56 (m, 6H), 7.54–7.48 (m, 2H), 7.48–7.43 (m, 3H), 7.41–7.37 (m, 2H), 7.25–7.17 (m, 3H); ^{13}C{^{1}H} NMR (100 MHz, $CDCl_3$): δ 196.66, 156.92, 152.76, 138.60, 135.71, 135.42, 134.26, 132.07, 131.62, 131.11, 131.03, 130.73, 129.28, 128.98, 128.91, 128.55, 128.47, 128.11, 127.28, 123.25, 122.83, 122.42, 122.37, 122.04, 112.00; HRMS (m/z): $[M+H]^+$ calcd. for $C_{31}H_{19}Br_2O_2$, 580.9746; found, 580.9755.

(4-Phenyl-2-(4-(trifluoromethyl)phenyl)dibenzo[*b,d*]furan-1-yl)(4-(trifluoromethyl)phenyl)methanone (Figure 3 and Figure 4, compound 3am)

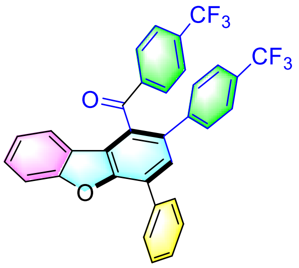

mp: 212–213 °C; TLC (ethyl acetate:petroleum ether, 1:20 v/v); R_f = 0.6; white solid; ^{1}H NMR (400 MHz, $CDCl_3$): δ 8.03–7.96 (m, 2H), 7.83 (d, J = 8.0 Hz, 2H), 7.69 (s, 1H), 7.65 (d, J = 8.0 Hz, 1H), 7.63–7.59 (m, 2H), 7.59–7.52 (m, 4H), 7.52–7.46 (m, 5H), 7.26–7.20 (m, 1H); ^{13}C{^{1}H} NMR (100 MHz, $CDCl_3$): δ 196.58, 157.03, 153.02, 143.26, 139.81, 135.23, 134.97, 134.64, 134.40, 130.65, 129.92, 129.86, 129.01, 128.96, 128.71, 128.47, 128.37, 127.74, 125.68, 125.64, 125.39, 125.35, 123.37, 123.14, 122.49, 122.25, 112.10; HRMS (m/z): $[M+H]^+$ calcd. for $C_{33}H_{19}F_6O_2$, 561.12846; found, 561.1278.

Naphthalen-1-yl(2-(naphthalen-1-yl)-4-phenyldibenzo[*b,d*]furan-1-yl)methanone (Figure 3, compound 3an)

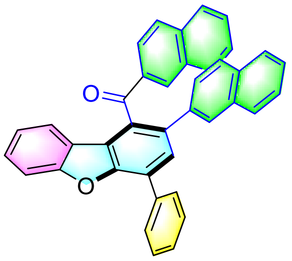

mp: 108–109 °C; TLC (ethyl acetate:petroleum ether, 1:20 v/v); R_f = 0.5; white solid; ^{1}H NMR (400 MHz, $CDCl_3$): δ 8.21–8.18 (s, 1H), 8.09–8.04 (m, 2H), 7.99–7.83 (m, 4H), 7.73–7.69 (m, 4H), 7.68–7.63 (m, 2H), 7.62–7.56 (m, 4H), 7.52–7.47 (m, 2H), 7.45–7.37 (m, 4H), 7.18–7.12 (m, 1H); ^{13}C{^{1}H} NMR (100 MHz, $CDCl_3$): δ 197.83, 156.92, 152.72, 137.39, 135.78, 135.58, 134.52, 133.12, 132.43, 132.35, 132.29, 131.91, 129.72, 129.12, 129.05, 128.90, 128.72, 128.65, 128.60, 128.41, 128.13, 128.11, 127.82, 127.73, 127.54, 127.33, 126.92, 126.61, 126.25, 126.19, 126.06, 124.42, 123.14, 122.93, 122.74, 122.61, 111.88; HRMS (m/z): $[M+H]^+$ calcd. for $C_{39}H_{25}O_2$, 525.1849; found, 525.1846.

(8-Bromo-2-(naphthalen-1-yl)-4-phenyldibenzo[*b,d*]furan-1-yl)(naphthalen-1-yl)methanone (Figure 3, compound 3ln)

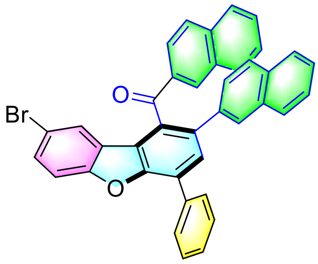

mp: 219–220 °C; TLC (ethyl acetate:petroleum ether, 1:20 v/v); R_f = 0.6; white solid; ^{1}H NMR (400 MHz, $CDCl_3$): δ 8.18–8.08 (s, 1H), 8.07–7.97 (m, 2H), 7.95–7.84 (m, 3H), 7.76–7.69 (m, 4H), 7.69–7.63 (m, 3H), 7.63–7.58 (m, 2H), 7.56–7.52 (m, 2H), 7.52–7.46 (m, 3H), 7.43–7.35 (m, 3H); ^{13}C{^{1}H} NMR (100 MHz, $CDCl_3$): δ 197.48, 155.66, 153.20, 137.13, 136.08, 135.78, 135.43, 134.35, 133.07, 132.40, 132.30, 132.26, 131.92, 130.81, 129.77, 129.64, 129.02, 128.95, 128.77, 128.70, 128.62, 128.59, 128.23, 128.09, 127.75, 127.53, 127.24, 127.16, 126.65, 126.33, 126.16, 125.32, 124.79, 124.35, 122.02, 116.08, 113.36; HRMS (m/z): [M+H]$^+$ calcd. for $C_{39}H_{24}BrO_2$, 603.0954; found, 603.0958.

(8-Bromo-4-(4-methoxyphenyl)-2-(*p*-tolyl)dibenzo[*b,d*]furan-1-yl)(*p*-tolyl)methanone (Figure 3, compound 3nd)

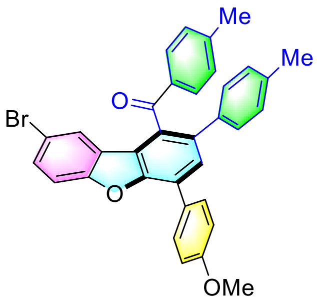

mp: 177–178 °C; TLC (ethyl acetate:petroleum ether, 1:20 v/v); R_f = 0.4; light yellow solid; ^{1}H NMR (400 MHz, $CDCl_3$): δ 7.93–7.89 (m, 2H), 7.70–7.63 (m, 4H), 7.53–7.45 (m, 2H), 7.28 (s, 1H), 7.21–6.87 (m, 7H), 3.91 (s, 3H), 2.32 (s, 3H), 2.27 (s, 3H); ^{13}C{^{1}H} NMR (100 MHz, $CDCl_3$): δ 197.19, 159.86, 155.51, 152.85, 144.61, 137.11, 136.89, 135.97, 134.57, 131.19, 130.50, 130.13, 129.97, 129.30, 129.09, 129.03, 127.86, 126.58, 125.32, 124.65, 121.57, 115.87, 114.33, 113.22, 55.44, 21.77, 21.11; HRMS (m/z): [M+H]$^+$ calcd. for $C_{34}H_{26}BrO_3$, 561.1060; found, 561.1067.

(4-Phenyl-2-(thiophen-2-yl)dibenzo[b,d]furan-1-yl)(thiophen-2-yl)methanone (Figure 3, compound 3ap)

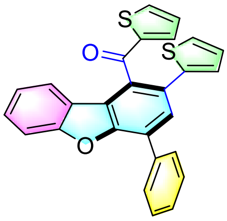

mp: 168-169 °C; TLC (ethyl acetate:petroleum ether, 1:20 v/v); R_f =0.6; white solid; ^{1}H NMR (400 MHz, $CDCl_3$): δ 8.03 – 7.92 (m, 2H), 7.81 (s, 1H), 7.70 (d, J = 7.6 Hz, 2H), 7.68 – 7.64 (m, 1H), 7.63 – 7.54 (m, 3H), 7.52 – 7.43 (m, 2H), 7.36 – 7.29 (m, 1H), 7.26 – 7.20 (m, 2H), 7.17 – 7.08 (m, 1H), 7.02 – 6.85 (m, 2H); $^{13}C\{^{1}H\}$ NMR (100 MHz, $CDCl_3$): δ 189.48, 156.87, 152.74, 144.33, 141.10, 135.55, 135.41, 131.11, 129.29, 129.00, 128.88, 128.83, 128.51, 128.33, 128.04, 127.77, 127.73, 127.17, 126.31, 123.28, 122.81, 122.53, 122.44, 111.91; HRMS (m/z): $[M+H]^+$ calcd. for $C_{27}H_{17}O_2S_2$, 437.0664; found, 437.0667.

(2-(4-Methoxyphenyl)-4-phenyldibenzo[*b,d*]furan-1-yl)(4-(trifluoromethyl)phenyl)methanone (Figure 4, compound 3afm)

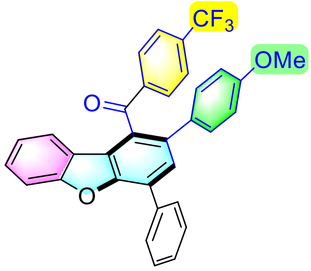

mp: 139–140 °C; TLC (ethyl acetate:petroleum ether, 1:20 v/v); R_f = 0.5; white solid; ^{1}H NMR (400 MHz, $CDCl_3$): δ 8.03–7.95 (m, 2H), 7.82 (d, J = 8.0 Hz, 2H), 7.68 (s, 1H), 7.65–7.56 (m, 4H), 7.54–7.44 (m, 4H), 7.29–7.26 (m, 1H), 7.26–7.18 (m, 2H), 6.81–6.72 (m, 2H), 3.74 (s, 3H); $^{13}C\{^{1}H\}$ NMR (100 MHz, $CDCl_3$): δ 197.22, 159.16, 156.93, 152.55, 139.97, 135.61, 132.02, 130.72, 130.34, 129.85, 129.02, 128.87, 128.66, 128.48, 128.02, 127.41, 125.45, 125.41, 123.14, 122.84, 122.49, 122.41, 113.95, 111.97, 77.04, 55.26; HRMS (m/z): $[M +H]^+$ calcd. for $C_{33}H_{22}F_3O_2$, 523.1516; found, 523.1524.

6,12-Diphenyl-12-(*p*-tolyl)-12*H*-fluoreno[2,1-*b*]benzofuran (Figure 5, compound 4aa)

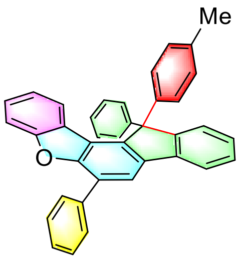

mp: 98–99 °C; TLC (ethyl acetate:petroleum ether, 1:20 v/v); R_f = 0.7; white solid; ^{1}H NMR (400 MHz, $CDCl_3$): δ 8.04 (s, 1H), 7.98 (d, J = 7.6 Hz, 2H), 7.82 (d, J = 7.6 Hz, 1H), 7.59 (t, J = 7.6 Hz, 2H), 7.54–7.49 (m, 2H), 7.48–7.44 (m, 3H), 7.40–7.36 (m, 3H), 7.33 (d, J = 7.6 Hz, 1H), 7.28–7.18 (m, 6H), 7.03 (d, J = 8.0 Hz, 2H), 2.27 (s, 3H); ^{13}C{^{1}H} NMR (100 MHz, $CDCl_3$): δ 156.67, 153.89, 153.69, 146.11, 141.77, 139.79, 138.45, 136.78, 136.55, 136.35, 129.13, 129.02, 128.91, 128.85, 128.80, 128.71, 128.30, 127.88, 127.52, 127.20, 127.03, 126.91, 126.20, 125.65, 125.13, 123.27, 122.22, 119.44, 119.08, 111.44, 65.52, 20.97; HRMS (m/z): $[M+H]^+$ calcd. for $C_{38}H_{27}O$, 499.2056; found, 499.2060.

6-(4-Methoxyphenyl)-12-phenyl-12-(*p*-tolyl)-12*H*-fluoreno[2,1-*b*]benzofuran (Figure 5, compound 4ca)

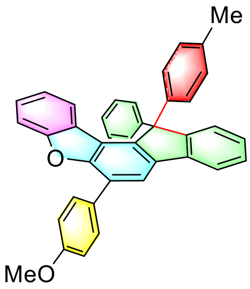

mp: 207–208 °C; TLC (ethyl acetate:petroleum ether, 1:20 v/v); R_f = 0.7; white solid ; ^{1}H NMR (400 MHz, $CDCl_3$): δ 7.99 (s, 1H), 7.94–7.89 (m, 2H), 7.81 (d, J = 7.6 Hz, 1H), 7.51 (d, J = 8.4 Hz, 1H), 7.47–7.42 (m, 3H), 7.38–7.34 (m, 3H), 7.33–7.30 (m, 1H), 7.25–7.23 (m, 1H), 7.22–7.17 (m, 4H), 7.12 (d, J = 8.8 Hz, 2H), 7.03 – 6.98 (m, 3H), 3.93 (s, 3H), 2.26 (s, 3H); ^{13}C{^{1}H} NMR (100 MHz, $CDCl_3$): δ 159.42, 156.63, 153.91, 145.52, 141.81, 139.85, 138.49, 136.50, 136.30, 130.22, 129.15, 128.98, 128.84, 128.79, 128.27, 127.47, 127.13, 126.93, 126.86, 125.62, 125.32, 125.11, 123.32, 122.47, 122.16, 119.39, 118.66, 114.17, 111.39, 55.42, 20.96; HRMS (m/z): $[M+H]^+$ calcd. for

$C_{39}H_{29}O_2$, 529.2162; found, 529.2159.

2-Chloro-6-(4-methoxyphenyl)-12-phenyl-12-(*p*-tolyl)-12*H*-fluoreno[2,1-*b*]benzofuran (Figure 5, compound 4ia)

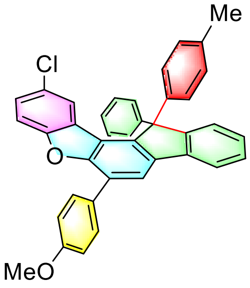

mp: 185–186 °C; TLC (ethyl acetate:petroleum ether, 1:20 v/v); R_f = 0.7; white solid; ^{1}H NMR (400 MHz, $CDCl_3$): δ 8.00 (s, 1H), 7.91–7.86 (m, 2H), 7.80 (d, J = 7.6 Hz, 1H), 7.46–7.40 (m, 4H), 7.37 (d, J = 7.6 Hz, 1H), 7.34–7.27 (m, 3H), 7.26–7.21 (m, 4H), 7.14–7.10 (m, 2H), 7.08–7.05 (m, 2H), 7.03 (s, 1H), 3.92 (s, 3H), 2.28 (s, 3H); $^{13}C\{^1H\}$ NMR (100 MHz, $CDCl_3$): δ 159.53, 154.94, 154.17, 153.87, 145.94, 141.57, 139.63, 138.26, 136.75, 136.63, 130.19, 129.13, 128.78, 128.72, 128.43, 127.60, 127.56, 127.25, 127.08, 127.02, 125.70, 125.56, 124.78, 124.63, 121.70, 119.51, 119.32, 114.21, 112.31, 65.47, 55.43, 20.95; HRMS (m/z): $[M+H]^+$ calcd. for $C_{39}H_{28}ClO_2$, 563.1772; found, 563.1766.

6,12-Diphenyl-12*H*-fluoreno[2,1-*b*]benzofuran-12-ol (Figure 5, compound 5aa)

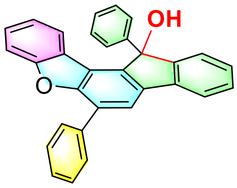

mp 210-211 °C; TLC (ethyl acetate:petroleum ether, 1:20 v/v); R_f = 0.4; white solid; ^{1}H NMR (400 MHz, $CDCl_3$): δ 8.01–7.95 (m, 2H), 7.92 (s, 1H), 7.72–7.66 (m, 2H), 7.63–7.56 (m, 4H), 7.53–7.47 (m, 2H), 7.41–7.33 (m, 3H), 7.32–7.26 (m, 2H), 7.25–7.19 (m, 2H), 7.15–7.10 (m, 1H), 2.62 (s, 1H); $^{13}C\{^1H\}$ NMR (100 MHz, $CDCl_3$): δ 156.88, 154.19, 150.92, 142.61, 141.76, 139.08, 136.53, 135.86, 129.13, 129.02, 128.78, 128.52, 128.08, 127.87, 127.56, 127.44, 126.78, 125.25, 124.69, 124.49, 122.97, 122.62, 119.76, 118.37, 111.44, 83.99; HRMS (m/z): $[M+H]^+$ calcd. for $C_{31}H_{21}O_2$, 425.1536; found, 425.1542.

9-Methoxy-12-(3-methoxyphenyl)-6-phenyl-12*H*-fluoreno[2,1-*b*]benzofuran-12-ol (Figure 5, compound 5af)

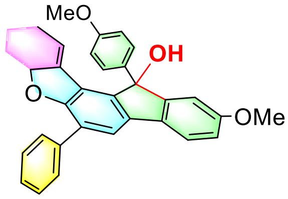

mp: 99–100 °C; TLC (ethyl acetate:petroleum ether, 1:20 v/v); R_f = 0.3; white solid; ^{1}H NMR (400 MHz, $CDCl_3$): δ 8.00–7.94 (m, 2H), 7.85–7.81 (s, 1H), 7.73 (d, J = 7.6 Hz, 1H), 7.61–7.56 (m, 2H), 7.52–7.46 (m, 2H), 7.38–7.33 (m, 1H), 7.30–7.26 (m, 1H), 7.22 – 7.12 (m, 4H), 7.07–7.03 (m, 1H), 6.76–6.68 (m, 2H), 3.82 (s, 3H), 3.75 (s, 3H), 2.63 (s, 1H); $^{13}C\{^1H\}$ NMR (100 MHz, $CDCl_3$): δ 160.84, 159.71, 156.85, 154.22, 143.91, 143.45, 143.14, 140.62, 136.50, 135.47, 129.52, 129.01, 128.77, 128.05, 127.52, 126.58, 125.15, 124.81, 122.99, 122.66, 122.12, 118.25, 117.64, 113.10, 112.19, 111.47, 111.40, 105.45, 83.38, 55.55, 55.22; HRMS (m/z): $[M-OH+H]^+$ calcd. for $C_{33}H_{23}O_3$, 467.1642; found, 467.1634.

2-Phenyl-6-(*p*-tolyl)-12*H*-fluoreno[2,1-*b*]benzofuran-12-ol (Figure 5, compound 5ba)

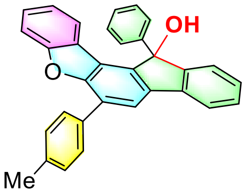

mp: 222-223 °C; TLC (ethyl acetate:petroleum ether, 1:20 v/v); R_f =0.3; white solid; ^{1}H NMR (400 MHz, $CDCl_3$): δ 7.92–7.85 (m, 3H), 7.69 (dd, J = 12.0, 7.6 Hz, 2H), 7.58 (d, J = 8.0 Hz, 2H), 7.51 (d, J = 8.4 Hz, 1H), 7.43 – 7.38 (m, 3H), 7.37 – 7.33 (m, 2H), 7.32–7.26 (m, 2H), 7.24–7.19 (m, 2H), 7.12 (t, J = 7.6 Hz, 1H), 2.61 (s, 1H), 2.50 (s, 3H); $^{13}C\{^1H\}$ NMR (100 MHz, $CDCl_3$): δ 156.86, 154.20, 150.94, 142.30, 141.81, 139.14, 137.97, 135.81, 133.59, 129.51, 129.10, 128.87, 128.51, 127.81, 127.50, 127.41, 126.80, 125.26, 124.66, 124.46, 122.92, 122.66, 122.13, 119.74, 118.18, 111.44, 83.98, 77.37, 77.05, 76.73, 21.37; HRMS (m/z): $[M+CH_3OH]^+$ calcd. for $C_{33}H_{26}O_3$, 470.1876; found, 470.1869.

4-Bromo-12-phenyl-6-(*p*-tolyl)-12*H*-fluoreno[2,1-*b*]benzofuran-12-ol (Figure 5, compound 5oa)

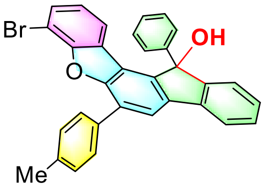

mp: 112–113 °C; TLC (ethyl acetate:petroleum ether, 1:20 v/v); R_f = 0.4; white solid; ^{1}H NMR (400 MHz, $CDCl_3$): δ 7.98–7.91 (m, 3H), 7.69 (d, *J* = 7.6 Hz, 1H), 7.60 (d, *J* = 7.6 Hz, 1H), 7.53 (d, *J* = 7.2 Hz, 2H), 7.50 (d, *J* = 8.0 Hz, 1H), 7.43–7.32 (m, 4H), 7.28–7.19 (m, 4H), 6.98 (t, *J* = 8.0 Hz, 1H), 2.60 (s, 1H), 2.49 (s, 3H); ^{13}C{^{1}H} NMR (100 MHz, $CDCl_3$): δ 153.96, 153.88, 150.82, 142.58, 141.59, 138.88, 138.12, 136.33, 133.02, 130.33, 129.57, 129.18, 128.74, 128.54, 128.00, 127.51, 126.99, 125.18, 124.46, 124.12, 123.63, 122.23, 119.85, 118.55, 104.13, 83.88, 21.38; HRMS (m/z): $[M+H]^+$ calcd. for $C_{32}H_{22}BrO_2$, 517.0798; found, 517.0807.

10. X-ray crystallographic data of compound 3ha

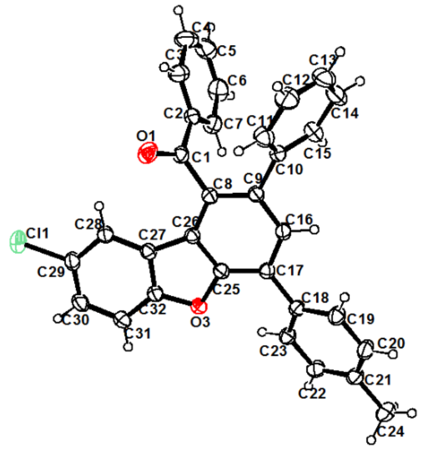

The purified compound **3ha** is dissolved in a mixed solvent of dichloromethane and *n*-hexane, and placed in a dark cabinet to slowly evaporate. After several days, a colourless bulk crystal is obtained. The X-ray crystal-structure determinations were obtained on a Bruker Smart CCDC APEX-2 diffractometer (graphite- monochromated Mo $K\alpha$ radiation, λ=0.71073 nm) at 293(2) K.

Supplementary Figure 1. ORTEP drawing of compound **3ha** (30% probability for the thermal ellipsoid).

Supplementary Table 1. Crystal data and structure refinement for compound **3ha**.

CCDC number	2022149
Identification code	hexw4
Empirical formula	C32 H21 Cl O2
Formula weight	472.94
Temperature	296(2) K
Wavelength	0.71073 Å
Crystal system	Monoclinic
Space group	$P2_1/n$
Unit cell dimensions	a = 21.4091(12) Å α= 90°.
	b = 6.3562(3) Å β= 94.401(2)°.
	c = 17.3108(10) Å γ= 90°.
Volume	2348.7(2) $Å^3$
Z	4
Density (calculated)	1.337 Mg/m^3
Absorption coefficient	0.191 mm^{-1}
F(000)	984
Crystal size	0.400 x 0.300 x 0.300 mm^3
Theta range for data collection	1.908 to 27.926°.
Index ranges	-28<=h<=28, -8<=k<=8, -22<=l<=22
Reflections collected	72884
Independent reflections	5626 [R(int) = 0.0323]
Completeness to theta = 25.242°	99.5%

Refinement method	Full-matrix least-squares on F^2
Data / restraints / parameters	5626 / 0 / 316
Goodness-of-fit on F^2	1.061
Final R indices [I>2sigma(I)]	R1 = 0.0429, wR2 = 0.1194
R indices (all data)	R1 = 0.0551, wR2 = 0.1346
Extinction coefficient	n/a
Largest diff. peak and hole	0.245 and -0.318 e.Å^{-3}

11. X-ray crystallographic data of compound 5aa

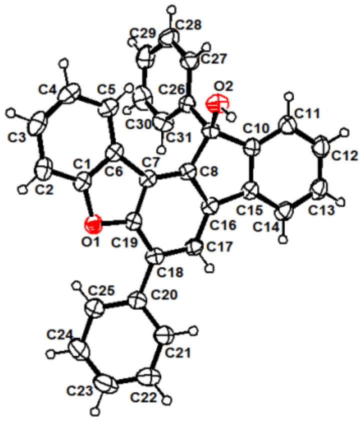

The purified compound **5aa** is dissolved in a mixed solvent of ethyl acetate and petroleum ether, and placed in a dark cabinet to slowly evaporate. After several days, a colourless bulk crystal is obtained. The X-ray crystal-structure determinations were obtained on a Bruker Smart CCDC APEX-2 diffractometer (graphite-monochromated Mo *Kα* radiation, λ=0.71073 nm) at 273(2) K.

Supplementary Figure 2. ORTEP drawing of compound **5aa** (30% probability for the thermal ellipsoid).

Supplementary Table 2. Crystal data and structure refinement for compound **5aa**.

CCDC number	2022150	
Identification code	1	
Empirical formula	C31 H20 O2	
Formula weight	424.47	
Temperature	273(2) K	
Wavelength	0.71073 Å	
Crystal system	Triclinic	
Space group	P-1	
Unit cell dimensions	a = 11.4097(12) Å	α= 85.916(5)°.
	b = 11.9749(14) Å	β= 79.904(5)°.
	c = 17.294(2) Å	γ= 69.798(4)°.
Volume	2183.0(4) $Å^3$	
Z	4	
Density (calculated)	1.292 Mg/m^3	
Absorption coefficient	0.080 mm^{-1}	
F(000)	888	
Crystal size	0.230 x 0.210 x 0.210 mm^3	
Theta range for data collection	2.821 to 27.859°.	
Index ranges	-14<=h<=14, -15<=k<=8,15 -22<=l<=22	
Reflections collected	106424	
Independent reflections	10255 [R(int) = 0.0429]	
Completeness to theta = 25.242°	99.8%	

Refinement method	Full-matrix least-squares on F^2
Data / restraints / parameters	10255 / 0 / 597
Goodness-of-fit on F^2	1.029
Final R indices [I>2sigma(I)]	R1 = 0.0447, wR2 = 0.1011
R indices (all data)	R1 = 0.0674, wR2 = 0.1138
Extinction coefficient	n/a
Largest diff. peak and hole	0.189 and -0.221 e.Å^{-3}

12. ^{1}H and ^{13}C NMR spectra for all compounds

(2,4-Diphenyldibenzo[*b,d*]furan-1-yl)(phenyl)methanone (Figure 2, compound 3aa)

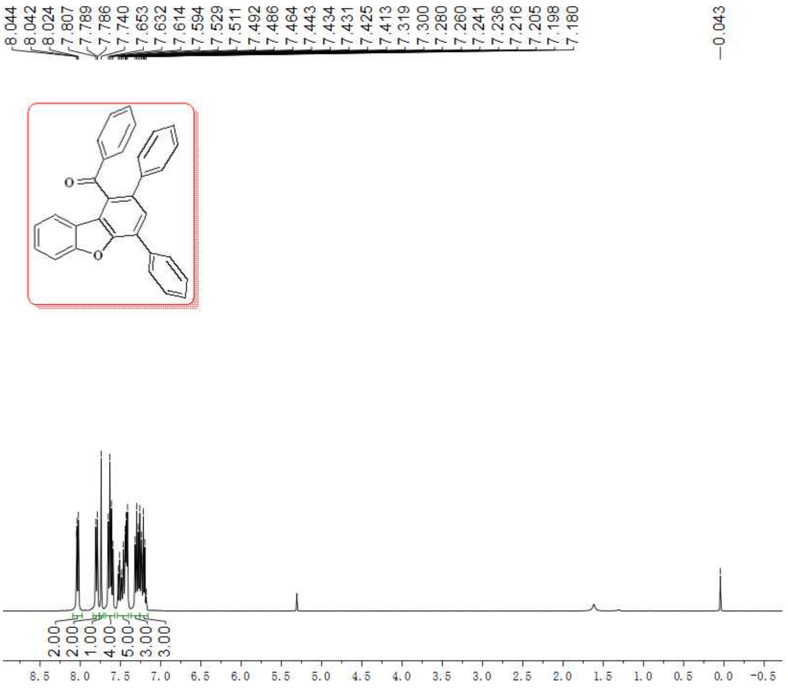

Parameter	值
1 Data File Name	F:/ 实验数据/ 实验数据/ 97/ pdata/ 1/ 1r
2 标题	2020-1-hxw-H.97.1.1r
3 Comment	
4 Origin	Bruker BioSpin GmbH
5 Owner	nmrsu
6 Site	
7 Instrument	Avance
8 Author	
9 Solvent	CDCl3
10 Temperature	296.6
11 Pulse Sequence	zg30
12 Experiment	1D
13 Probe	Z163739_0032 (PI HR-400-S1-BBF/ H/ D-5.0-Z SP)
14 Number of Scans	4
15 Receiver Gain	99.2
16 Relaxation Delay	1.0000
17 Pulse Width	10.0000
18 Presaturation Frequency	
19 Acquisition Time	3.9977
20 Acquisition Date	2020-07-04T14:27:10
21 Modification Date	2020-07-04T14:24:18
22 Class	
23 Spectrometer Frequency	400.13
24 Spectral Width	8196.7
25 Lowest Frequency	-1631.2
26 Nucleus	1H
27 Acquired Size	32768
28 Spectral Size	65536

Supplementary Figure 3 ^{1}H NMR Spectra of compound 3aa

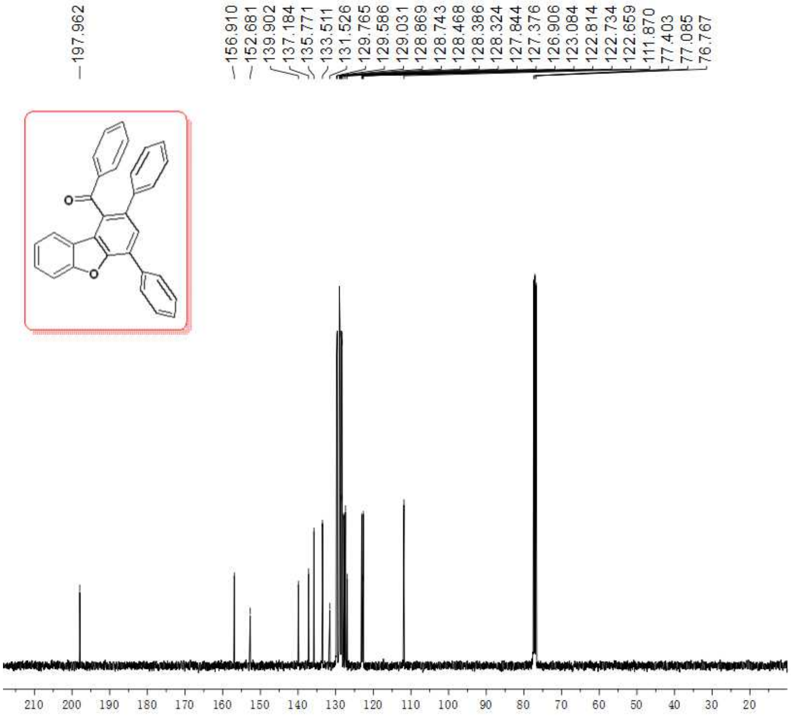

Parameters	
Parameter	值
1 Data File Name	F:/ 实验数据/ 实验数据/ 56/ pdata/ 1/ 1r
2 标题	2020-1-hxw-C.56.1.1r
3 Comment	
4 Origin	Bruker BioSpin GmbH
5 Owner	nmrsu
6 Site	
7 Instrument	Avance
8 Author	
9 Solvent	CDCl3
10 Temperature	297.4
11 Pulse Sequence	zgpg30
12 Experiment	1D
13 Probe	Z163739_0032 (PI HR-400-S1-BBF/ H/ D-5.0-Z SP)
14 Number of Scans	200
15 Receiver Gain	15.3
16 Relaxation Delay	2.0000
17 Pulse Width	10.0000
18 Presaturation Frequency	
19 Acquisition Time	1.3763
20 Acquisition Date	2020-07-05T22:25:48
21 Modification Date	2020-07-05T22:25:50
22 Class	
23 Spectrometer Frequency	100.61
24 Spectral Width	23809.5
25 Lowest Frequency	-1843.5
26 Nucleus	13C
27 Acquired Size	32768
28 Spectral Size	32768

Supplementary Figure 4 ^{13}C NMR Spectra of compound 3aa

Phenyl(2-phenyl-4-(*p*-tolyl)dibenzo[*b,d*]furan-1-yl)methanone (Figure 2, compound 3ba)

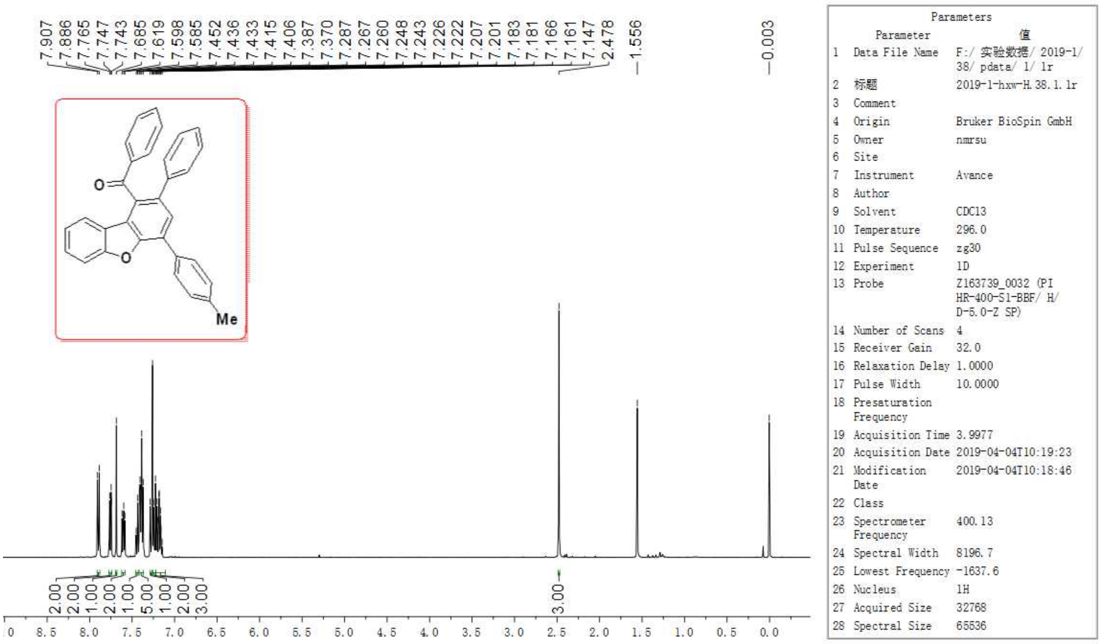

**Supplementary Figure 5 **

Supplementary Figure 5 ^{1}H NMR Spectra of compound 3ba

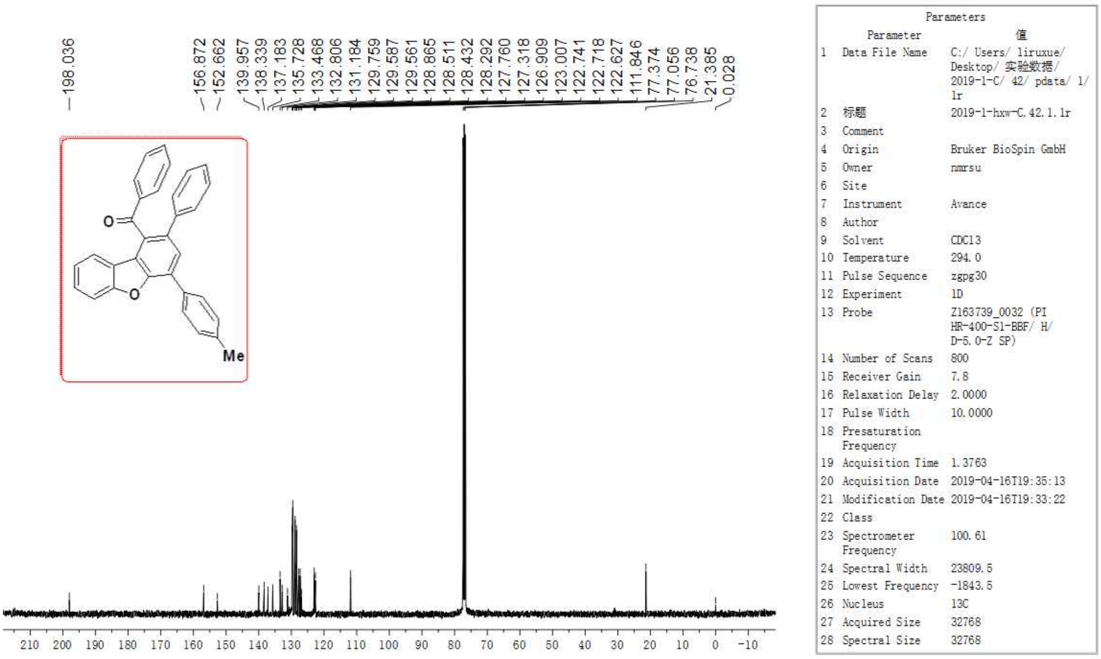

Supplementary Figure 6 ^{13}C NMR Spectra of compound 3ba

(4-(4-Methoxyphenyl)-2-phenyldibenzo[*b,d*]furan-1-yl)(phenyl)methanone (Figure 2, compound 3ca)

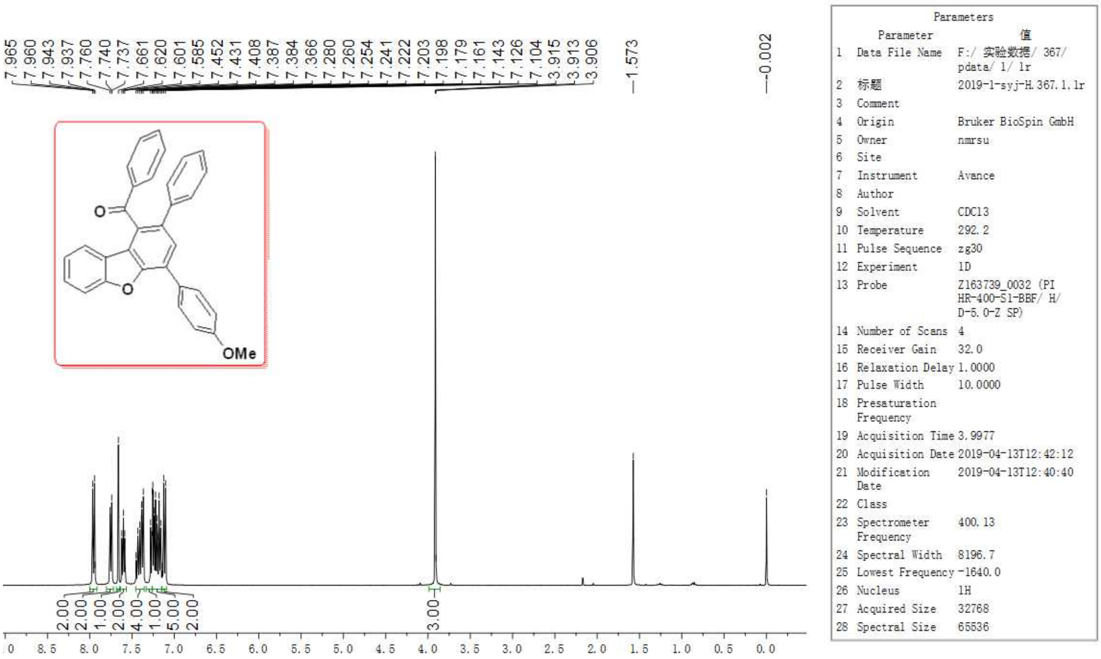

Supplementary Figure 7 ^{1}H NMR Spectra of compound 3ca

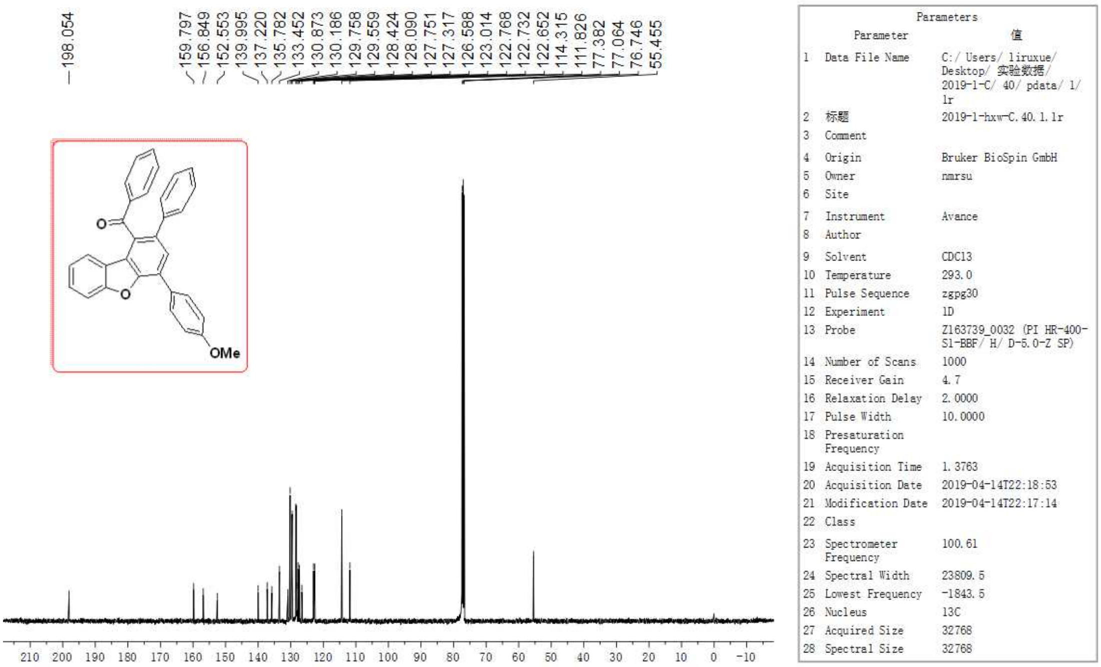

Supplementary Figure 8 ^{13}C NMR Spectra of compound 3ca

(8-Methyl-2,4-diphenyldibenzo[*b,d*]furan-1-yl)(phenyl)methanone (Figure 2, compound 3da)

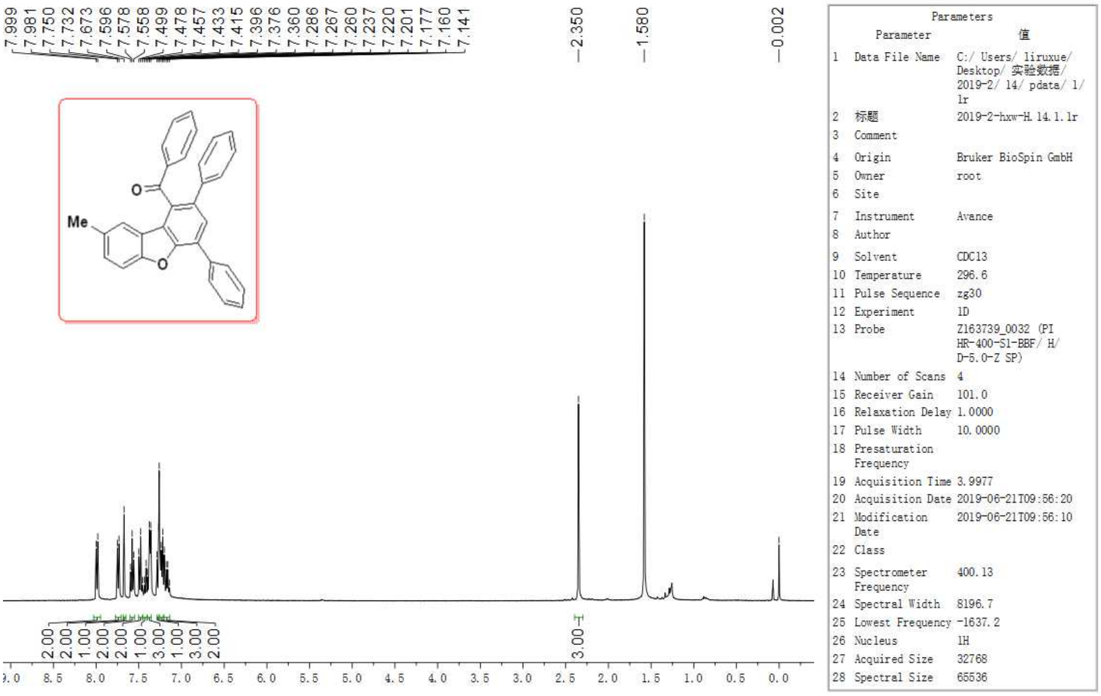

Supplementary Figure 9 ^{1}H NMR Spectra of compound 3da

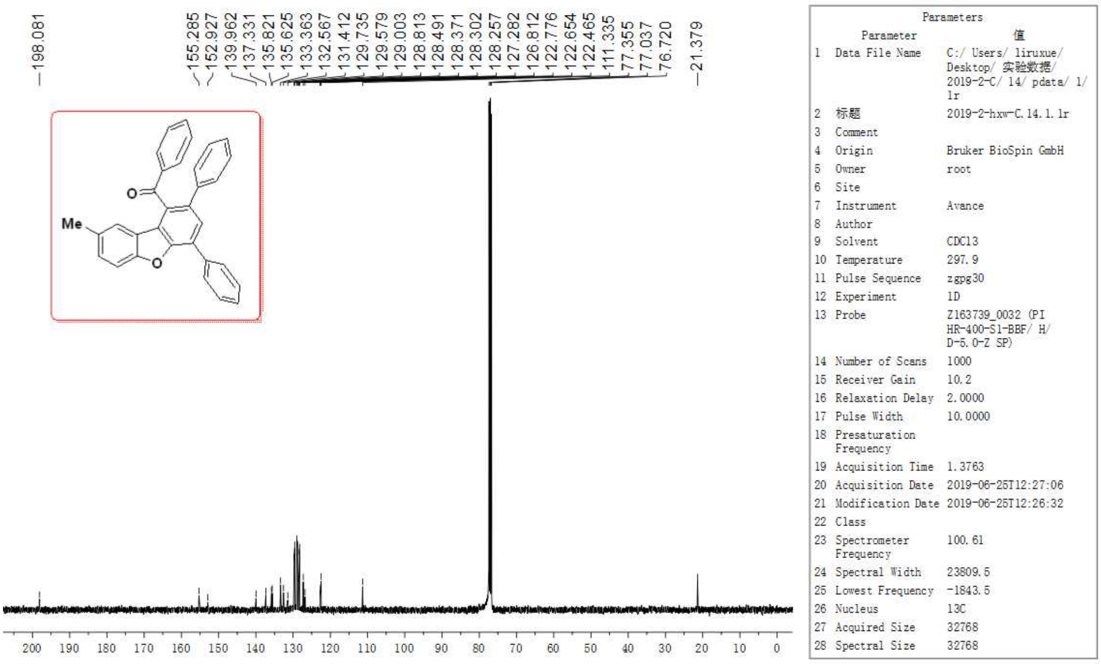

Supplementary Figure 10 ^{13}C NMR Spectra of compound 3da

(8-Methyl-2-phenyl-4-(p-tolyl)dibenzo[*b,d*]furan-1-yl)(phenyl)methanone (Figure 2, compound 3ea)

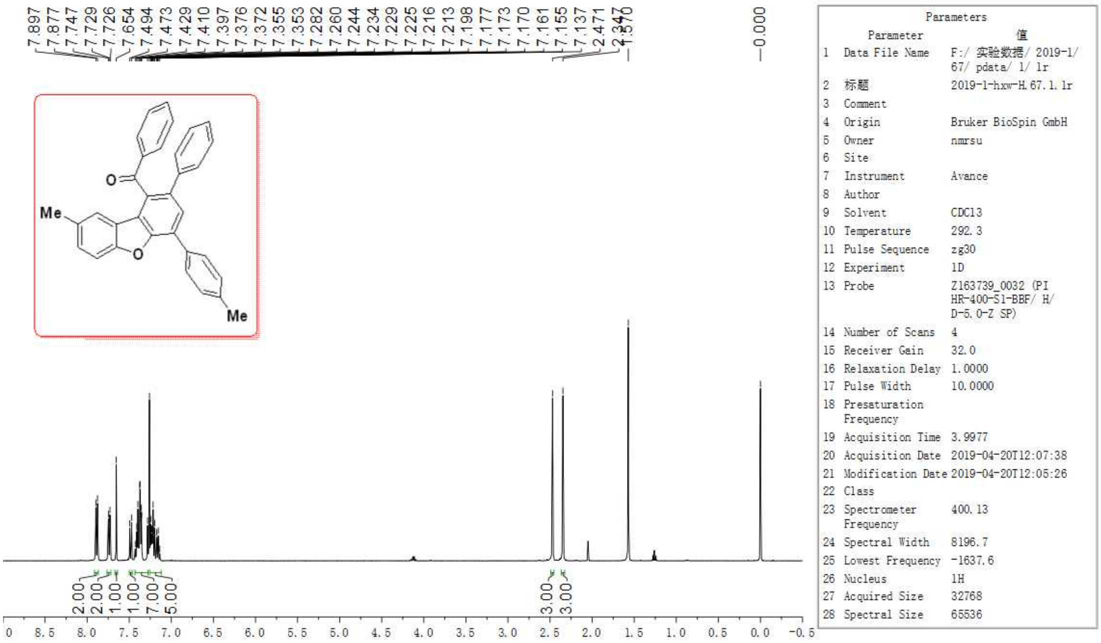

Supplementary Figure 11 ^{1}H NMR Spectra of compound 3ea

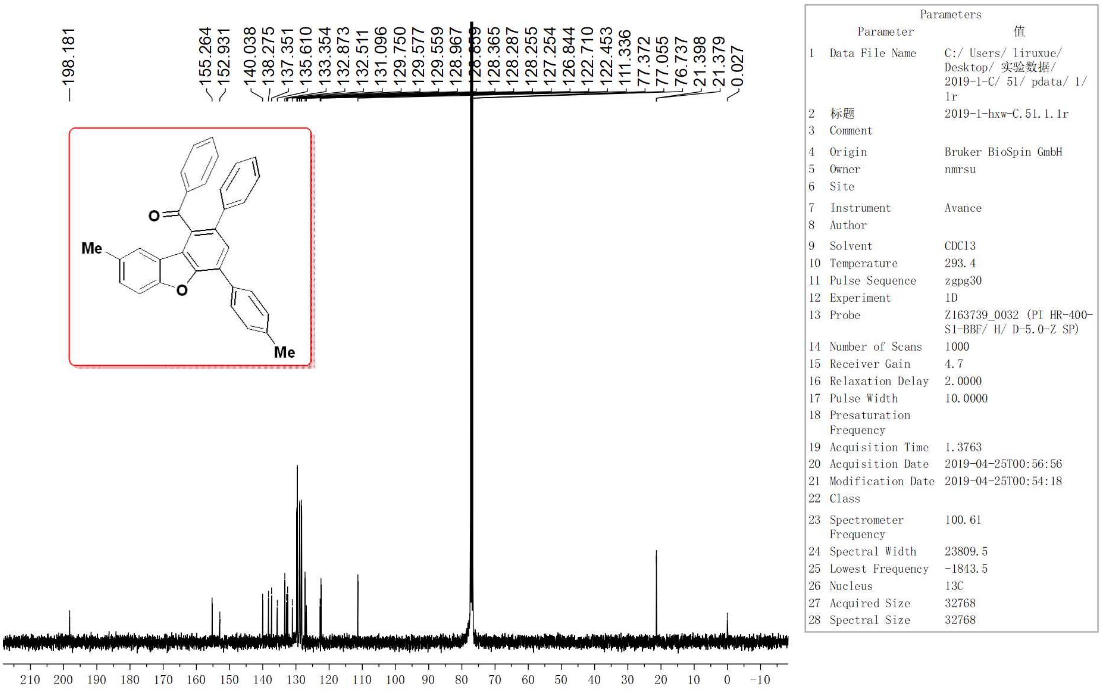

Supplementary Figure 12 ^{13}C NMR Spectra of compound 3ea

(4-(4-Methoxyphenyl)-8-methyl-2-phenyldibenzo[*b,d*]furan-1-yl)(phenyl)methanone (Figure 2, compound 3fa)

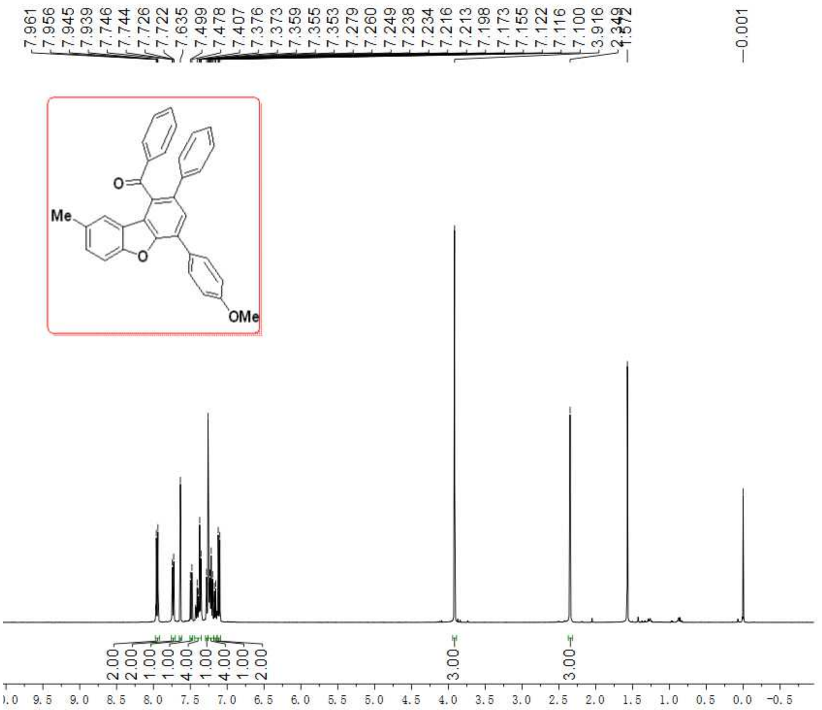

Parameters

	Parameter	值
1	Data File Name	F:/ 实验数据/ 2019-1/ 52/ pdata/ 1/ 1r
2	标题	2019-1-hxw-H.52.1.1r
3	Comment	
4	Origin	Bruker BioSpin GmbH
5	Owner	nmrsu
6	Site	
7	Instrument	Avance
8	Author	
9	Solvent	CDCl3
10	Temperature	293.5
11	Pulse Sequence	zg30
12	Experiment	1D
13	Probe	Z163739_0032 (PI HR-400-S1-BBF/ H/ D-5.0-Z SP)
14	Number of Scans	4
15	Receiver Gain	32.0
16	Relaxation Delay	1.0000
17	Pulse Width	10.0000
18	Presaturation Frequency	
19	Acquisition Time	3.9977
20	Acquisition Date	2019-04-12T10:54:52
21	Modification Date	2019-04-12T10:53:26
22	Class	
23	Spectrometer Frequency	400.13
24	Spectral Width	8196.7
25	Lowest Frequency	-1637.6
26	Nucleus	1H
27	Acquired Size	32768
28	Spectral Size	65536

Supplementary Figure 13 ^{1}H NMR Spectra of compound 3fa

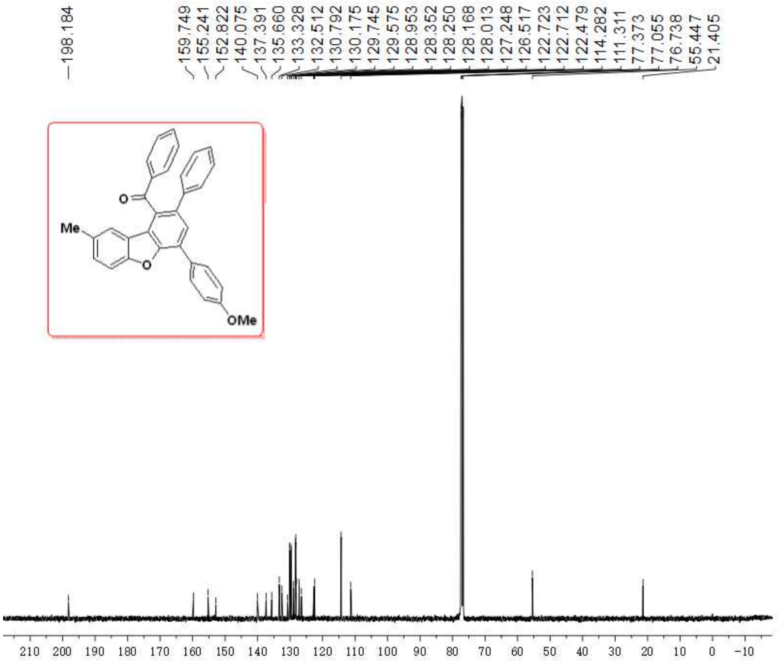

Parameters

	Parameter	值
1	Data File Name	C:/ Users/ linxue/ Desktop/ 实验数据/ 2019-1-C/ 41/ pdata/ 1/ 1r
2	标题	2019-1-hxw-C.41.1.1r
3	Comment	
4	Origin	Bruker BioSpin GmbH
5	Owner	nmrsu
6	Site	
7	Instrument	Avance
8	Author	
9	Solvent	CDCl3
10	Temperature	293.1
11	Pulse Sequence	zgpg30
12	Experiment	1D
13	Probe	Z163739_0032 (PI HR-400-S1-BBF/ H/ D-5.0-Z SP)
14	Number of Scans	1000
15	Receiver Gain	4.7
16	Relaxation Delay	2.0000
17	Pulse Width	10.0000
18	Presaturation Frequency	
19	Acquisition Time	1.3763
20	Acquisition Date	2019-04-14T23:23:41
21	Modification Date	2019-04-14T23:22:00
22	Class	
23	Spectrometer Frequency	100.61
24	Spectral Width	23809.5
25	Lowest Frequency	-1843.5
26	Nucleus	13C
27	Acquired Size	32768
28	Spectral Size	32768

Supplementary Figure 14 ^{13}C NMR Spectra of compound 3fa

(4-(4-Chlorophenyl)-8-methyl-2-phenyldibenzo[*b,d*]furan-1-yl)(phenyl)methanone (Figure 2, compound 3ga)

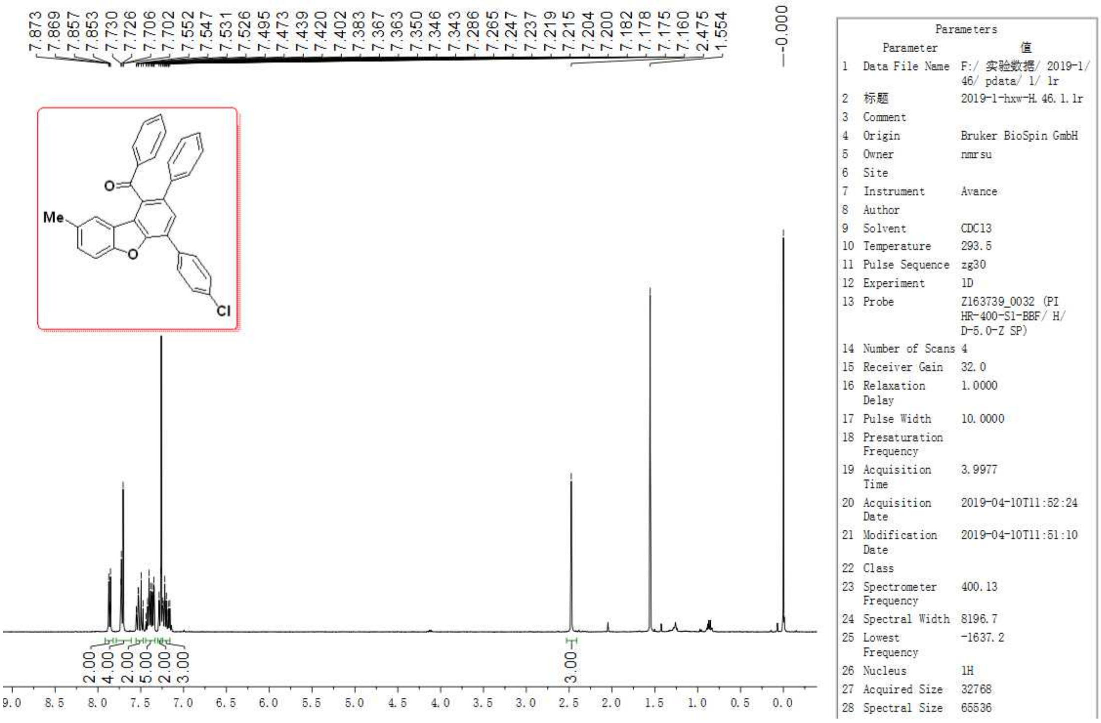

Supplementary Figure 15 ^{1}H NMR Spectra of compound 3ga

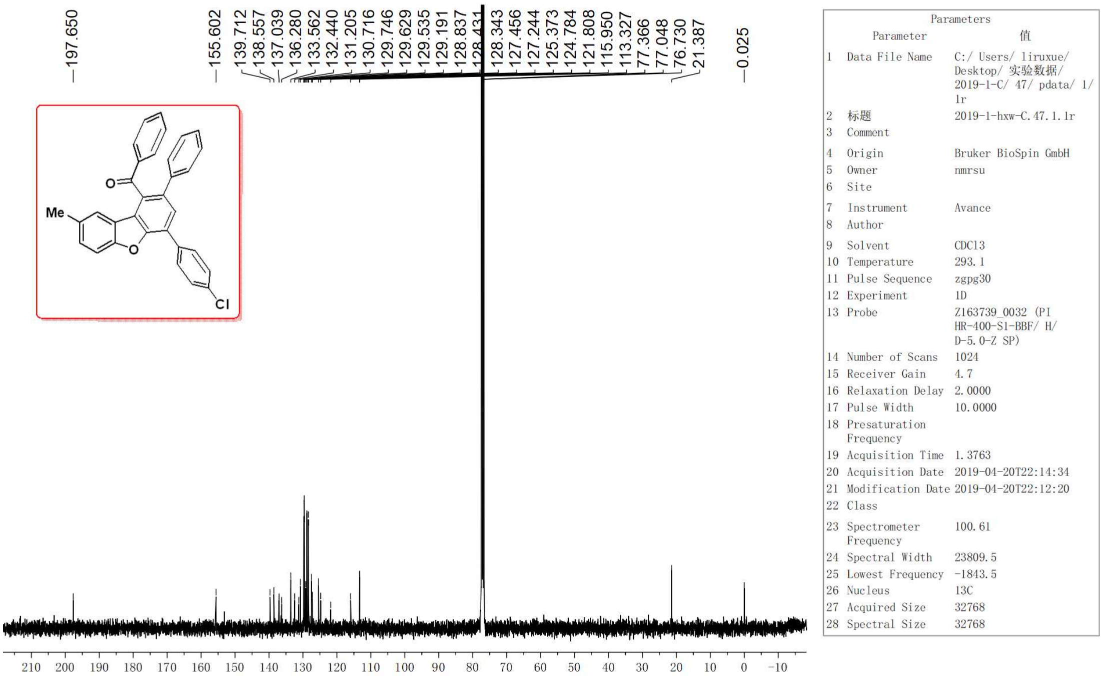

Supplementary Figure 16 ^{13}C NMR Spectra of compound 3ga

(8-Chloro-2-phenyl-4-(*p*-tolyl)dibenzo[*b,d*]furan-1-yl)(phenyl)methanone (Figure 2, compound 3ha)

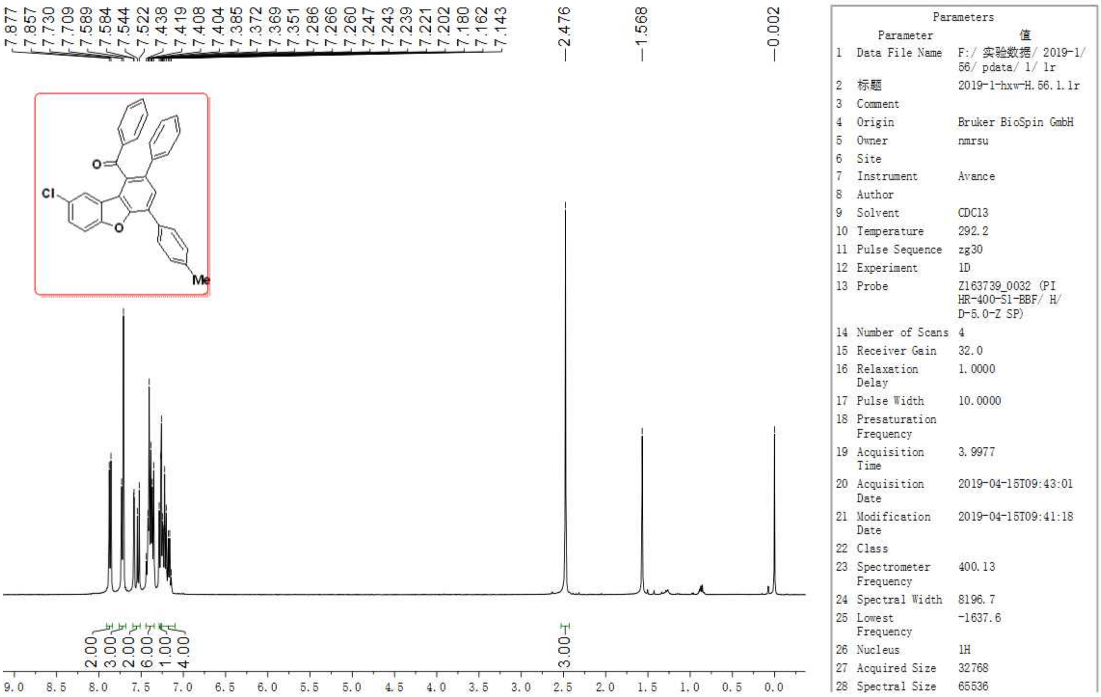

Supplementary Figure 17 ^{1}H NMR Spectra of compound 3ha

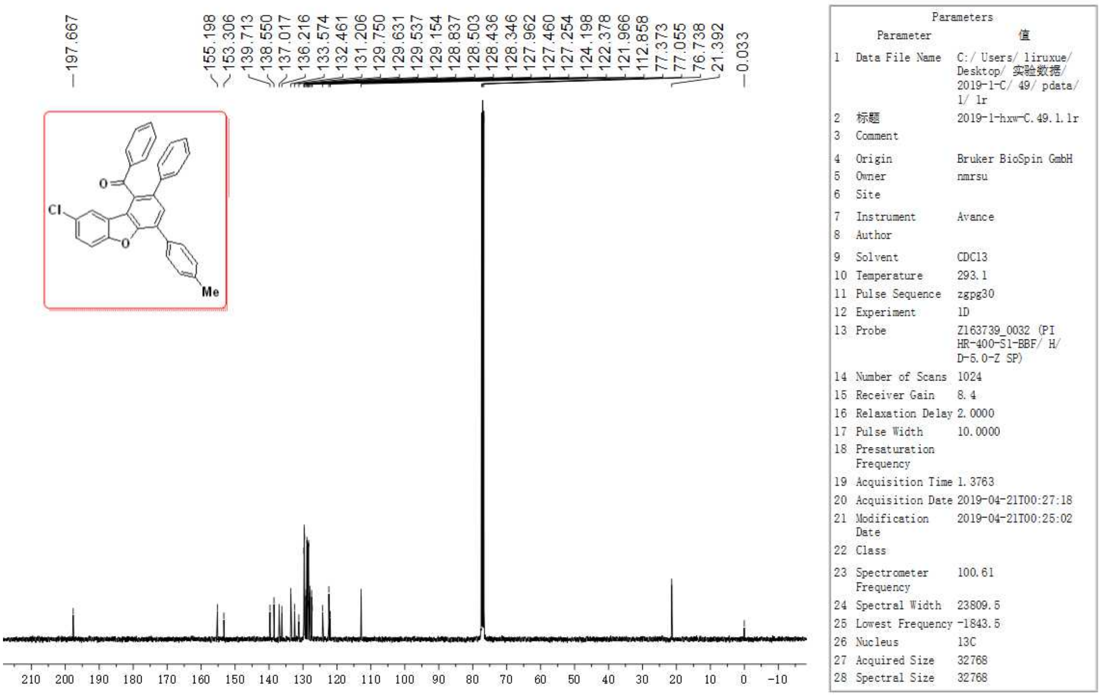

Supplementary Figure 18 ^{13}C NMR Spectra of compound 3ha

(8-Chloro-4-(4-methoxyphenyl)-2-phenyldibenzo[*b,d*]furan-1-yl)(phenyl)methanone (Figure 2, compound 3ia)

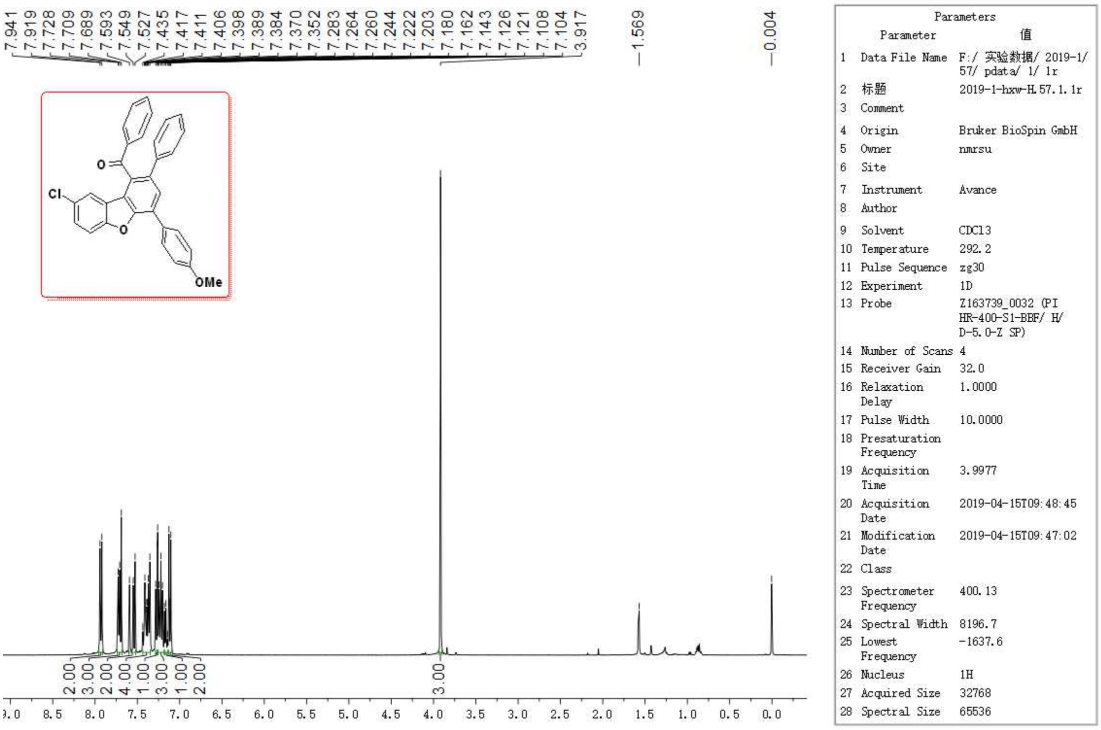

Supplementary Figure 19 ^{1}H NMR Spectra of compound 3ia

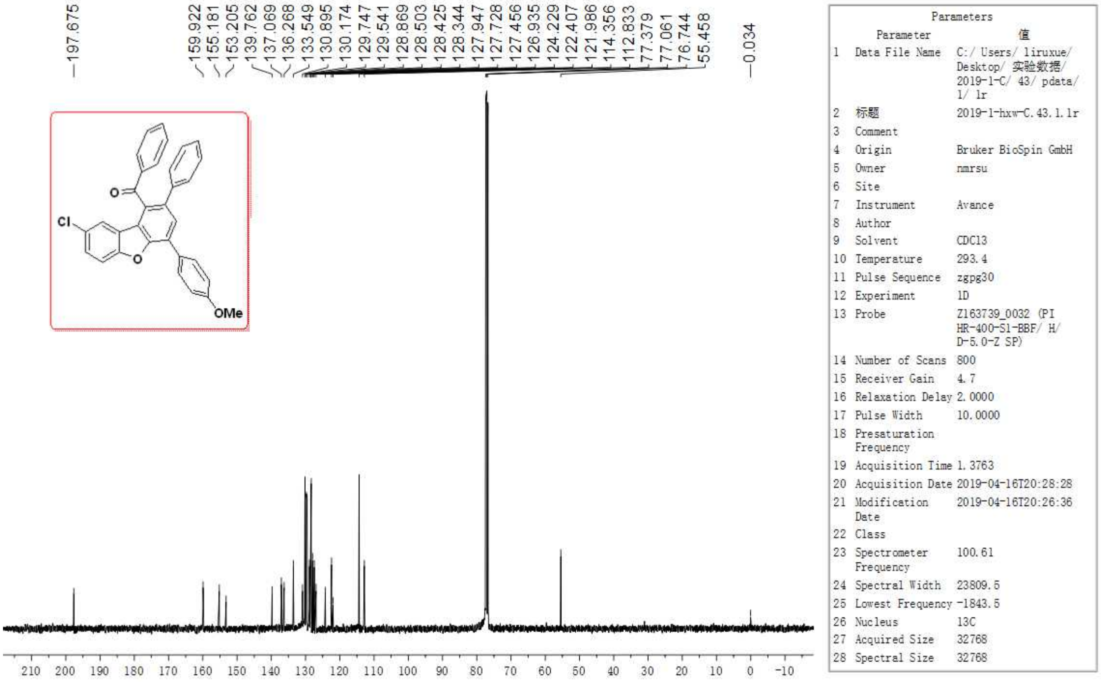

Supplementary Figure 20 ^{13}C NMR Spectra of compound 3ia

(8-Chloro-2-phenyl-4-(*p*-tolyl)dibenzo[*b,d*]furan-1-yl)(phenyl)methanone (Figure 2, compound 3ja)

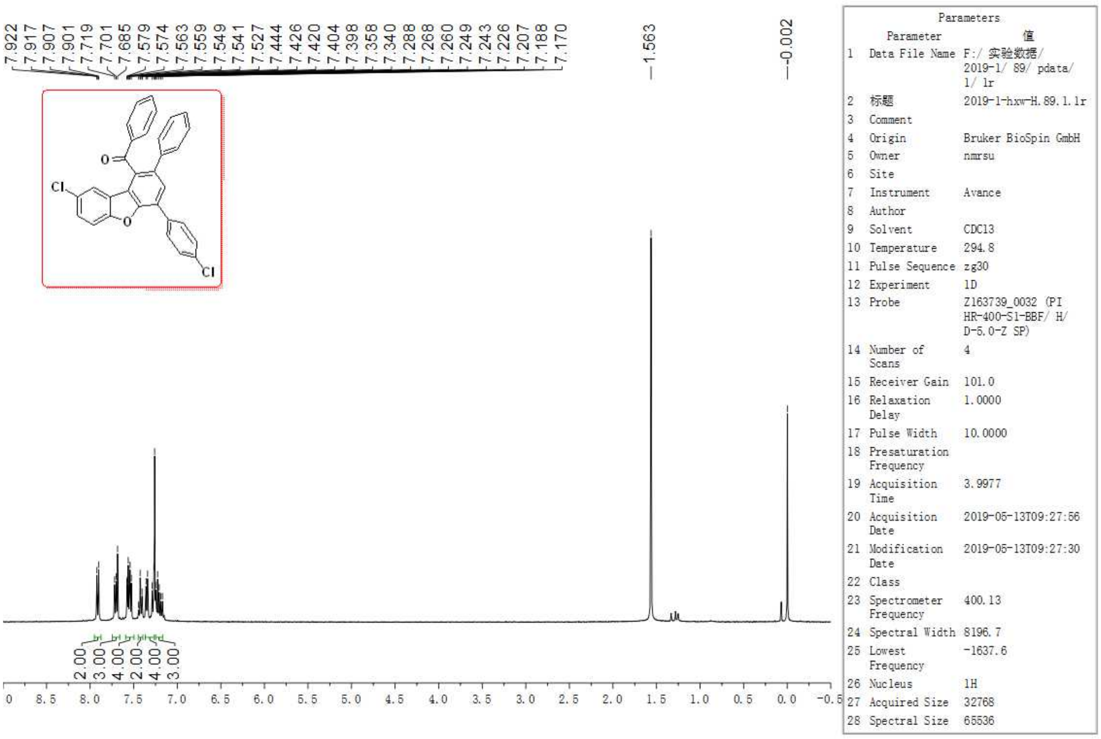

Supplementary Figure 21 ^{1}H NMR Spectra of compound 3ja

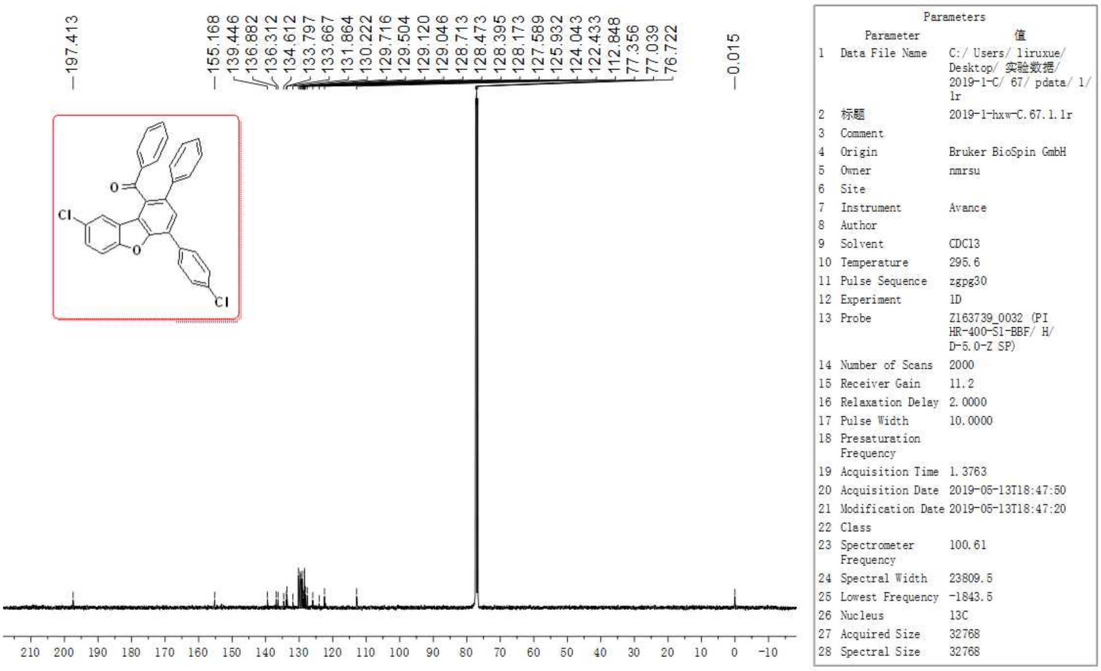

Supplementary Figure 22 ^{13}C NMR Spectra of compound 3ja

(8-Chloro-4-(2-methoxyphenyl)-2-phenyldibenzo[*b,d*]furan-1-yl)(phenyl)methanone (Figure 2, compound 3ka)

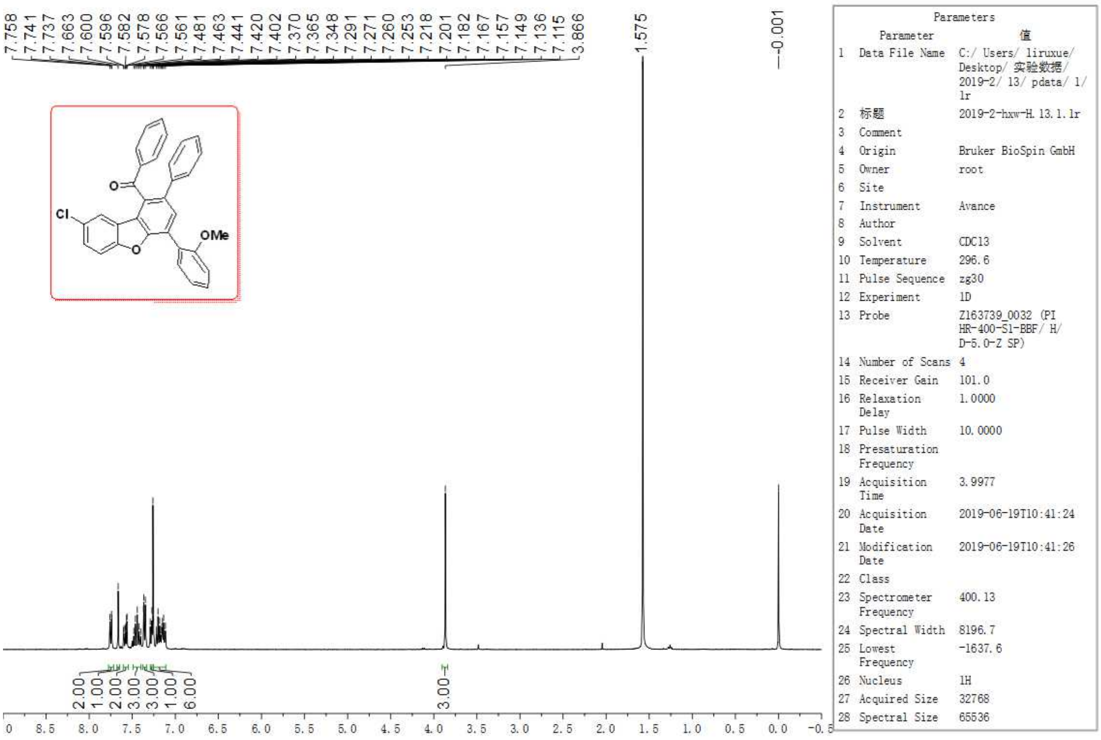

Supplementary Figure 23 ^{1}H NMR Spectra of compound 3ka

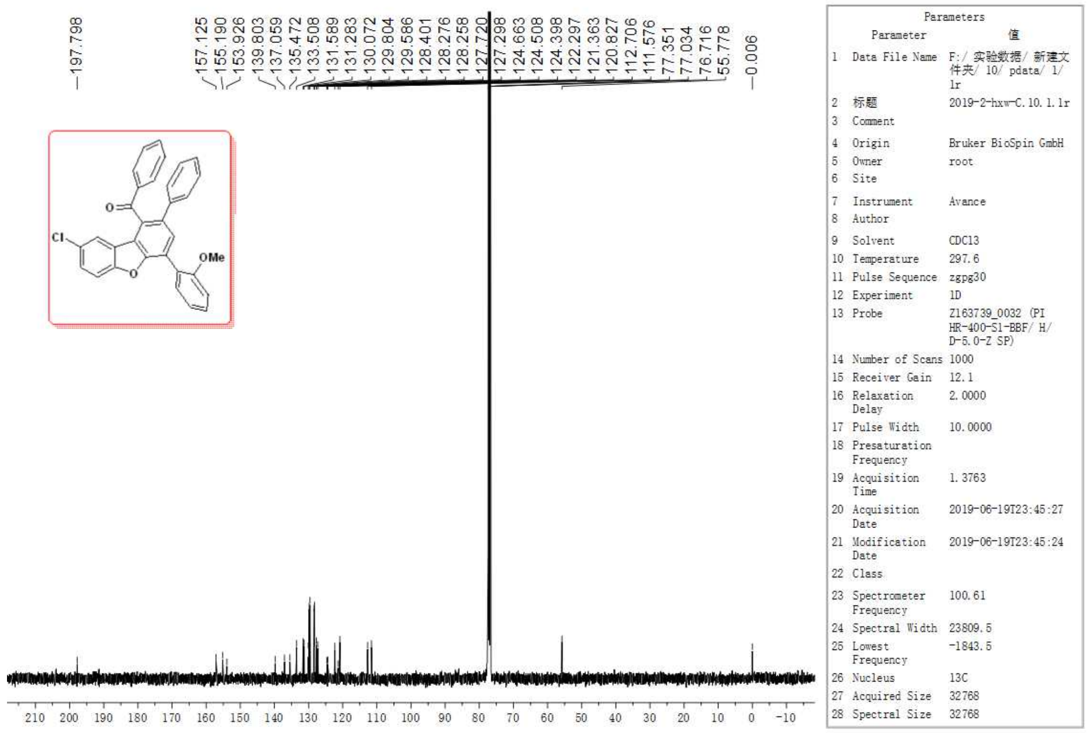

Supplementary Figure 24 ^{13}C NMR Spectra of compound 3ka

(8-Bromo-2,4-diphenyldibenzo[*b,d*]furan-1-yl)(phenyl)methanone (3la)

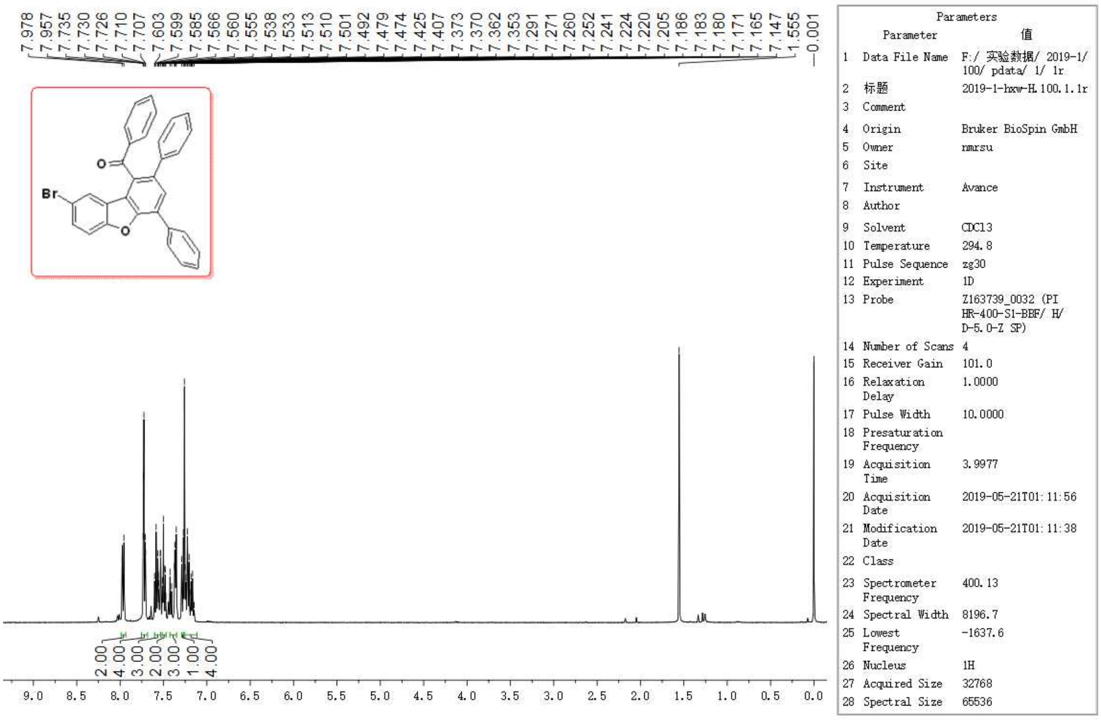

Supplementary Figure 25 ^{1}H **NMR Spectra of compound 3la**

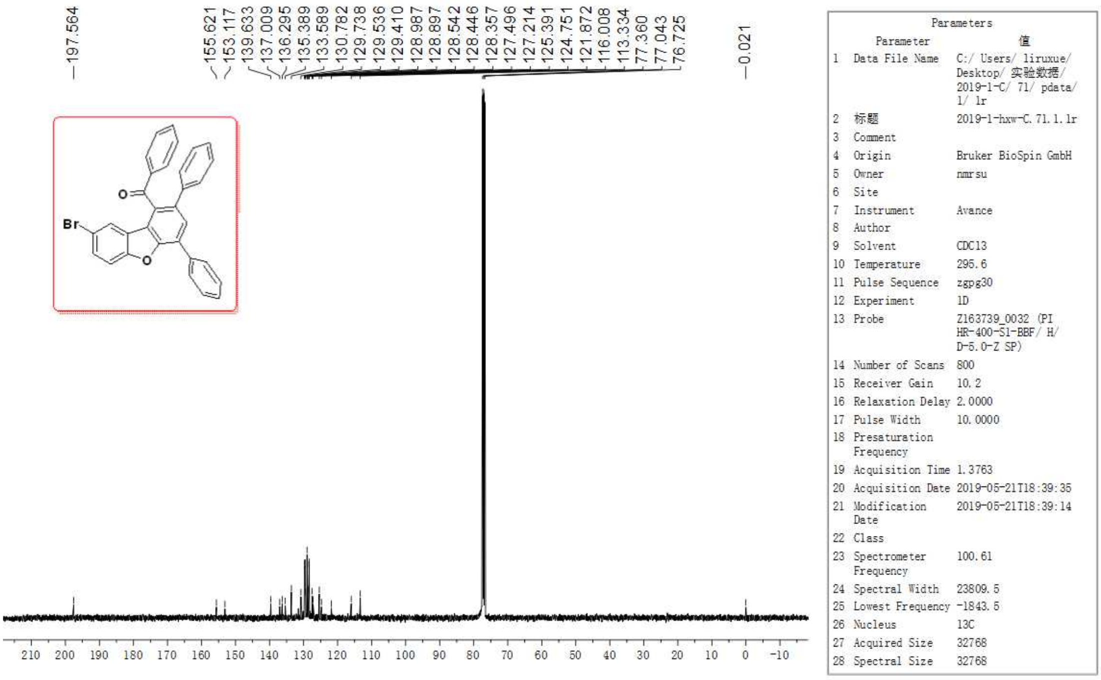

Supplementary Figure 26 ^{13}C **NMR Spectra of compound 3la**

(8-Bromo-2-phenyl-4-(*p*-tolyl)dibenzo[*b,d*]furan-1-yl)(phenyl)methanone (Figure 2, compound 3ma)

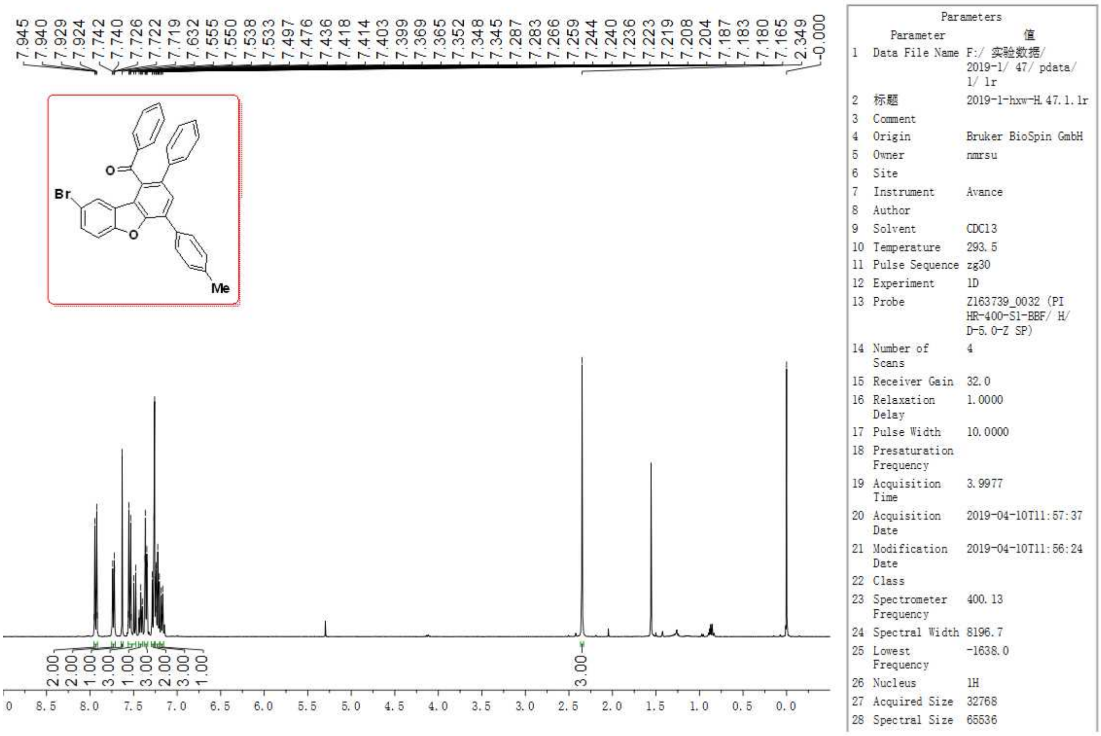

Supplementary Figure 27 ^{1}H NMR Spectra of compound 3ma

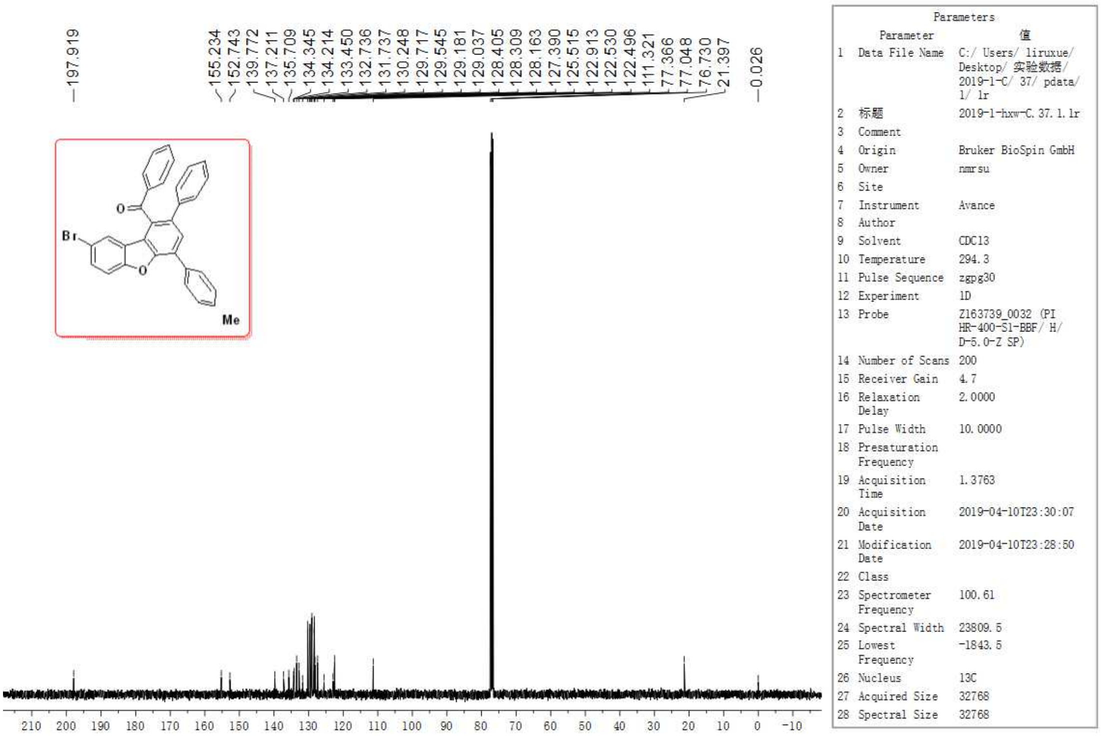

Supplementary Figure 28 ^{13}C NMR Spectra of compound 3ma

(8-Bromo-4-(4-methoxyphenyl)-2-phenyldibenzo[*b,d*]furan-1-yl)(phenyl)methanone (Figure 2, compound 3na)

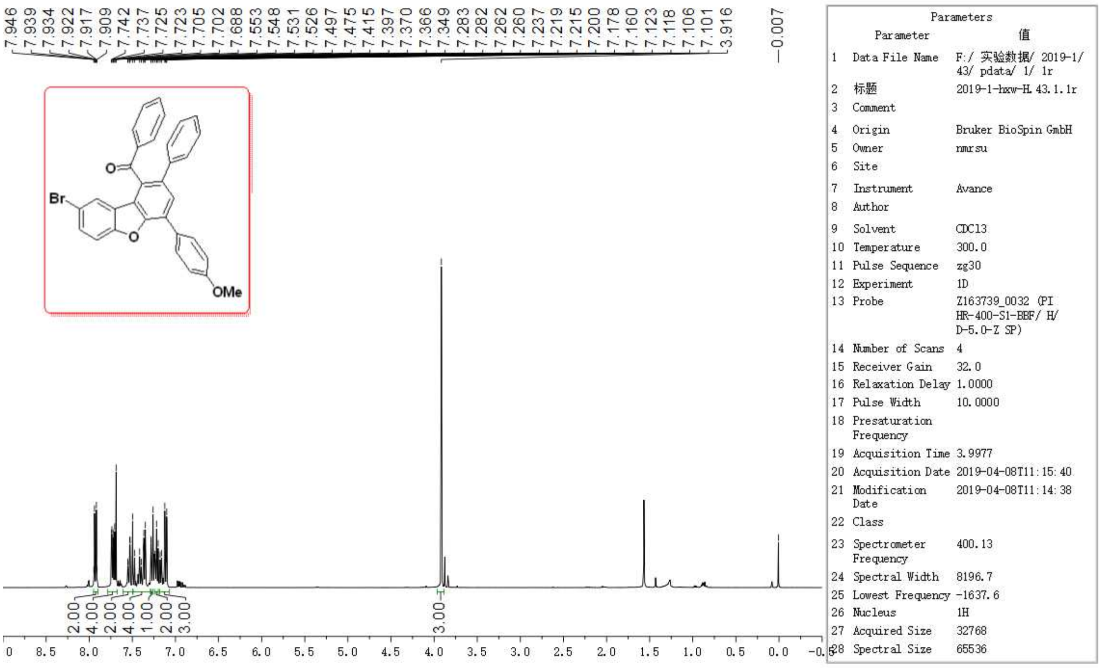

Supplementary Figure 29 ^{1}H NMR Spectra of compound 3na

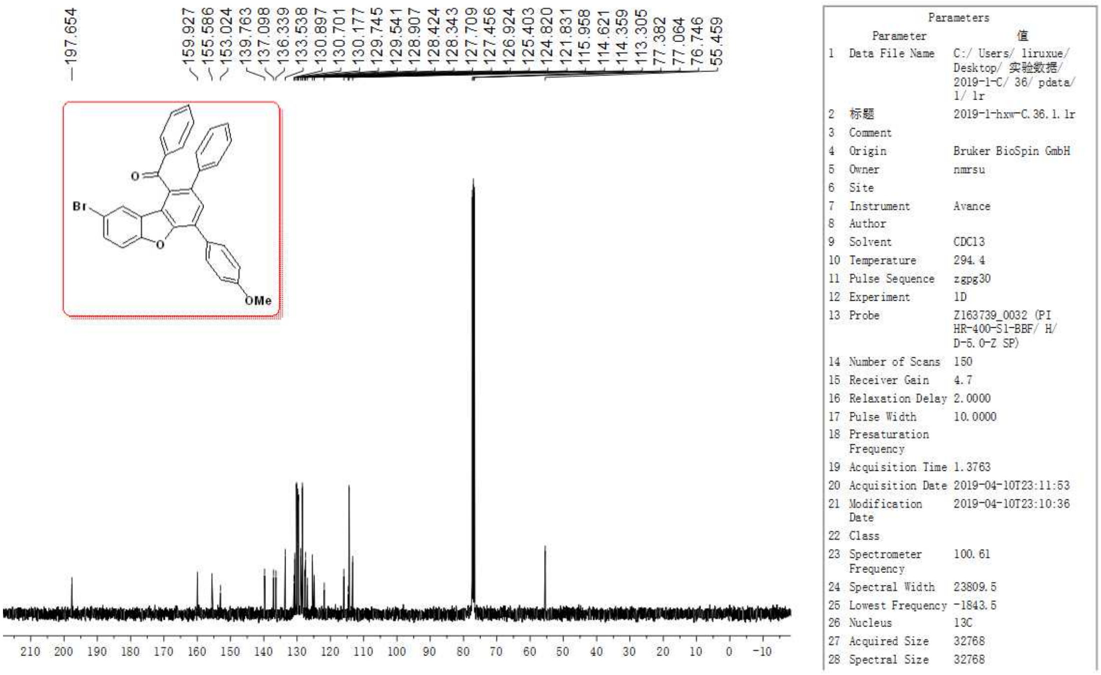

Supplementary Figure 30 ^{13}C NMR Spectra of compound 3na

(6-Bromo-2-phenyl-4-(*p*-tolyl)dibenzo[*b,d*]furan-1-yl)(phenyl)methanone (Figure 2, compound 3oa)

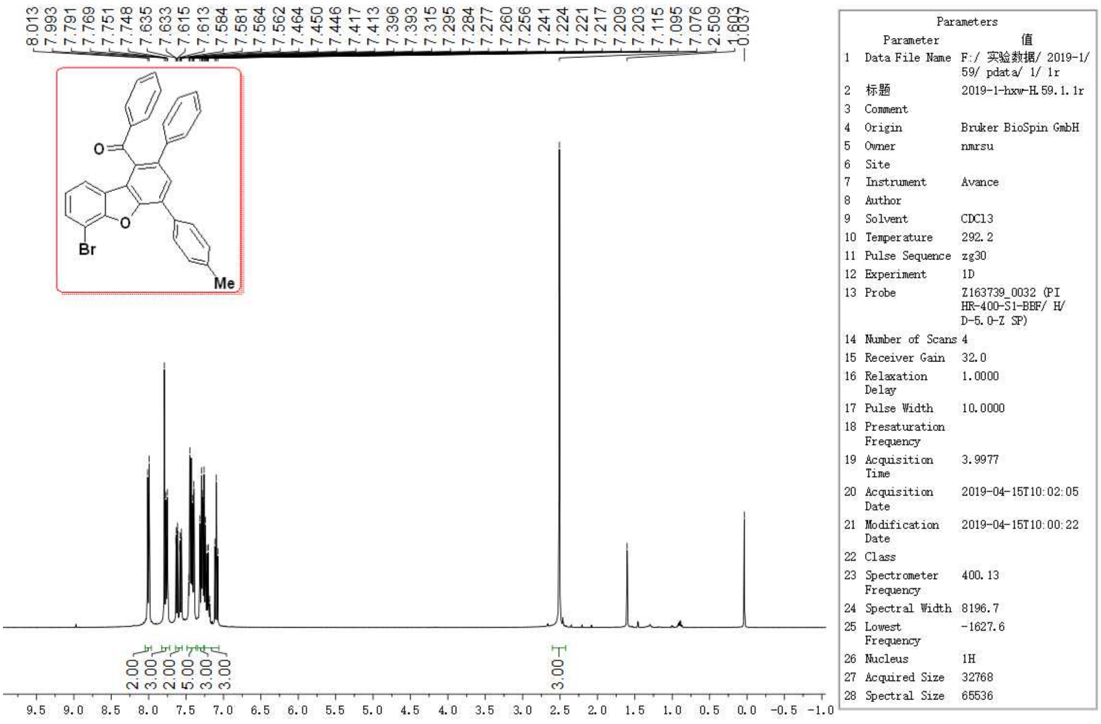

Supplementary Figure 31 ^{1}H NMR Spectra of compound 3oa

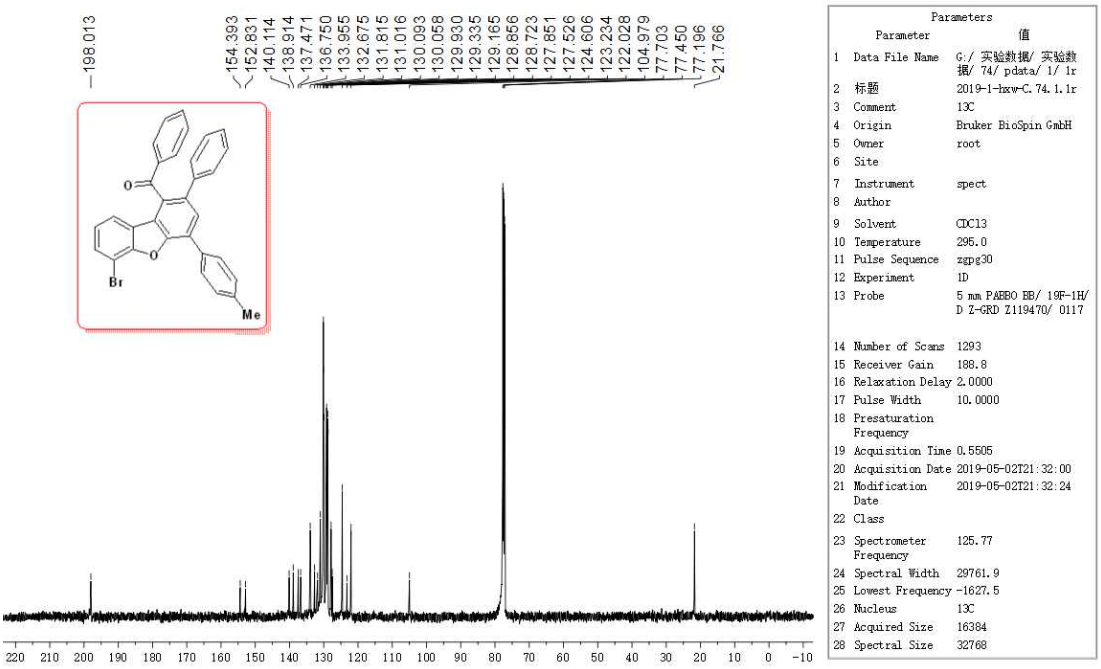

Supplementary Figure 32 ^{13}C NMR Spectra of compound 3oa

(6,8-Di-*tert*-butyl-2-phenyl-4-(*p*-tolyl)dibenzo[*b,d*]furan-1-yl)(phenyl)methanone (Figure 2, compound 3pa)

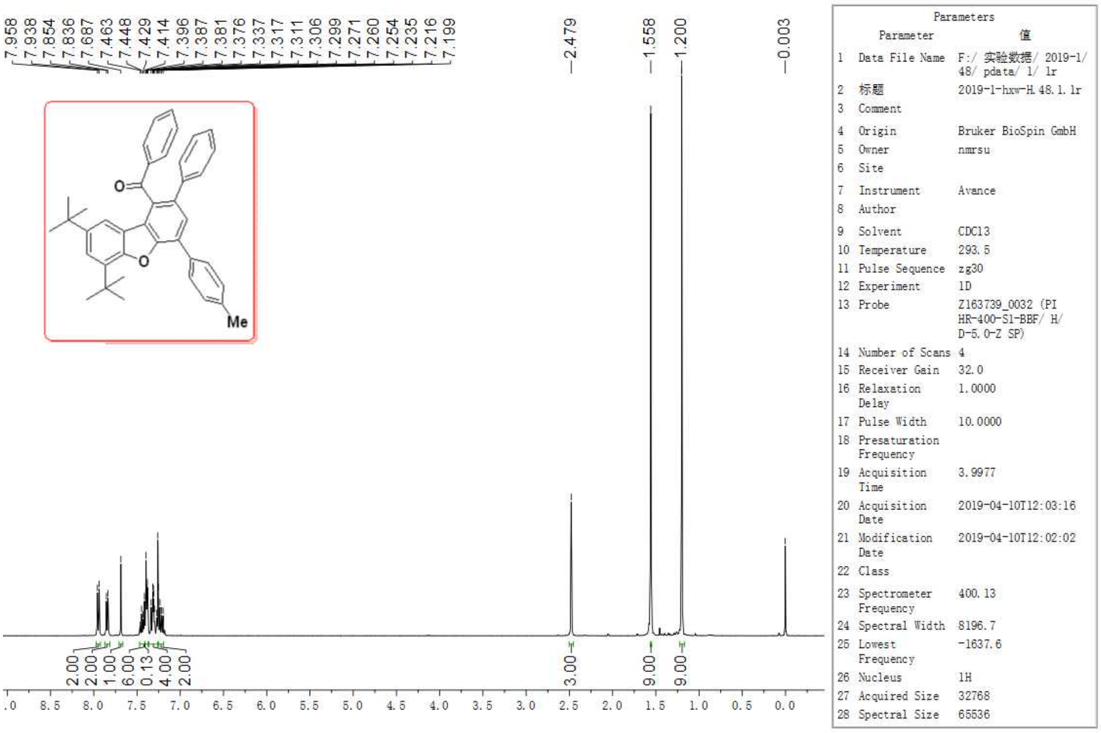

Supplementary Figure 33 ¹H NMR Spectra of compound 3pa

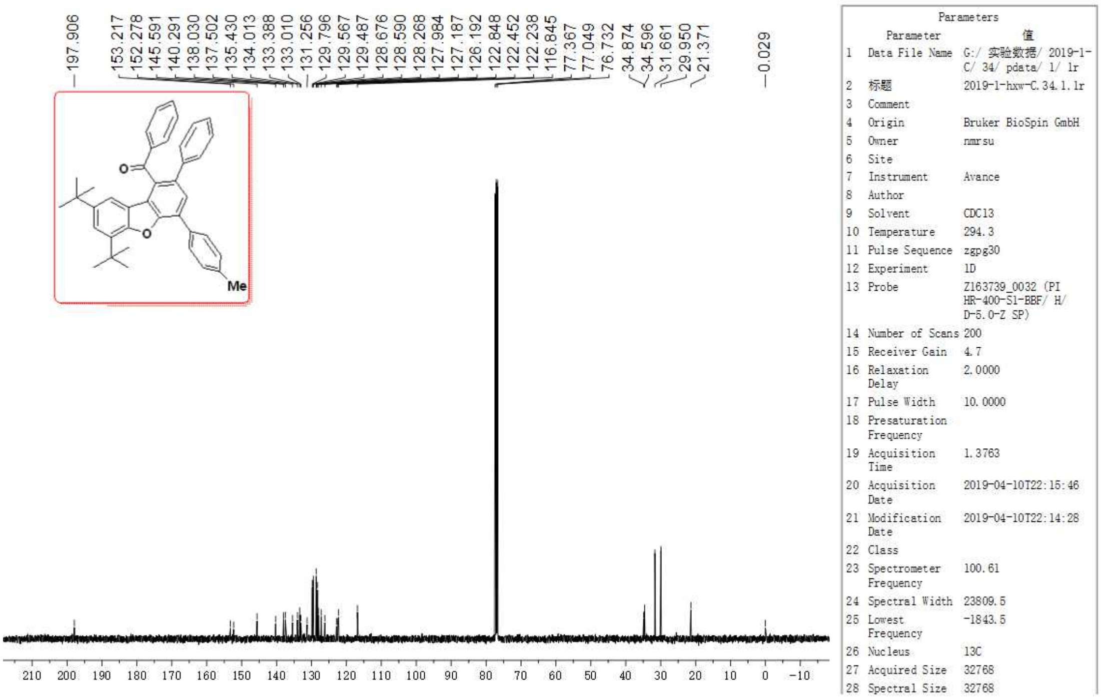

Supplementary Figure 34 ¹³C NMR Spectra of compound 3pa

(6-Bromo-8-chloro-4-(4-methoxyphenyl)-2-phenyldibenzo[*b,d*]furan-1-yl)(phenyl)methanone (Figure 2, compound 3qa)

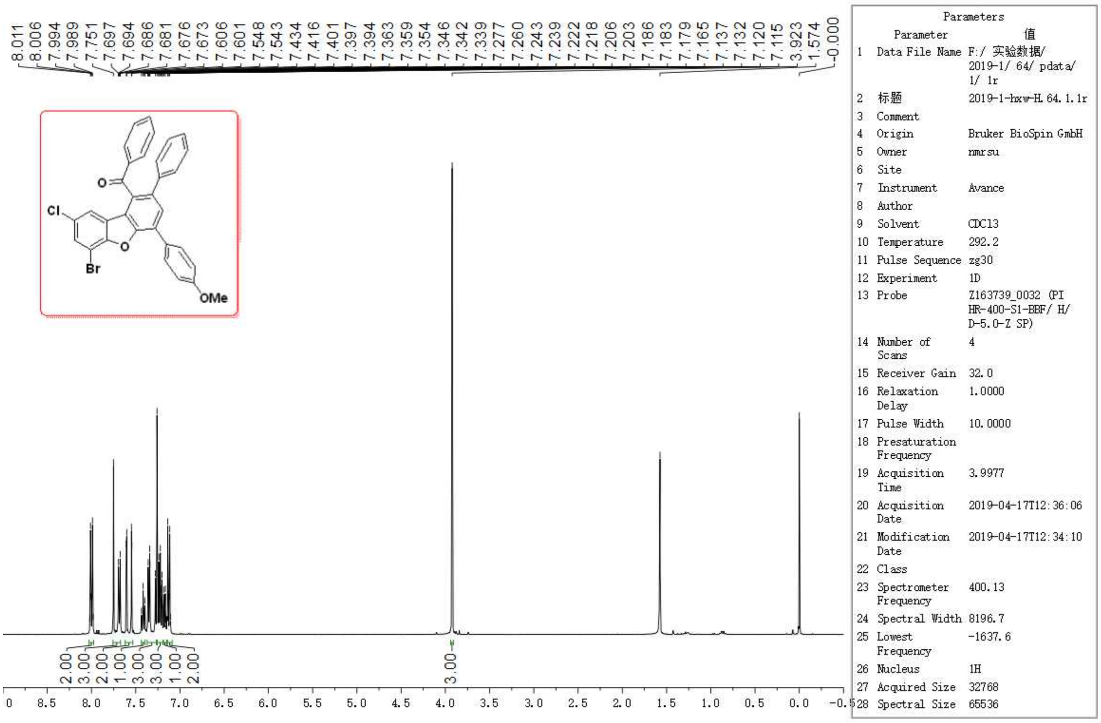

Supplementary Figure 35 ^{1}H NMR Spectra of compound 3qa

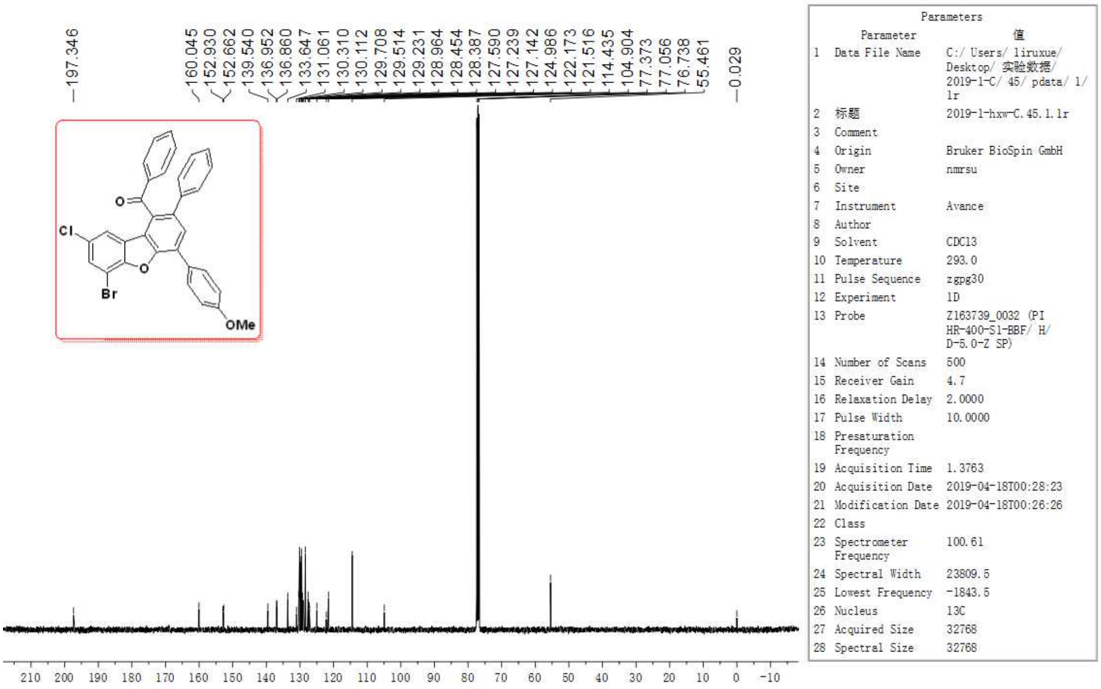

Supplementary Figure 36 ^{13}C NMR Spectra of compound 3qa

(8-Bromo-4-(cyclohex-1-en-1-yl)-2-phenyldibenzo[*b,d*]furan-1-yl)(phenyl)methanone (Figure 2, compound 3ra)

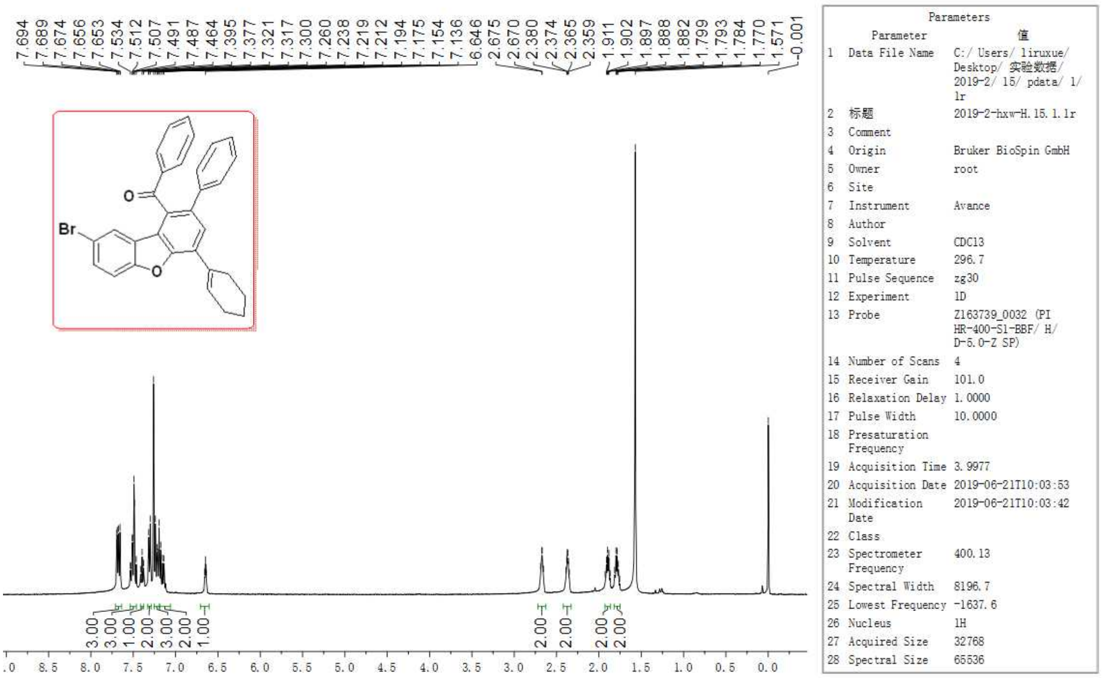

Supplementary Figure 37 ^{1}H NMR Spectra of compound 3ra

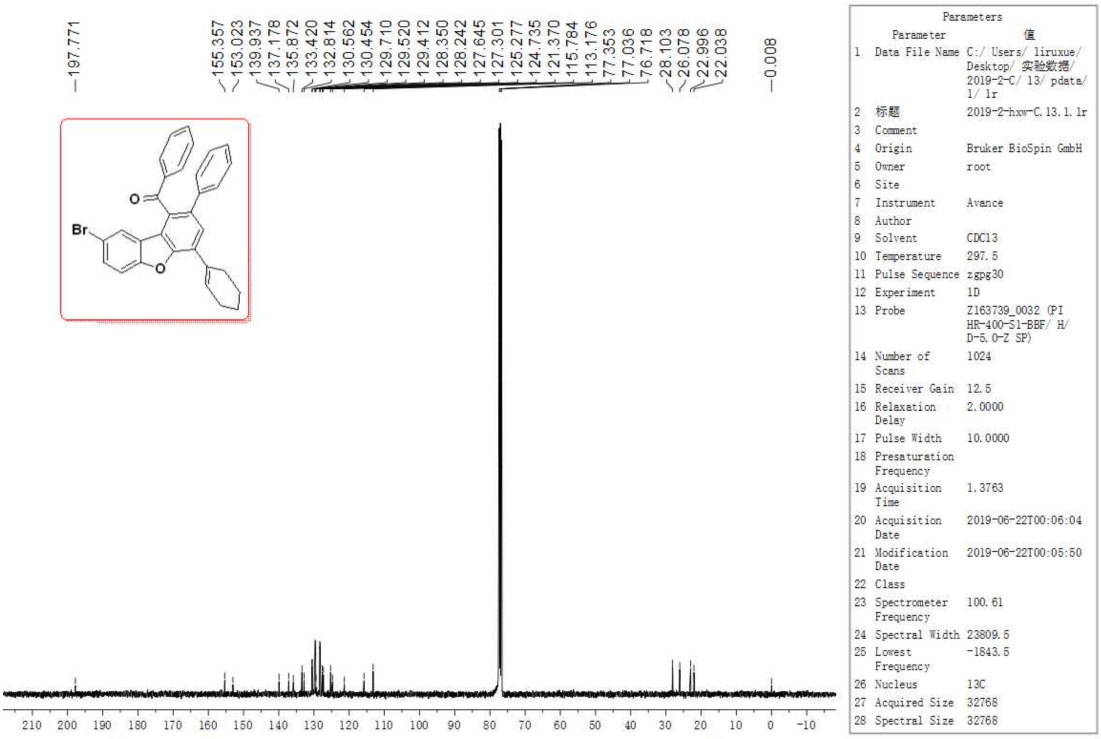

Supplementary Figure 38 ^{13}C NMR Spectra of compound 3ra

(8-Bromo-2-phenyl-4-(thiophen-3-yl)dibenzo[*b,d*]furan-1-yl)(phenyl)methanone (Figure 2, compound 3sa)

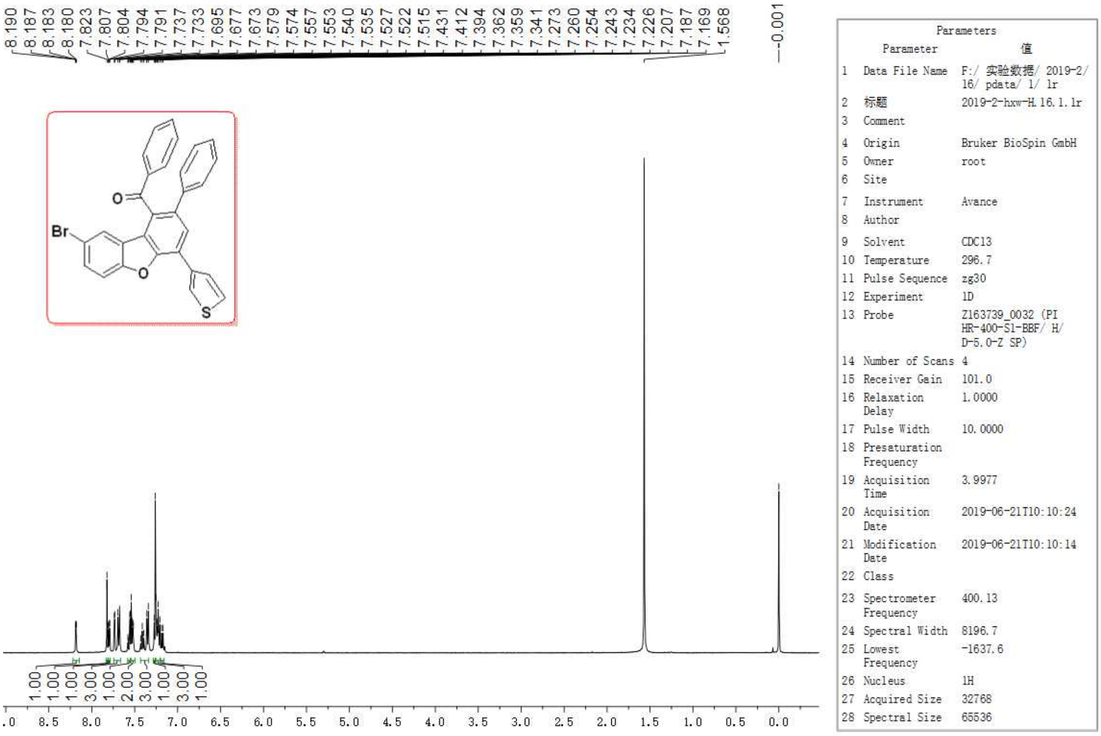

Supplementary Figure 39 ^{1}H NMR Spectra of compound 3sa

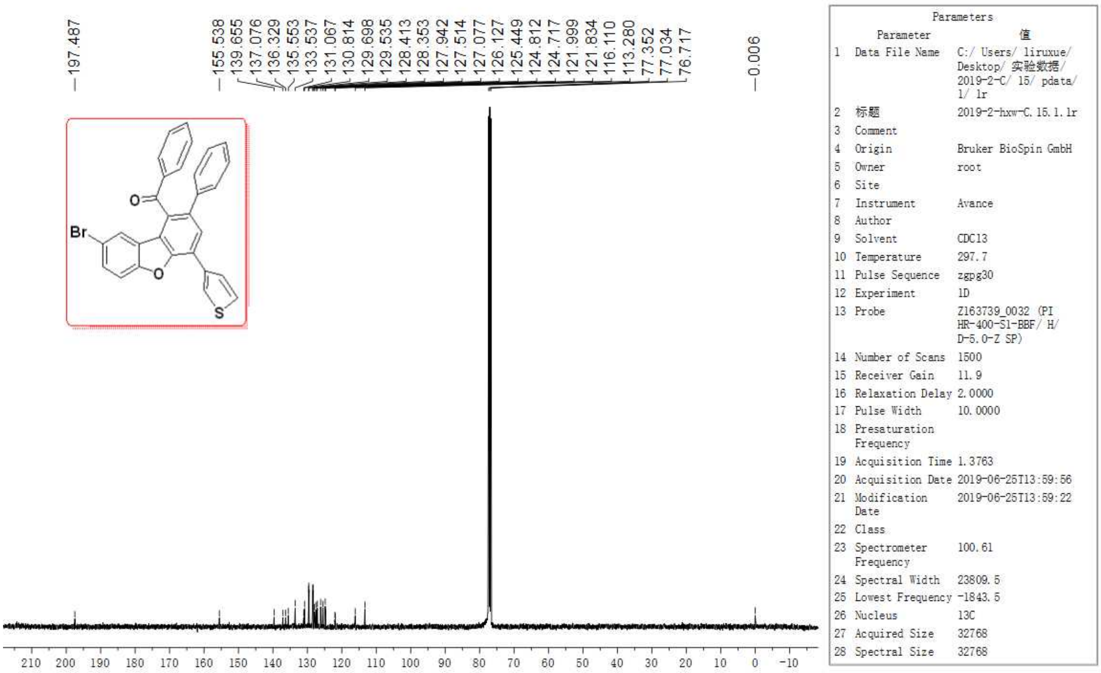

Supplementary Figure 40 ^{13}C NMR Spectra of compound 3sa

(4-Phenyl-2-(*o*-tolyl)dibenzo[b,d]furan-1-yl)(*o*-tolyl)methanone (Figure 3, compound 3ab)

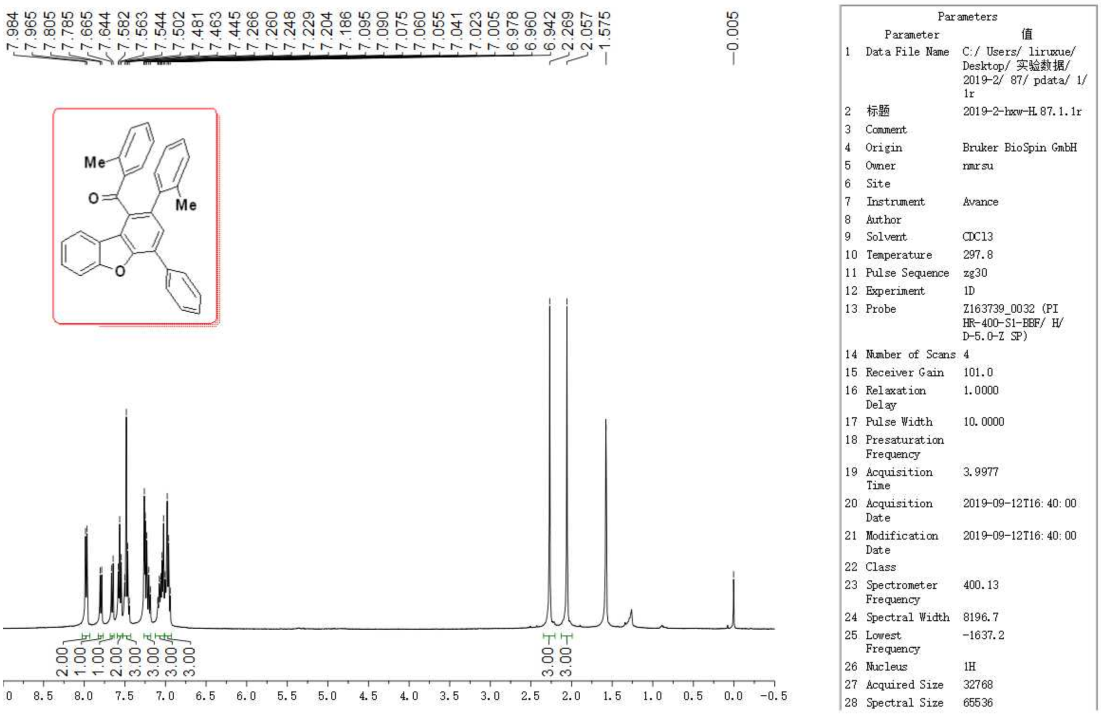

Supplementary Figure 41 ^{1}H NMR Spectra of compound 3ab

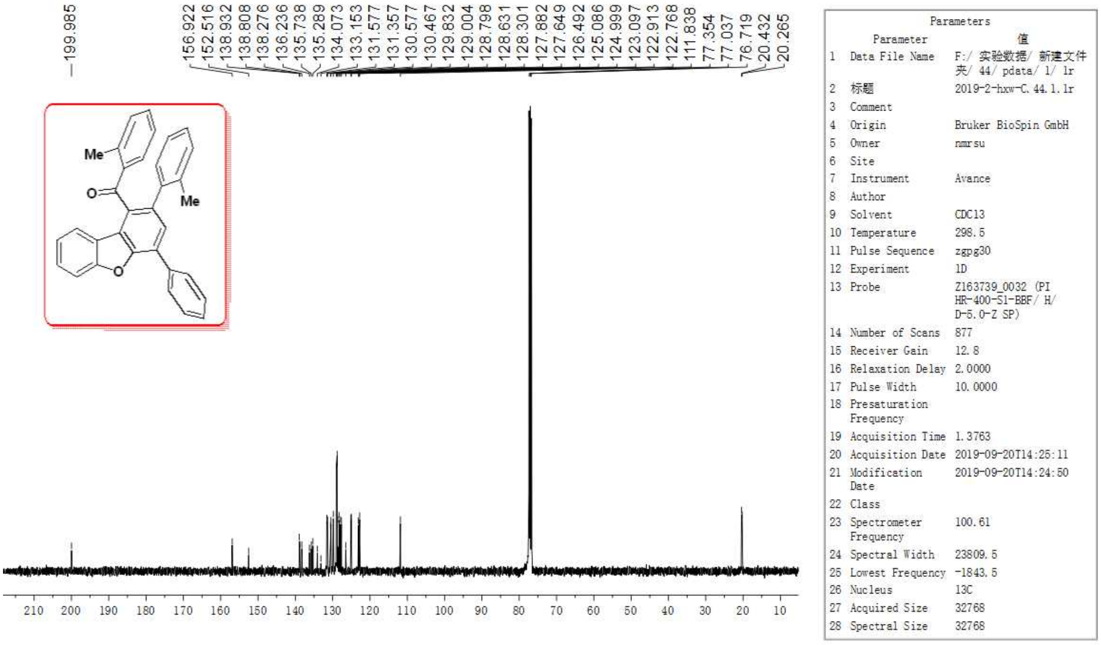

Supplementary Figure 42 ^{13}C NMR Spectra of compound 3ab

(4-Phenyl-2-(*m*-tolyl)dibenzo[*b,d*]furan-1-yl)(*m*-tolyl)methanone (Figure 3, compound 3ac)

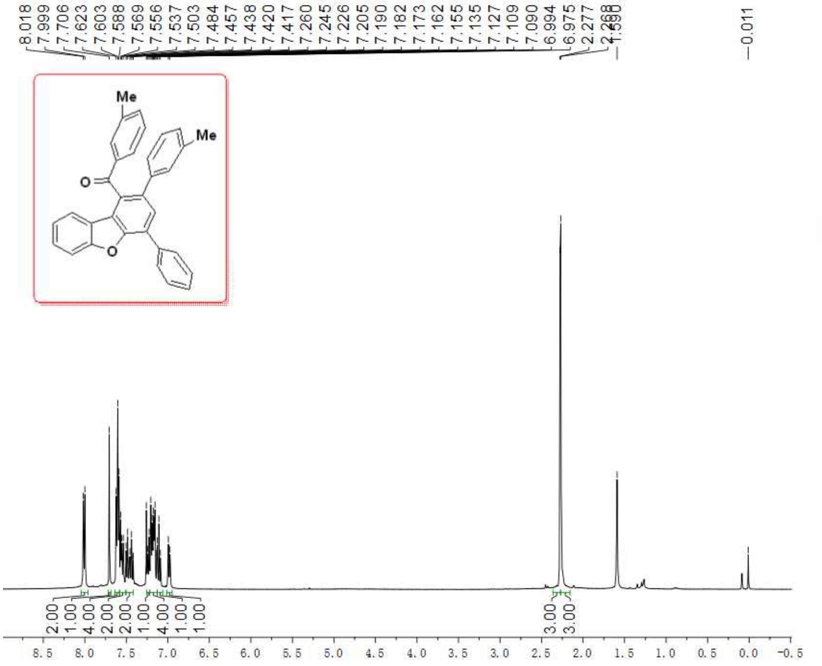

	Parameter	值
1	Data File Name	F:/ 实验数据/ 实验数据/ 2019-2/ 77/ pdata/ 1/ 1r
2	标题	2019-2-hxw-H. 77. 1. 1r
3	Comment	
4	Origin	Bruker BioSpin GmbH
5	Owner	root
6	Site	
7	Instrument	Avance
8	Author	
9	Solvent	CDCl3
10	Temperature	297.5
11	Pulse Sequence	zg30
12	Experiment	1D
13	Probe	Z163739_0032 (PI HR-400-S1-BBF/ H/ D-5.0-Z SP)
14	Number of Scans	4
15	Receiver Gain	101.0
16	Relaxation Delay	1.0000
17	Pulse Width	10.0000
18	Presaturation Frequency	
19	Acquisition Time	3.9977
20	Acquisition Date	2019-08-27T11:13:51
21	Modification Date	2019-08-27T11:12:50
22	Class	
23	Spectrometer Frequency	400.13
24	Spectral Width	8196.7
25	Lowest Frequency	-1637.6
26	Nucleus	1H
27	Acquired Size	32768
28	Spectral Size	65536

Supplementary Figure 43 ^{1}H NMR Spectra of compound 3ac

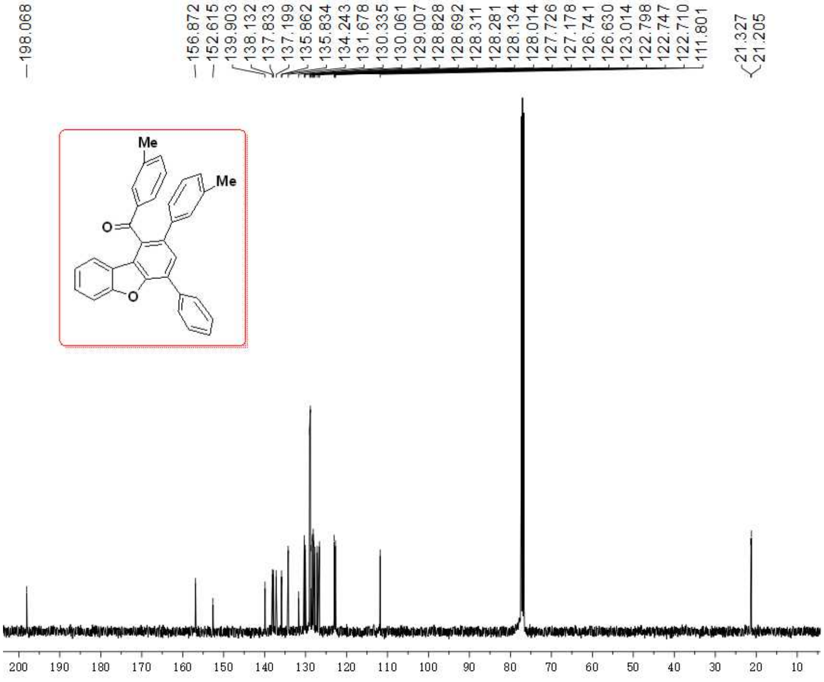

	Parameter	值
1	Data File Name	C:/ Users/ liruxue/ Desktop/ 实验数据/ 2019-2-C/ 35/ pdata/ 1/ 1r
2	标题	2019-2-hxw-C. 35. 1. 1r
3	Comment	
4	Origin	Bruker BioSpin GmbH
5	Owner	root
6	Site	
7	Instrument	Avance
8	Author	
9	Solvent	CDCl3
10	Temperature	298.8
11	Pulse Sequence	zgpg30
12	Experiment	1D
13	Probe	Z163739_0032 (PI HR-400-S1-BBF/ H/ D-5.0-Z SP)
14	Number of Scans	500
15	Receiver Gain	12.5
16	Relaxation Delay	2.0000
17	Pulse Width	10.0000
18	Presaturation Frequency	
19	Acquisition Time	1.3763
20	Acquisition Date	2019-09-03T02:54:30
21	Modification Date	2019-09-03T02:52:50
22	Class	
23	Spectrometer Frequency	100.61
24	Spectral Width	23809.5
25	Lowest Frequency	-1843.5
26	Nucleus	13C
27	Acquired Size	32768
28	Spectral Size	32768

Supplementary Figure 44 ^{13}C NMR Spectra of compound 3ac

(4-Phenyl-2-(*p*-tolyl)dibenzo[*b,d*]furan-1-yl)(*p*-tolyl)methanone (Figure 3, compound 3ad)

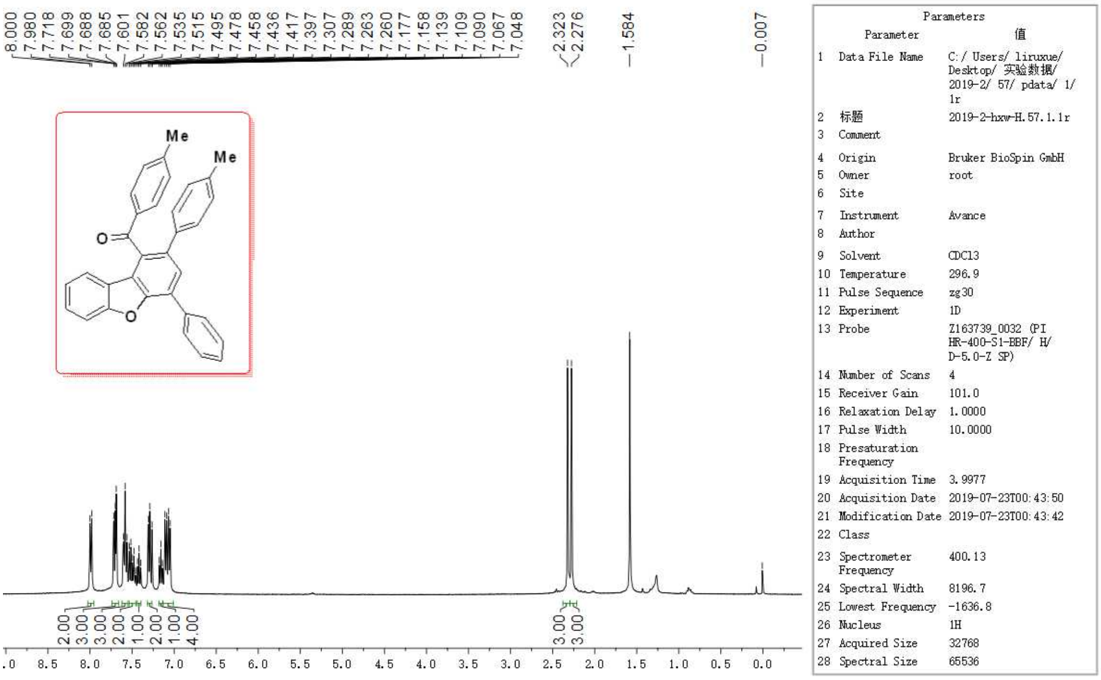

Supplementary Figure 45 ^{1}**H NMR Spectra of compound 3ad**

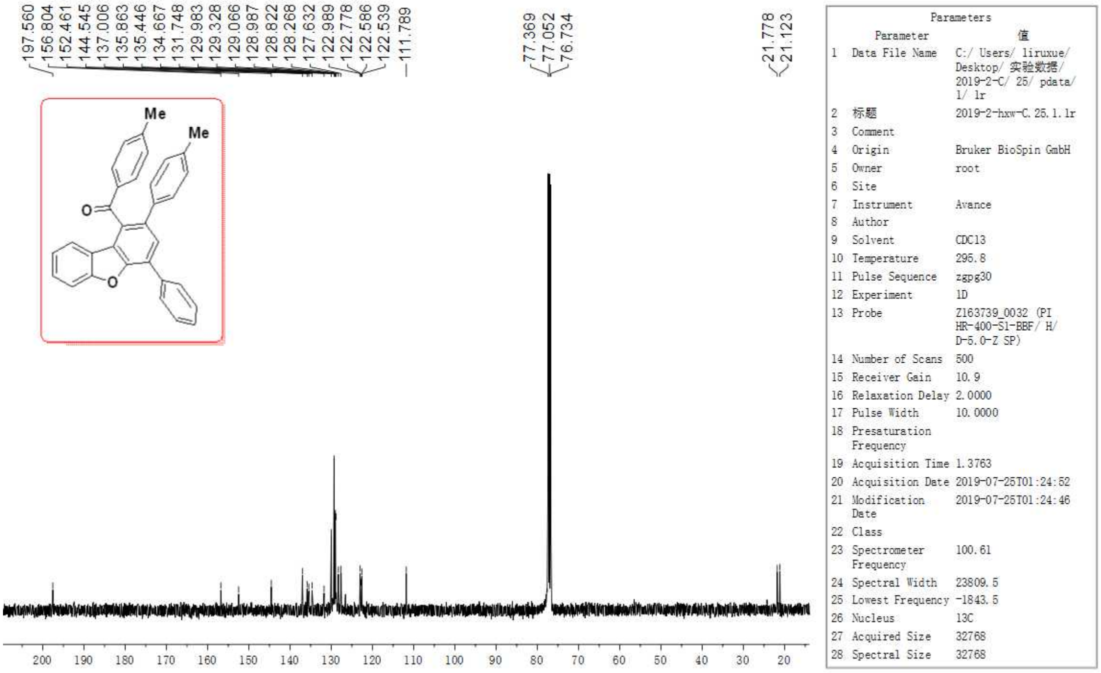

Supplementary Figure 46 ^{13}**C NMR Spectra of compound 3ad**

(4-Ethylphenyl)(2-(4-ethylphenyl)-4-phenyldibenzo[*b,d*]furan-1-yl)methanone (Figure 3, compound 3ae)

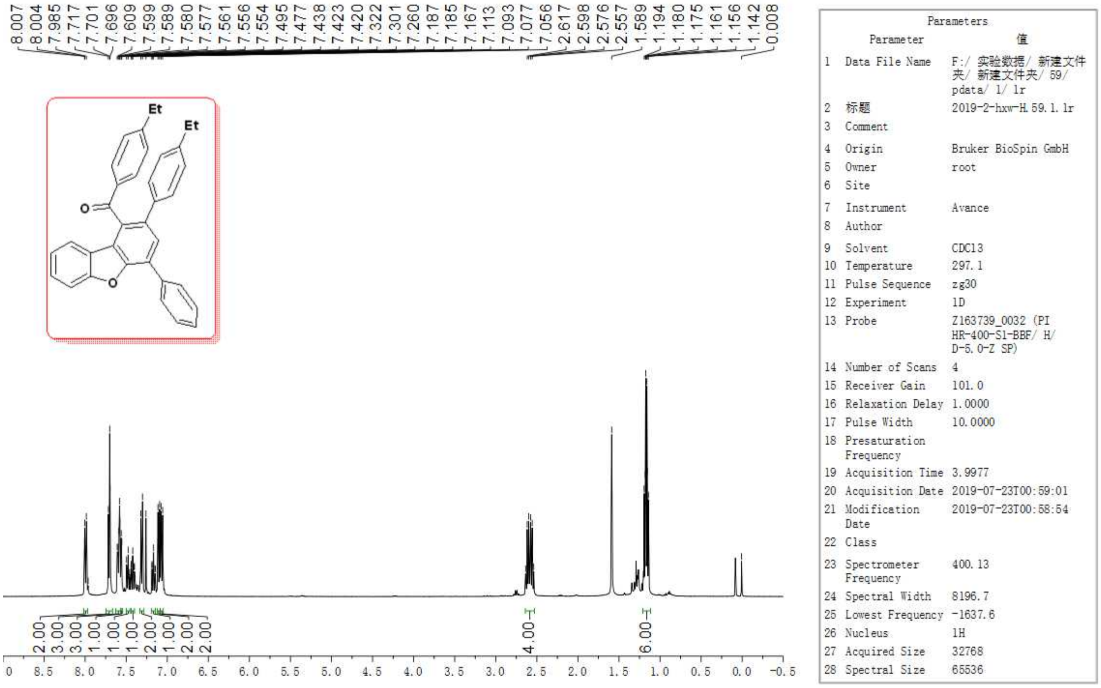

Supplementary Figure 47 ^{1}H NMR Spectra of compound 3ae

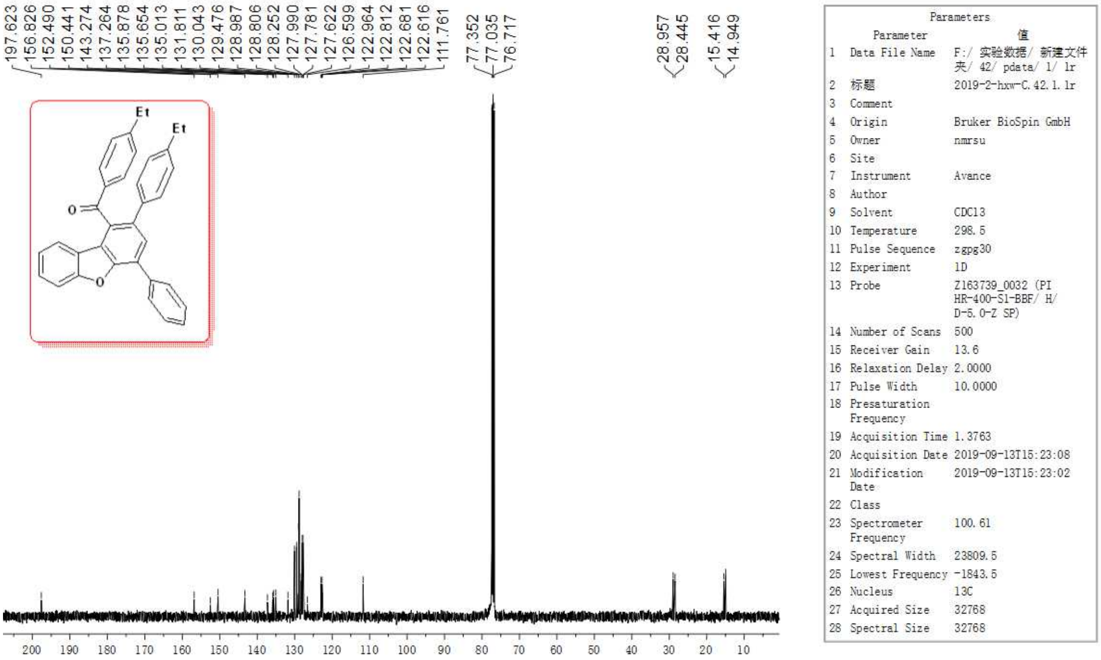

Supplementary Figure 48 ^{13}C NMR Spectra of compound 3ae

(4-Methoxyphenyl)(2-(4-methoxyphenyl)-4-phenyldibenzo[*b,d*]furan-1-yl)methanone (Figure 3, compound 3af)

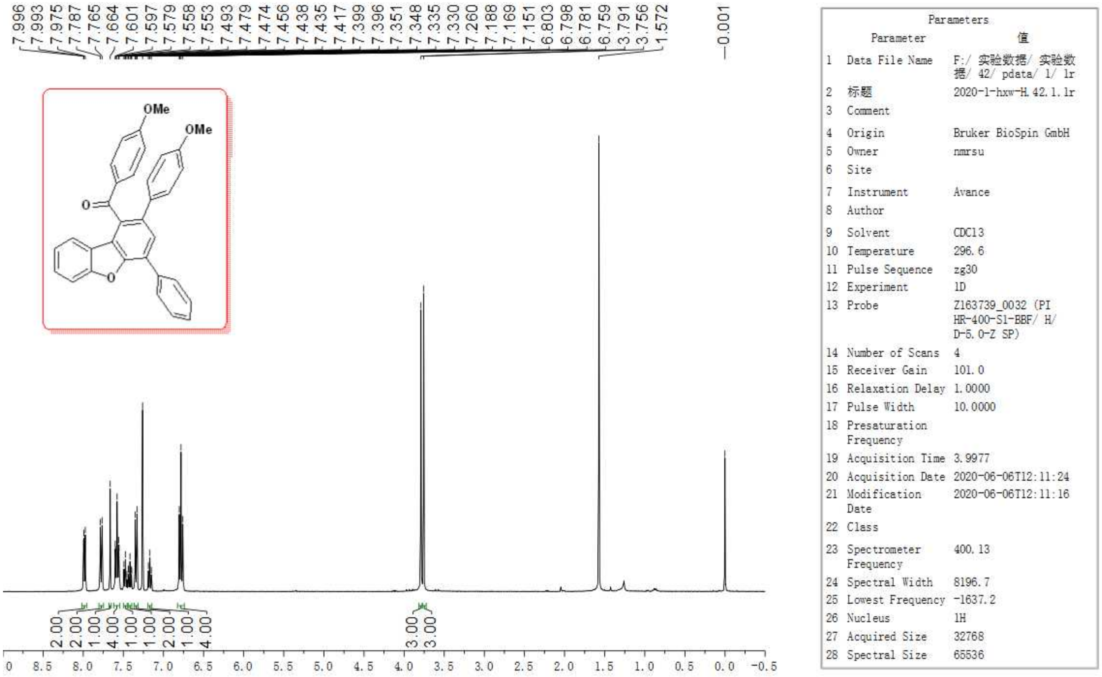

Supplementary Figure 49 ^{1}H NMR Spectra of compound 3af

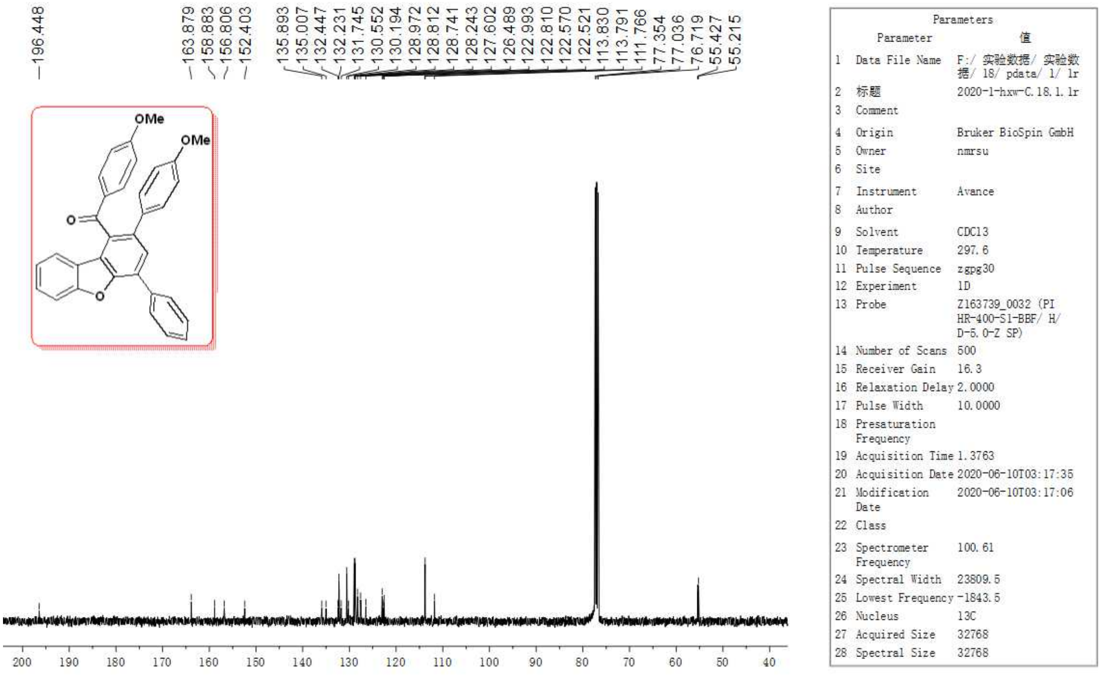

Supplementary Figure 50 ^{13}C NMR Spectra of compound 3af

(3-Fluorophenyl)(2-(3-fluorophenyl)-4-phenyldibenzo[*b,d*]furan-1-yl)methanone (Figure 3, compound 3ag)

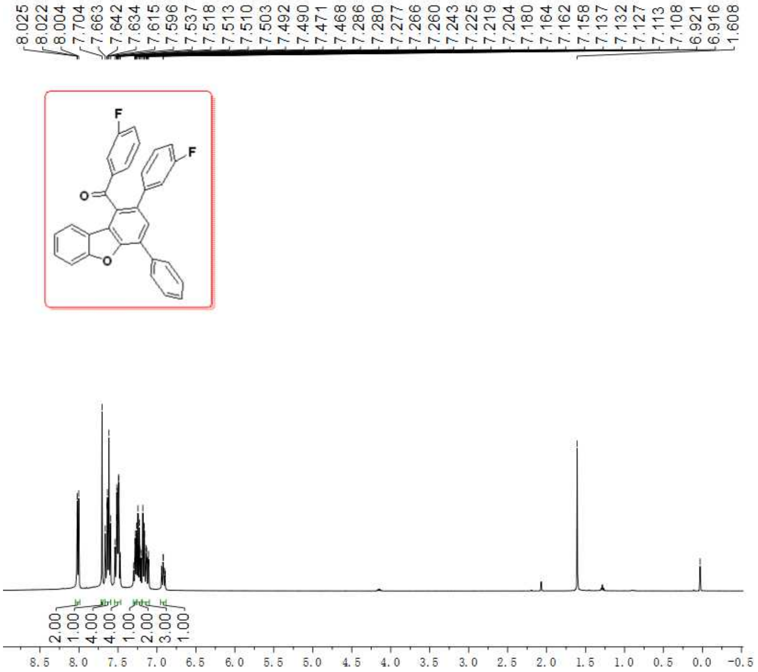

Parameters		
	Parameter	值
1	Data File Name	C:/ Users/ liruxue/ Desktop/ 实验数据/ 2019-2/ 67/ pdata/ 1/ 1r
2	标题	2019-2-hxw-H.67.1.1r
3	Comment	
4	Origin	Bruker BioSpin GmbH
5	Owner	root
6	Site	
7	Instrument	Avance
8	Author	
9	Solvent	CDCl3
10	Temperature	297.9
11	Pulse Sequence	zg30
12	Experiment	1D
13	Probe	Z163739_0032 (PI HR-400-S1-BBF/ H/ D-5.0-Z SP)
14	Number of Scans	4
15	Receiver Gain	101.0
16	Relaxation Delay	1.0000
17	Pulse Width	10.0000
18	Presaturation Frequency	
19	Acquisition Time	3.9977
20	Acquisition Date	2019-07-28T08:58:54
21	Modification Date	2019-07-28T08:58:28
22	Class	
23	Spectrometer Frequency	400.13
24	Spectral Width	8196.7
25	Lowest Frequency	-1630.8
26	Nucleus	1H
27	Acquired Size	32768
28	Spectral Size	65536

Supplementary Figure 51 ^{1}H NMR Spectra of compound 3ag

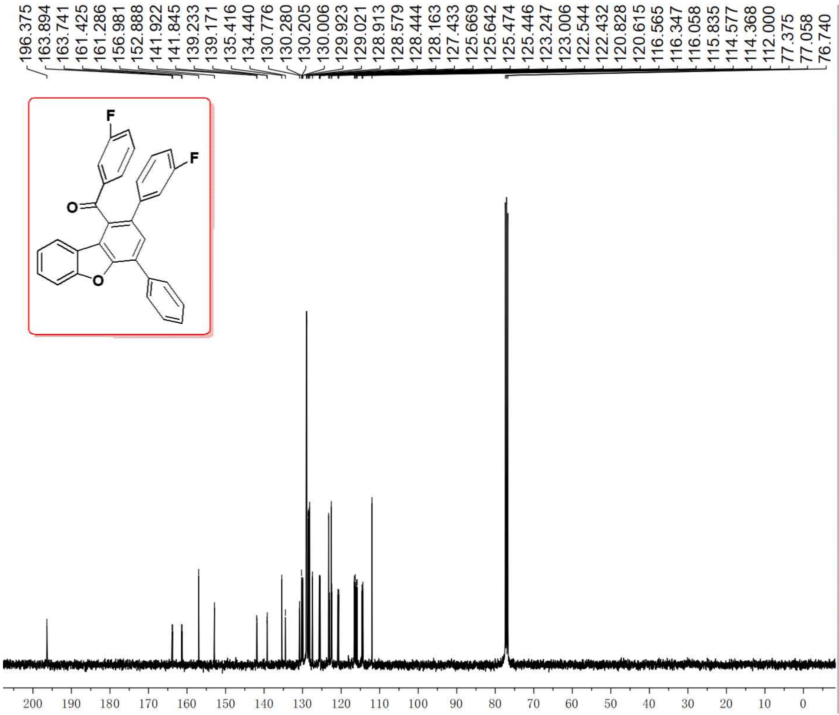

Parameters		
	Parameter	值
1	Data File Name	C:/ Users/ liruxue/ Desktop/ 实验数据/ 2019-2-C/ 27/ pdata/ 1/ 1r
2	标题	2019-2-hxw-C.27.1.1r
3	Comment	
4	Origin	Bruker BioSpin GmbH
5	Owner	root
6	Site	
7	Instrument	Avance
8	Author	
9	Solvent	CDCl3
10	Temperature	299.2
11	Pulse Sequence	zgpg30
12	Experiment	1D
13	Probe	Z163739_0032 (PI HR-400-S1-BBF/ H/ D-5.0-Z SP)
14	Number of Scans	500
15	Receiver Gain	11.6
16	Relaxation Delay	2.0000
17	Pulse Width	10.0000
18	Presaturation Frequency	
19	Acquisition Time	1.3763
20	Acquisition Date	2019-07-30T07:10:20
21	Modification Date	2019-07-30T07:09:44
22	Class	
23	Spectrometer Frequency	100.61
24	Spectral Width	23809.5
25	Lowest Frequency	-1843.5
26	Nucleus	13C
27	Acquired Size	32768
28	Spectral Size	32768

Supplementary Figure 52 ^{13}C NMR Spectra of compound 3ag

(2-Chlorophenyl)(2-(2-chlorophenyl)-4-phenyldibenzo[*b,d*]furan-1-yl)methanone (Figure 3, compound 3ah)

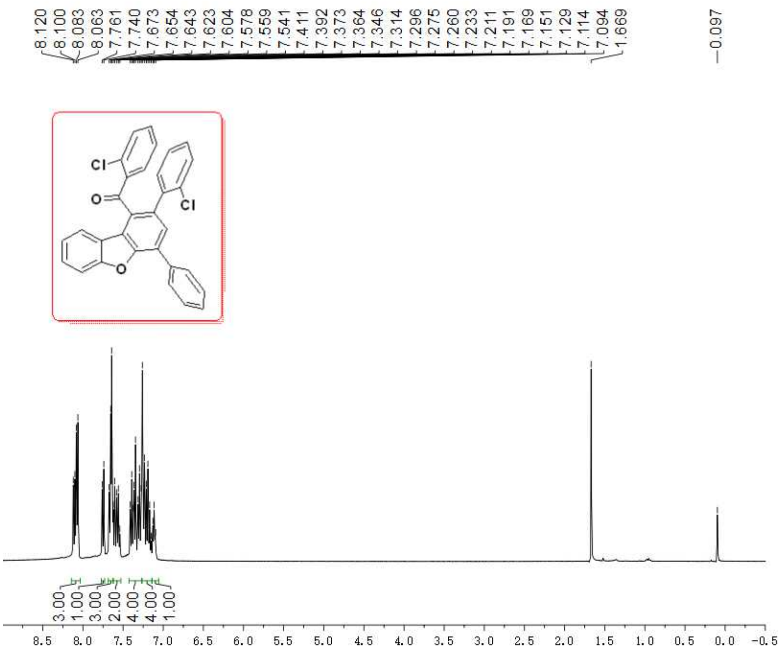

Parameters		
	Parameter	值
1	Data File Name	C:/ Users/ liruxue/ Desktop/ 实验数据/ 2019-2/ 36/ pdata/ 1/ 1r
2	标题	2019-2-hxw-H.36.1.1r
3	Comment	
4	Origin	Bruker BioSpin GmbH
5	Owner	root
6	Site	
7	Instrument	Avance
8	Author	
9	Solvent	CDCl3
10	Temperature	297.0
11	Pulse Sequence	zg30
12	Experiment	1D
13	Probe	Z163739_0032 (PI HR-400-S1-BBF/ H/ D-5.0-Z SP)
14	Number of Scans	4
15	Receiver Gain	101.0
16	Relaxation Delay	1.0000
17	Pulse Width	10.0000
18	Presaturation Frequency	
19	Acquisition Time	3.9977
20	Acquisition Date	2019-07-08T11:24:39
21	Modification Date	2019-07-08T11:23:50
22	Class	
23	Spectrometer Frequency	400.13
24	Spectral Width	8196.7
25	Lowest Frequency	-1602.8
26	Nucleus	1H
27	Acquired Size	32768
28	Spectral Size	65536

Supplementary Figure 53 ^{1}H NMR Spectra of compound 3ah

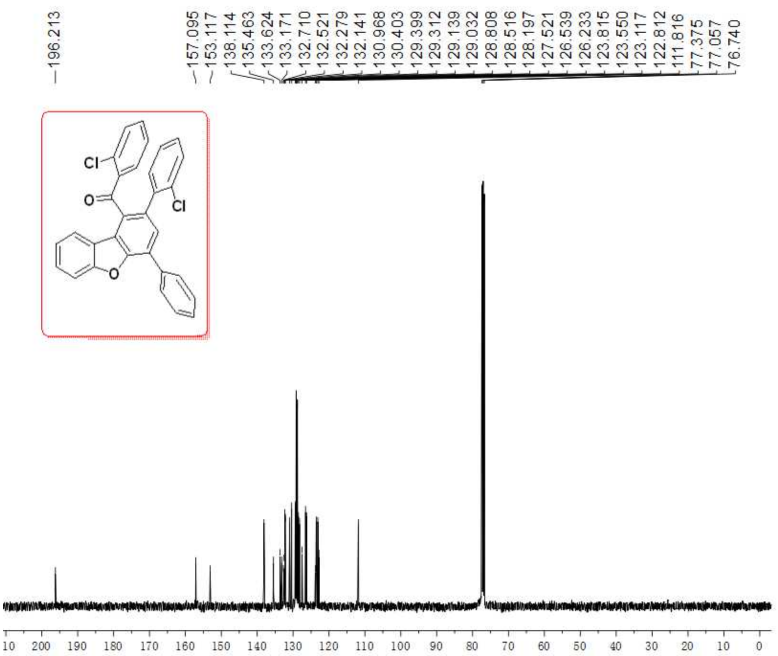

Parameters		
	Parameter	值
1	Data File Name	C:/ Users/ liruxue/ Desktop/ 实验数据/ 2019-2-C/ 18/ pdata/ 1/ 1r
2	标题	2019-2-hxw-C.18.1.1r
3	Comment	
4	Origin	Bruker BioSpin GmbH
5	Owner	root
6	Site	
7	Instrument	Avance
8	Author	
9	Solvent	CDCl3
10	Temperature	297.7
11	Pulse Sequence	zgpg30
12	Experiment	1D
13	Probe	Z163739_0032 (PI HR-400-S1-BBF/ H/ D-5.0-Z SP)
14	Number of Scans	500
15	Receiver Gain	15.3
16	Relaxation Delay	2.0000
17	Pulse Width	10.0000
18	Presaturation Frequency	
19	Acquisition Time	1.3763
20	Acquisition Date	2019-07-11T16:48:40
21	Modification Date	2019-07-11T16:47:34
22	Class	
23	Spectrometer Frequency	100.61
24	Spectral Width	23809.5
25	Lowest Frequency	-1843.5
26	Nucleus	13C
27	Acquired Size	32768
28	Spectral Size	32768

Supplementary Figure 54 ^{13}C NMR Spectra of compound 3ah

(3-Chlorophenyl)(2-(3-chlorophenyl)-4-phenyldibenzo[*b,d*]furan-1-yl)methanone (Figure 3, compound 3ai)

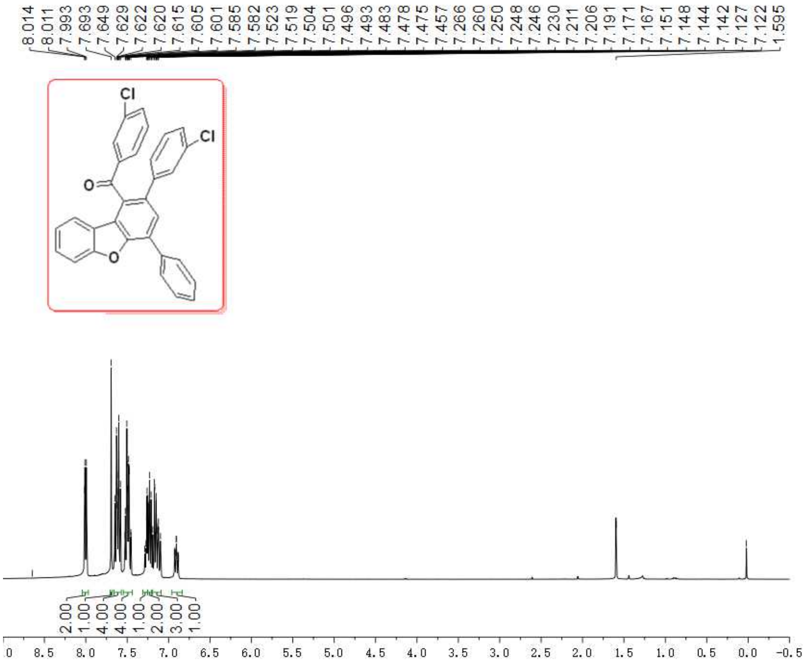

Parameters		
	Parameter	值
1	Data File Name	C:/ Users/ liruxue/ Desktop/ 实验数据/ 2019-2/ 79/ pdata/ 1/ 1r
2	标题	2019-2-hxw-H. 79. 1. 1r
3	Comment	
4	Origin	Bruker BioSpin GmbH
5	Owner	root
6	Site	
7	Instrument	Avance
8	Author	
9	Solvent	CDCl3
10	Temperature	296.9
11	Pulse Sequence	zg30
12	Experiment	1D
13	Probe	Z163739_0032 (PI HR-400-S1-BBF/ H/ D-5.0-Z SP)
14	Number of Scans	4
15	Receiver Gain	101.0
16	Relaxation Delay	1.0000
17	Pulse Width	10.0000
18	Presaturation Frequency	
19	Acquisition Time	3.9977
20	Acquisition Date	2019-08-30T20:57:58
21	Modification Date	2019-08-30T20:56:36
22	Class	
23	Spectrometer Frequency	400.13
24	Spectral Width	8196.7
25	Lowest Frequency	-1637.6
26	Nucleus	1H
27	Acquired Size	32768
28	Spectral Size	65536

Supplementary Figure 55 ^{1}**H NMR Spectra of compound 3ai**

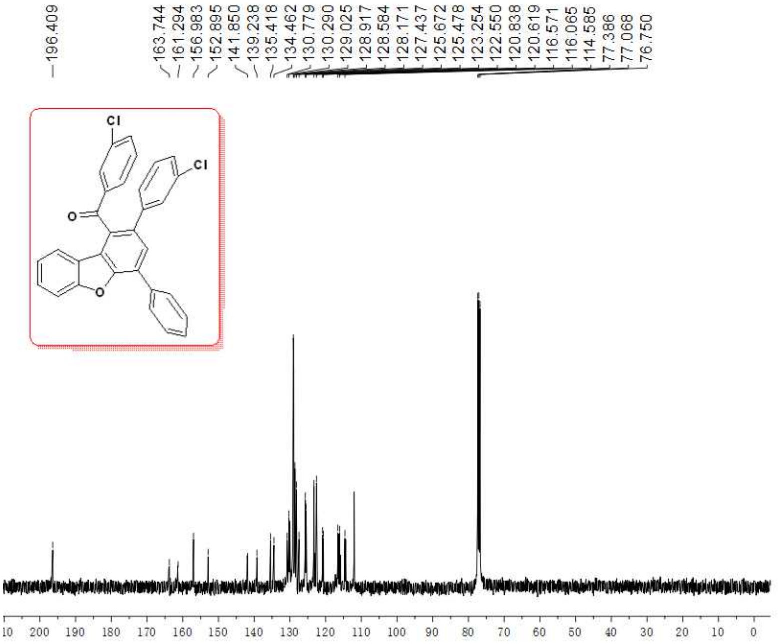

Parameters		
	Parameter	值
1	Data File Name	C:/ Users/ liruxue/ Desktop/ 实验数据/ 2019-2-C/ 36/ pdata/ 1/ 1r
2	标题	2019-2-hxw-C. 36. 1. 1r
3	Comment	
4	Origin	Bruker BioSpin GmbH
5	Owner	root
6	Site	
7	Instrument	Avance
8	Author	
9	Solvent	CDCl3
10	Temperature	298.7
11	Pulse Sequence	zgpg30
12	Experiment	1D
13	Probe	Z163739_0032 (PI HR-400-S1-BBF/ H/ D-5.0-Z SP)
14	Number of Scans	500
15	Receiver Gain	11.2
16	Relaxation Delay	2.0000
17	Pulse Width	10.0000
18	Presaturation Frequency	
19	Acquisition Time	1.3763
20	Acquisition Date	2019-09-03T15:16:27
21	Modification Date	2019-09-03T15:14:44
22	Class	
23	Spectrometer Frequency	100.61
24	Spectral Width	23809.5
25	Lowest Frequency	-1843.5
26	Nucleus	13C
27	Acquired Size	32768
28	Spectral Size	32768

Supplementary Figure 56 ^{13}**C NMR Spectra of compound 3ai**

(2-Bromophenyl)(2-(2-bromophenyl)-4-phenyldibenzo[*b,d*]furan-1-yl)methanone (Figure 3, compound 3aj)

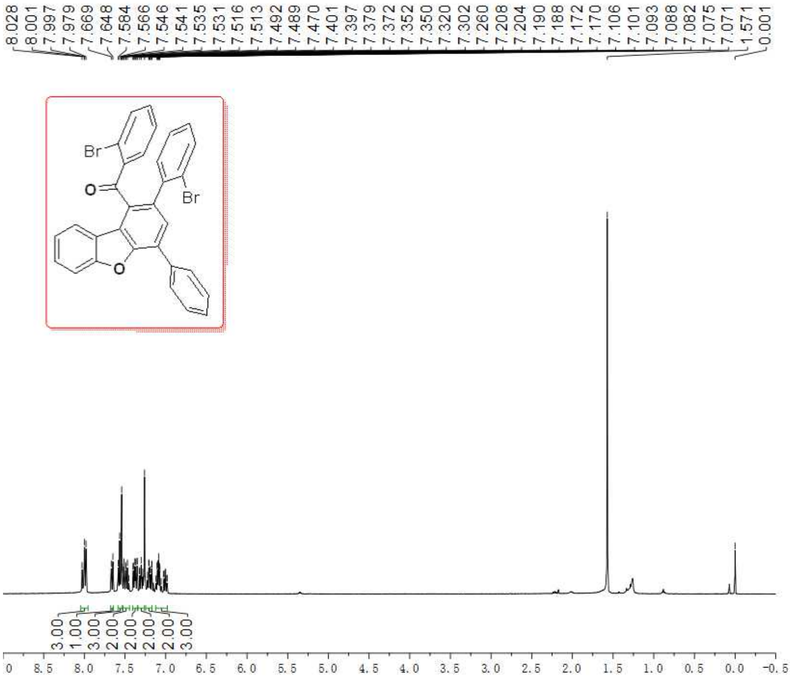

Parameters

	Parameter	值
1	Data File Name	C:/ Users/ liruxue/ Desktop/ 实验数据/ 2019-2/ 68/ pdata/ 1/ 1r
2	标题	2019-2-hxw-H.68.1.1r
3	Comment	
4	Origin	Bruker BioSpin GmbH
5	Owner	root
6	Site	
7	Instrument	Avance
8	Author	
9	Solvent	CDCl3
10	Temperature	297.9
11	Pulse Sequence	zg30
12	Experiment	1D
13	Probe	Z163739_0032 (PI HR-400-S1-BBF/ H/ D-5.0-Z SP)
14	Number of Scans	4
15	Receiver Gain	101.0
16	Relaxation Delay	1.0000
17	Pulse Width	10.0000
18	Presaturation Frequency	
19	Acquisition Time	3.9977
20	Acquisition Date	2019-07-28T09:04:31
21	Modification Date	2019-07-28T09:04:06
22	Class	
23	Spectrometer Frequency	400.13
24	Spectral Width	8196.7
25	Lowest Frequency	-1637.6
26	Nucleus	1H
27	Acquired Size	32768
28	Spectral Size	65536

Supplementary Figure 57 [1]**H NMR Spectra of compound 3aj**

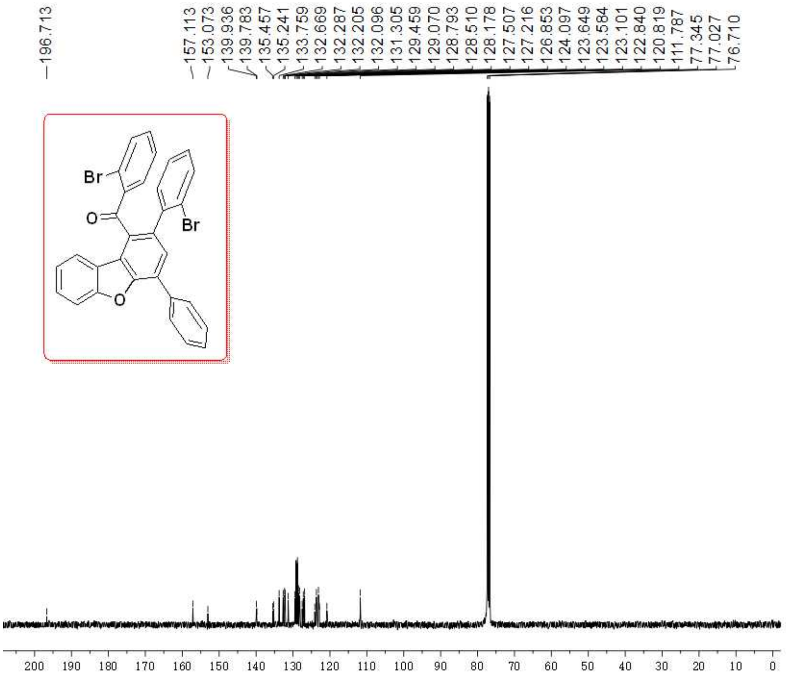

Parameters

	Parameter	值
1	Data File Name	F:/ 实验数据/ 实验数据/ 2019-2-C/ 28/ pdata/ 1/ 1r
2	标题	2019-2-hxw-C.28.1.1r
3	Comment	
4	Origin	Bruker BioSpin GmbH
5	Owner	root
6	Site	
7	Instrument	Avance
8	Author	
9	Solvent	CDCl3
10	Temperature	299.9
11	Pulse Sequence	zgpg30
12	Experiment	1D
13	Probe	Z163739_0032 (PI HR-400-S1-BBF/ H/ D-5.0-Z SP)
14	Number of Scans	2000
15	Receiver Gain	13.2
16	Relaxation Delay	2.0000
17	Pulse Width	10.0000
18	Presaturation Frequency	
19	Acquisition Time	1.3763
20	Acquisition Date	2019-07-31T14:09:54
21	Modification Date	2019-07-31T14:09:10
22	Class	
23	Spectrometer Frequency	100.61
24	Spectral Width	23809.5
25	Lowest Frequency	-1843.5
26	Nucleus	13C
27	Acquired Size	32768
28	Spectral Size	32768

Supplementary Figure 58 [13]**C NMR Spectra of compound 3aj**

(3-Bromophenyl)(2-(3-bromophenyl)-4-phenyldibenzo[*b,d*]furan-1-yl)methanone (Figure 3, compound 3ak)

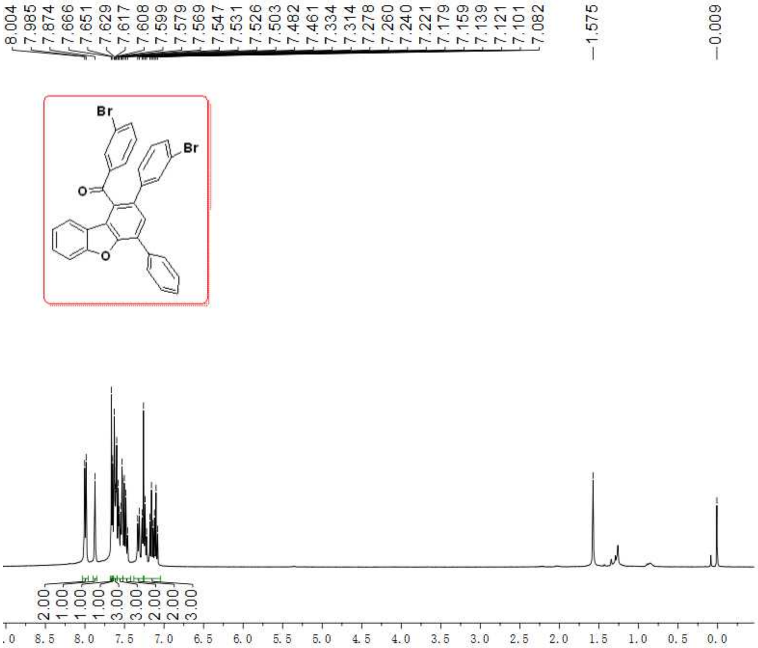

Parameters

	Parameter	值
1	Data File Name	C:/ Users/ liruxue/ Desktop/ 实验数据/ 2019-2/ 75/ pdata/ 1/ 1r
2	标题	2019-2-hxw-H. 75. 1. 1r
3	Comment	
4	Origin	Bruker BioSpin GmbH
5	Owner	root
6	Site	
7	Instrument	Avance
8	Author	
9	Solvent	CDCl3
10	Temperature	296.9
11	Pulse Sequence	zg30
12	Experiment	1D
13	Probe	Z163739_0032 (PI HR-400-S1-BBF/ H/ D-5.0-Z SP)
14	Number of Scans	4
15	Receiver Gain	101.0
16	Relaxation Delay	1.0000
17	Pulse Width	10.0000
18	Presaturation Frequency	
19	Acquisition Time	3.9977
20	Acquisition Date	2019-08-26T11:50:45
21	Modification Date	2019-08-26T11:49:48
22	Class	
23	Spectrometer Frequency	400.13
24	Spectral Width	8196.7
25	Lowest Frequency	-1637.2
26	Nucleus	1H
27	Acquired Size	32768
28	Spectral Size	65536

Supplementary Figure 59 ^{1}H NMR Spectra of compound 3ak

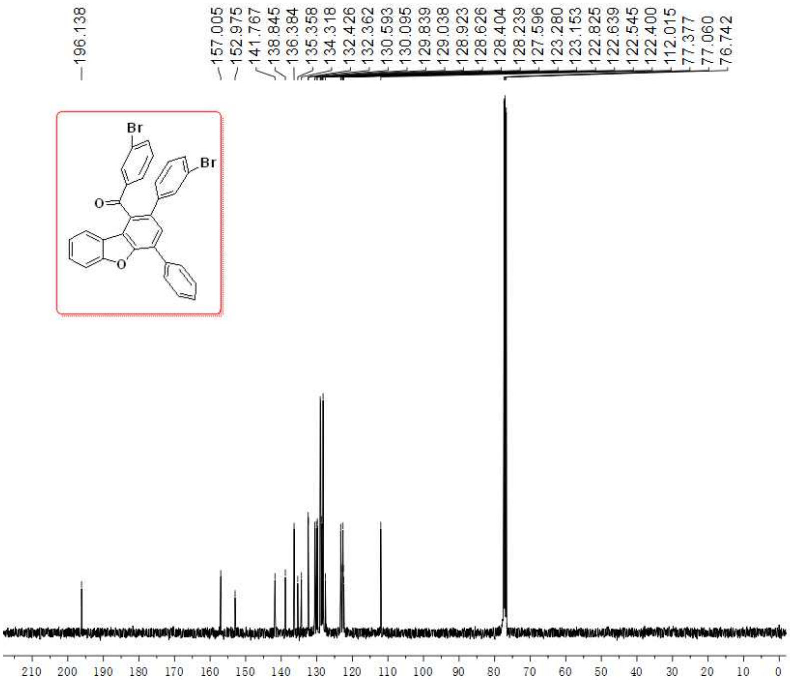

Parameters

	Parameter	值
1	Data File Name	C:/ Users/ liruxue/ Desktop/ 实验数据/ 2019-2-C/ 33/ pdata/ 1/ 1r
2	标题	2019-2-hxw-C. 33. 1. 1r
3	Comment	
4	Origin	Bruker BioSpin GmbH
5	Owner	root
6	Site	
7	Instrument	Avance
8	Author	
9	Solvent	CDCl3
10	Temperature	298.9
11	Pulse Sequence	zgpg30
12	Experiment	1D
13	Probe	Z163739_0032 (PI HR-400-S1-BBF/ H/ D-5.0-Z SP)
14	Number of Scans	1000
15	Receiver Gain	12.8
16	Relaxation Delay	2.0000
17	Pulse Width	10.0000
18	Presaturation Frequency	
19	Acquisition Time	1.3763
20	Acquisition Date	2019-08-27T14:57:37
21	Modification Date	2019-08-27T14:56:34
22	Class	
23	Spectrometer Frequency	100.61
24	Spectral Width	23809.5
25	Lowest Frequency	-1843.5
26	Nucleus	13C
27	Acquired Size	32768
28	Spectral Size	32768

Supplementary Figure 60 ^{13}C NMR Spectra of compound 3ak

(4-Bromophenyl)(2-(4-bromophenyl)-4-phenyldibenzo[*b,d*]furan-1-yl)methanone (Figure 3, compound 3al)

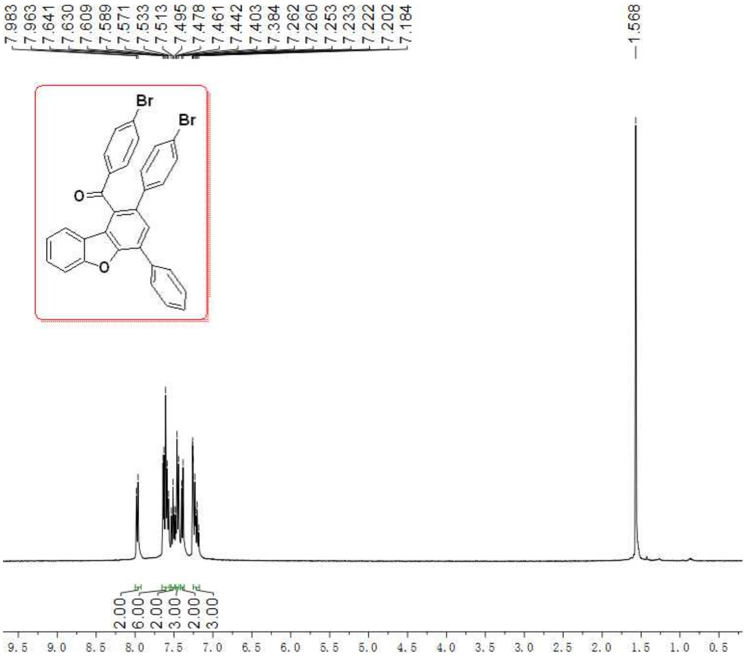

	Parameters	
	Parameter	值
1	Data File Name	C:/ Users/ liruxue/ Desktop/ 实验数据/ 2019-2/ 41/ pdata/ 1/ 1r
2	标题	2019-2-hxw-H.41.1.1r
3	Comment	
4	Origin	Bruker BioSpin GmbH
5	Owner	root
6	Site	
7	Instrument	Avance
8	Author	
9	Solvent	CDCl3
10	Temperature	297.1
11	Pulse Sequence	zg30
12	Experiment	1D
13	Probe	Z163739_0032 (PI HR-400-S1-BBF/ H/ D-5.0-Z SP)
14	Number of Scans	4
15	Receiver Gain	101.0
16	Relaxation Delay	1.0000
17	Pulse Width	10.0000
18	Presaturation Frequency	
19	Acquisition Time	3.9977
20	Acquisition Date	2019-07-11T18:47:35
21	Modification Date	2019-07-11T18:46:28
22	Class	
23	Spectrometer Frequency	400.13
24	Spectral Width	8196.7
25	Lowest Frequency	-1637.2
26	Nucleus	1H
27	Acquired Size	32768
28	Spectral Size	65536

Supplementary Figure 61 ^{1}H NMR Spectra of compound 3al

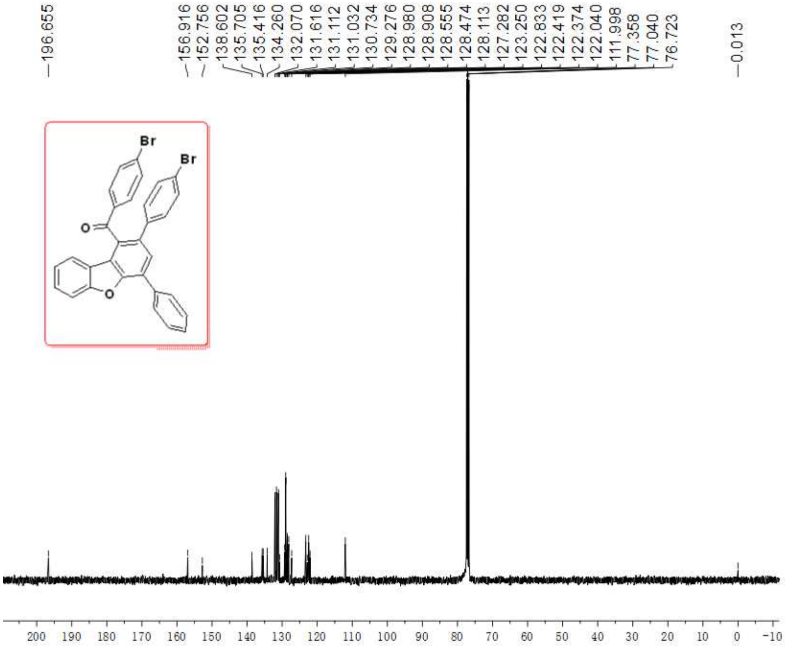

	Parameters	
	Parameter	值
1	Data File Name	C:/ Users/ liruxue/ Desktop/ 实验数据/ 2019-2-C/ 20/ pdata/ 1/ 1r
2	标题	2019-2-hxw-C.20.1.1r
3	Comment	
4	Origin	Bruker BioSpin GmbH
5	Owner	root
6	Site	
7	Instrument	Avance
8	Author	
9	Solvent	CDCl3
10	Temperature	297.8
11	Pulse Sequence	zgpg30
12	Experiment	1D
13	Probe	Z163739_0032 (PI HR-400-S1-BBF/ H/ D-5.0-Z SP)
14	Number of Scans	800
15	Receiver Gain	14.8
16	Relaxation Delay	2.0000
17	Pulse Width	10.0000
18	Presaturation Frequency	
19	Acquisition Time	1.3763
20	Acquisition Date	2019-07-13T07:04:15
21	Modification Date	2019-07-13T07:03:00
22	Class	
23	Spectrometer Frequency	100.61
24	Spectral Width	23809.5
25	Lowest Frequency	-1843.5
26	Nucleus	13C
27	Acquired Size	32768
28	Spectral Size	32768

Supplementary Figure 62 ^{13}C NMR Spectra of compound 3al

(4-Phenyl-2-(4-(trifluoromethyl)phenyl)dibenzo[*b,d*]furan-1-yl)(4-(trifluoromethyl)phenyl)methanone (Figure 3 and Figure 4, compound 3am)

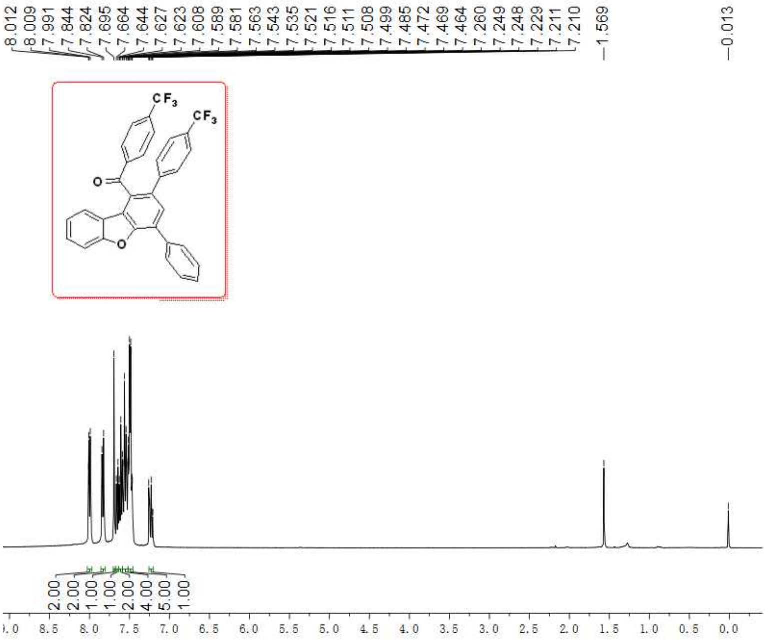

Parameters		
	Parameter	值
1	Data File Name	C:/ Users/ liruxue/ Desktop/ 实验数据/ 2019-2/ 81/ pdata/ 1/ 1r
2	标题	2019-2-hxw-H. 81. 1. 1r
3	Comment	
4	Origin	Bruker BioSpin GmbH
5	Owner	root
6	Site	
7	Instrument	Avance
8	Author	
9	Solvent	CDCl3
10	Temperature	297.3
11	Pulse Sequence	zg30
12	Experiment	1D
13	Probe	Z163739_0032 (PI HR-400-S1-BBF/ H/ D-5.0-Z SP)
14	Number of Scans	4
15	Receiver Gain	101.0
16	Relaxation Delay	1.0000
17	Pulse Width	10.0000
18	Presaturation Frequency	
19	Acquisition Time	3.9977
20	Acquisition Date	2019-08-30T21:18:19
21	Modification Date	2019-08-30T21:16:56
22	Class	
23	Spectrometer Frequency	400.13
24	Spectral Width	8196.7
25	Lowest Frequency	-1637.6
26	Nucleus	1H
27	Acquired Size	32768
28	Spectral Size	65536

Supplementary Figure 63 ^{1}H NMR Spectra of compound 3am

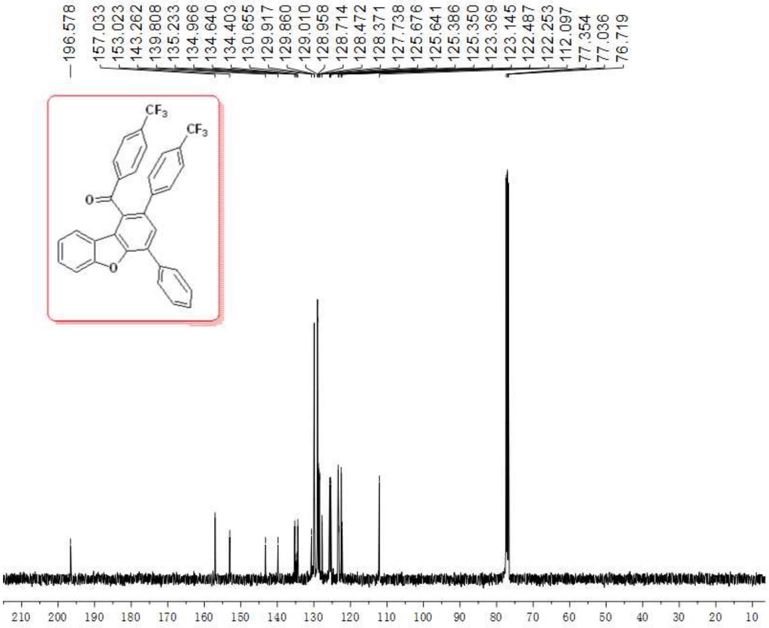

Parameters		
	Parameter	值
1	Data File Name	G:/ 实验数据/ 实验数据/ 86/ pdata/ 1/ 1r
2	标题	2019-2-hxw-H. 86. 1. 1r
3	Comment	
4	Origin	Bruker BioSpin GmbH
5	Owner	root
6	Site	
7	Instrument	Avance
8	Author	
9	Solvent	CDCl3
10	Temperature	302.0
11	Pulse Sequence	zgpg30
12	Experiment	1D
13	Probe	Z163739_0032 (PI HR-400-S1-BBF/ H/ D-5.0-Z SP)
14	Number of Scans	500
15	Receiver Gain	12.8
16	Relaxation Delay	2.0000
17	Pulse Width	10.0000
18	Presaturation Frequency	
19	Acquisition Time	1.3763
20	Acquisition Date	2019-09-03T18:37:05
21	Modification Date	2019-09-03T18:35:20
22	Class	
23	Spectrometer Frequency	100.61
24	Spectral Width	23809.5
25	Lowest Frequency	-1843.5
26	Nucleus	13C
27	Acquired Size	32768
28	Spectral Size	32768

Supplementary Figure 64 ^{13}C NMR Spectra of compound 3am

Naphthalen-1-yl(2-(naphthalen-1-yl)-4-phenyldibenzo[*b,d*]furan-1-yl)methanone (Figure 3, compound 3an)

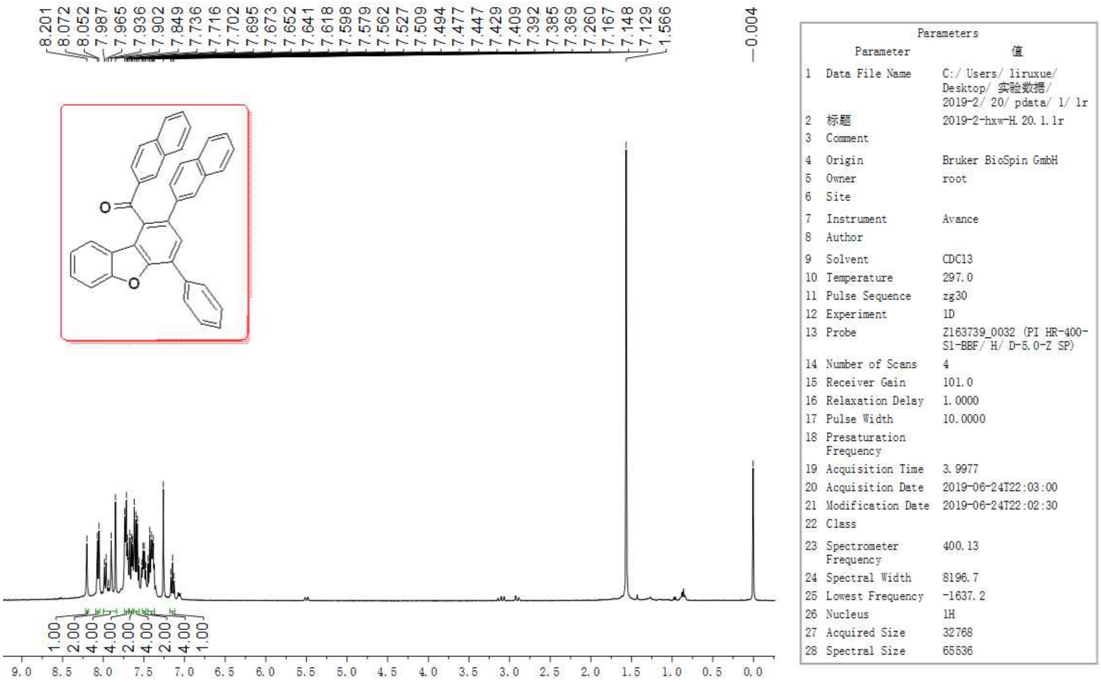

Supplementary Figure 65 ^{1}H NMR Spectra of compound 3an

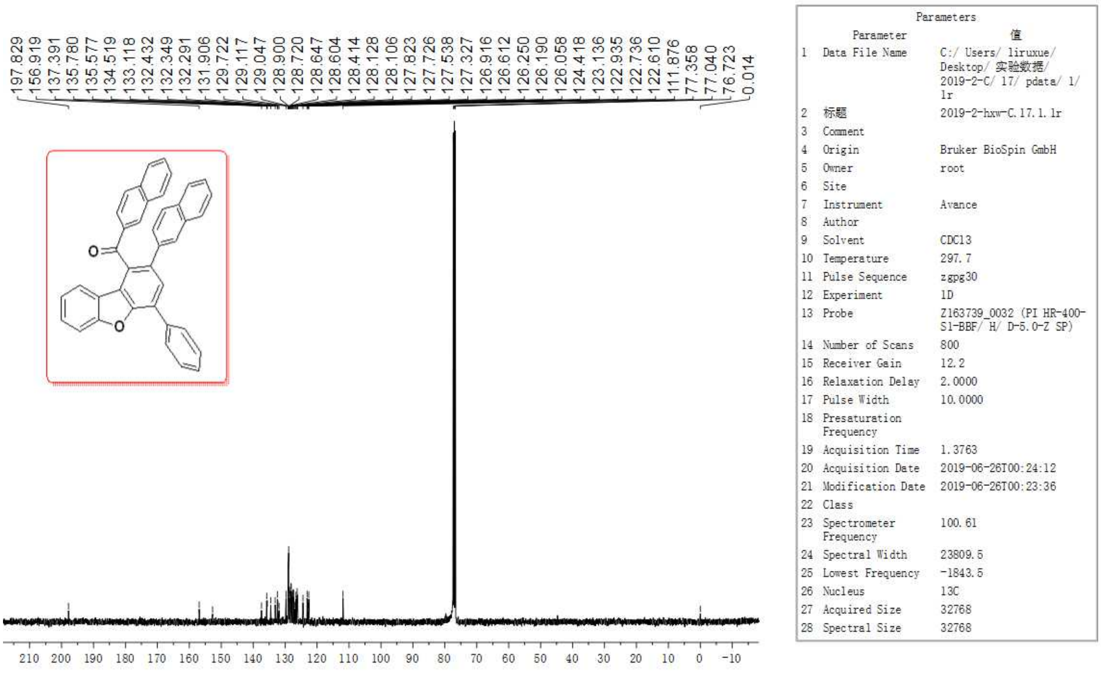

Supplementary Figure 66 ^{13}C NMR Spectra of compound 3an

(8-Bromo-2-(naphthalen-1-yl)-4-phenyldibenzo[*b,d*]furan-1-yl)(naphthalen-1-yl)methanone (Figure 3, compound 3ln)

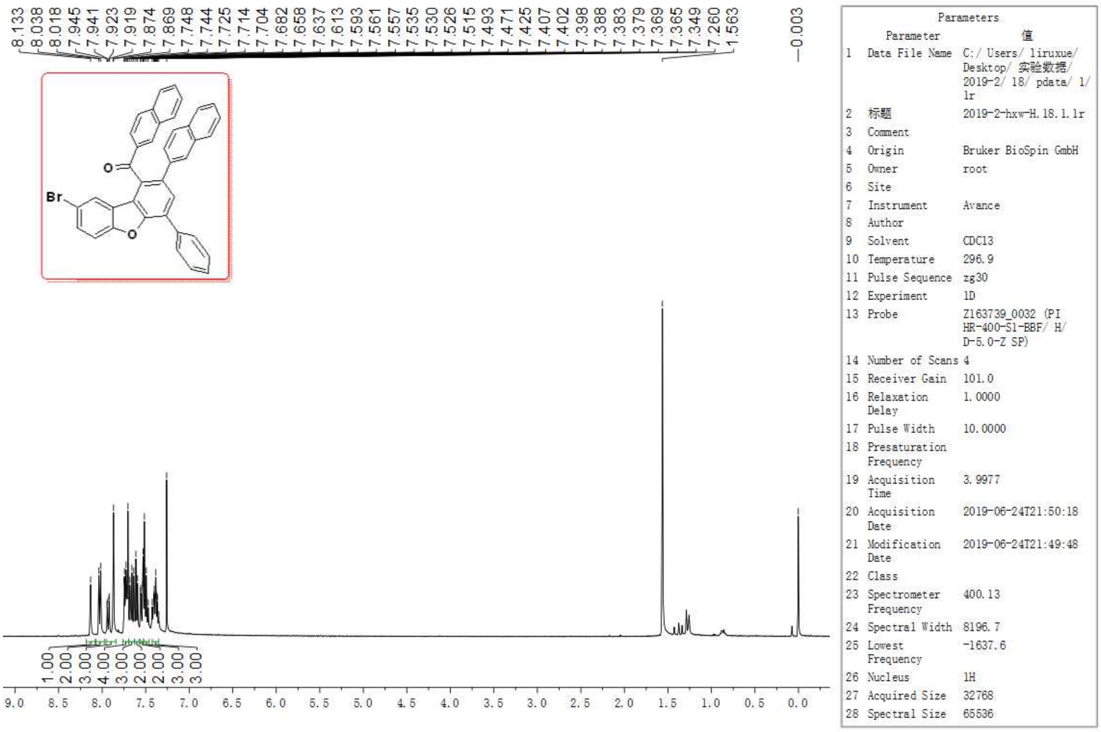

Supplementary Figure 67 ^{1}H NMR Spectra of compound 3ln

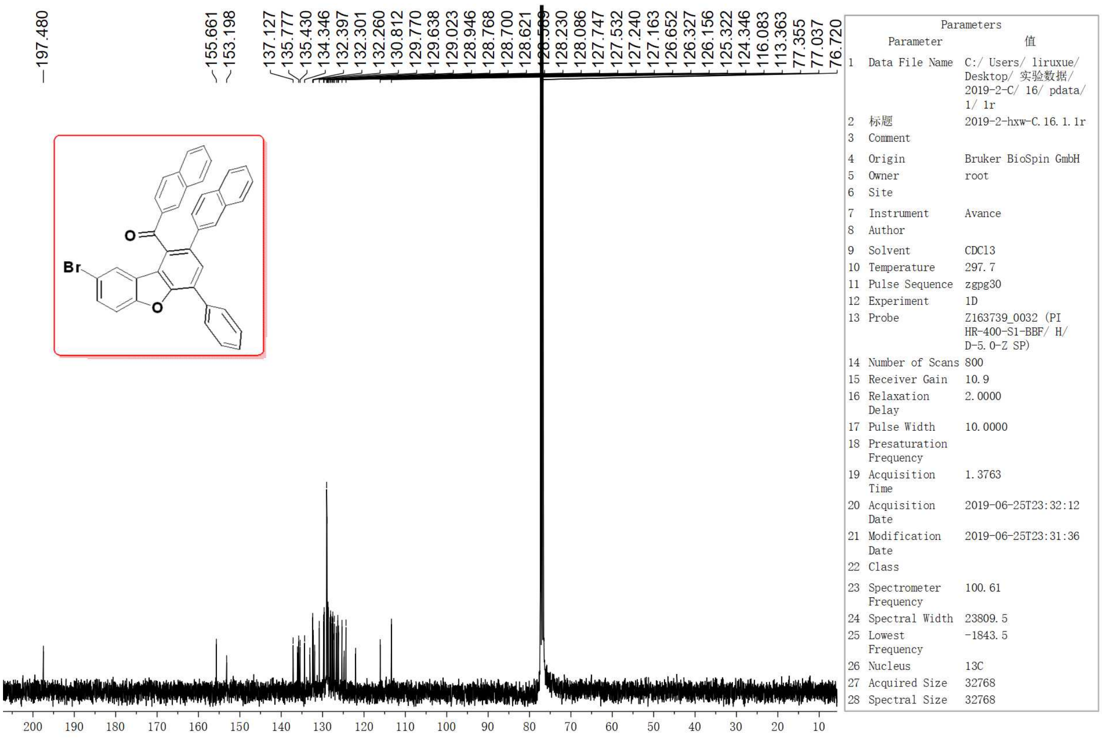

Supplementary Figure 68 ^{13}C NMR Spectra of compound 3ln

(8-Bromo-4-(4-methoxyphenyl)-2-(*p*-tolyl)dibenzo[*b,d*]furan-1-yl)(*p*-tolyl)methanone (Figure 3, compound 3nd)

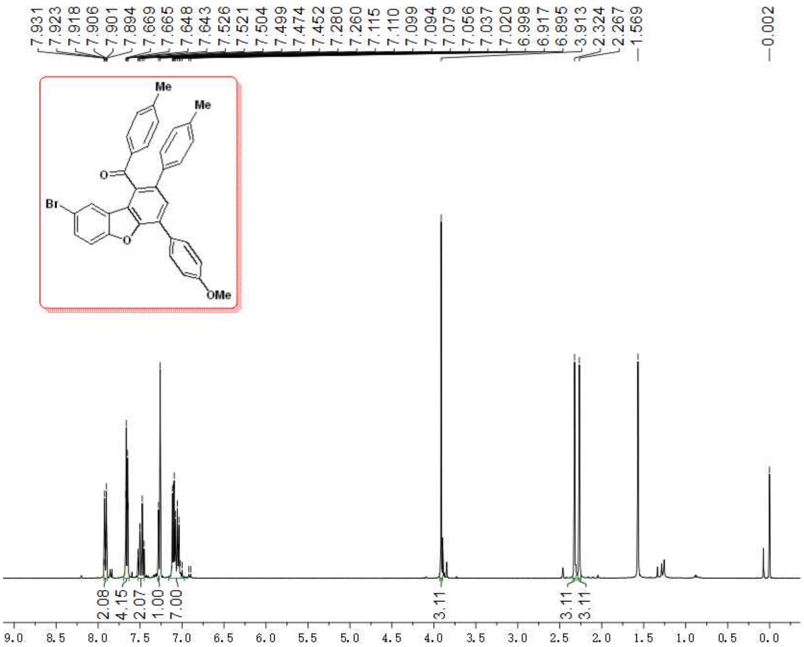

Parameters		
	Parameter	值
1	Data File Name	C:/ Users/ liruxue/ Desktop/ 实验数据/ 2019-1/ 124/ pdata/ 1/ 1r
2	标题	2019-1-hxw-H.124.1.1r
3	Comment	
4	Origin	Bruker BioSpin GmbH
5	Owner	nmrsu
6	Site	
7	Instrument	Avance
8	Author	
9	Solvent	CDCl3
10	Temperature	296.7
11	Pulse Sequence	zg30
12	Experiment	1D
13	Probe	Z163739_0032 (PI HR-400-S1-BBF/ H/ D-5.0-Z SP)
14	Number of Scans	4
15	Receiver Gain	101.0
16	Relaxation Delay	1.0000
17	Pulse Width	10.0000
18	Presaturation Frequency	
19	Acquisition Time	3.9977
20	Acquisition Date	2019-06-11T09:54:13
21	Modification Date	2019-06-11T09:53:36
22	Class	
23	Spectrometer Frequency	400.13
24	Spectral Width	8196.7
25	Lowest Frequency	-1637.6
26	Nucleus	1H
27	Acquired Size	32768
28	Spectral Size	65536

Supplementary Figure 69 ^{1}H NMR Spectra of compound 3nd

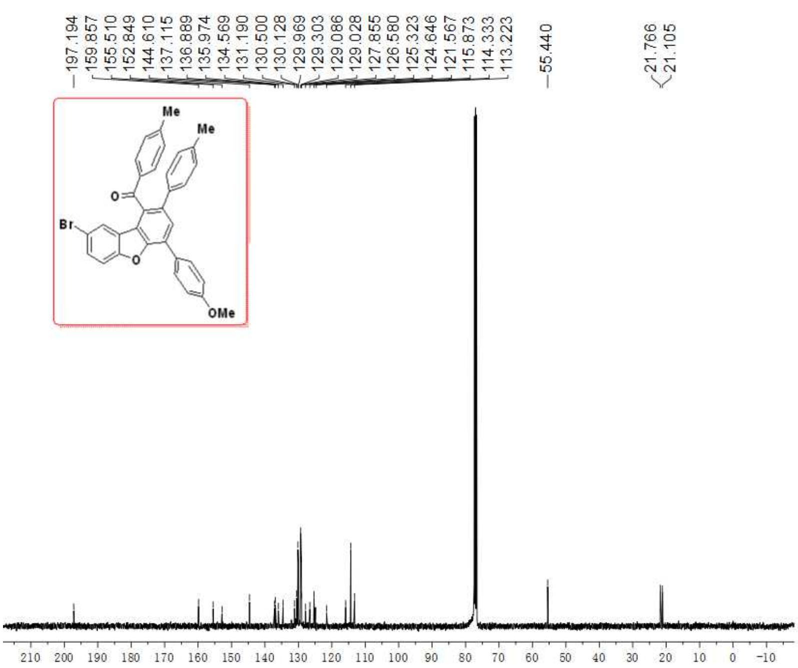

Parameters		
	Parameter	值
1	Data File Name	C:/ Users/ liruxue/ Desktop/ 实验数据/ 2019-2-C/ 1/ pdata/ 1/ 1r
2	标题	2019-2-hxw-C.1.1.1r
3	Comment	
4	Origin	Bruker BioSpin GmbH
5	Owner	nmrsu
6	Site	
7	Instrument	Avance
8	Author	
9	Solvent	CDCl3
10	Temperature	297.6
11	Pulse Sequence	zgpg30
12	Experiment	1D
13	Probe	Z163739_0032 (PI HR-400-S1-BBF/ H/ D-5.0-Z SP)
14	Number of Scans	1024
15	Receiver Gain	12.5
16	Relaxation Delay	2.0000
17	Pulse Width	10.0000
18	Presaturation Frequency	
19	Acquisition Time	1.3763
20	Acquisition Date	2019-06-13T01:45:08
21	Modification Date	2019-06-13T01:45:08
22	Class	
23	Spectrometer Frequency	100.61
24	Spectral Width	23809.5
25	Lowest Frequency	-1843.5
26	Nucleus	13C
27	Acquired Size	32768
28	Spectral Size	32768

Supplementary Figure 70 ^{13}C NMR Spectra of compound 3nd

(4-Phenyl-2-(thiophen-2-yl)dibenzo[b,d]furan-1-yl)(thiophen-2-yl)methanone (Figure 3, compound 3ap)

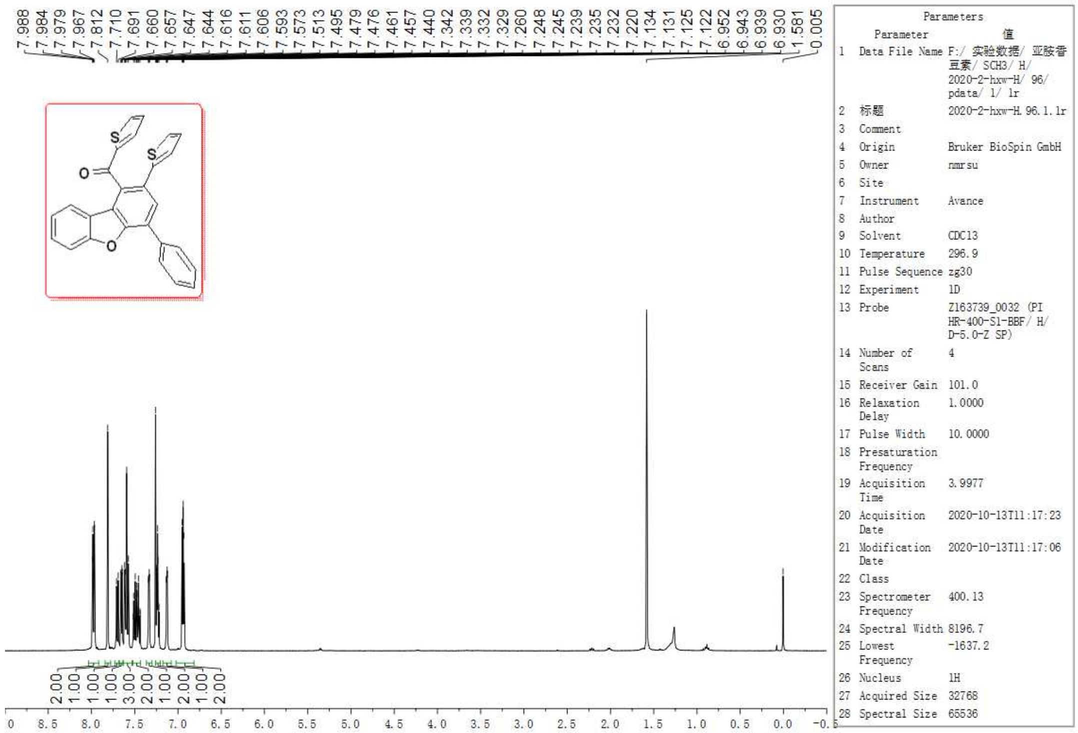

Supplementary Figure 71 ^{1}H NMR Spectra of compound 3ap

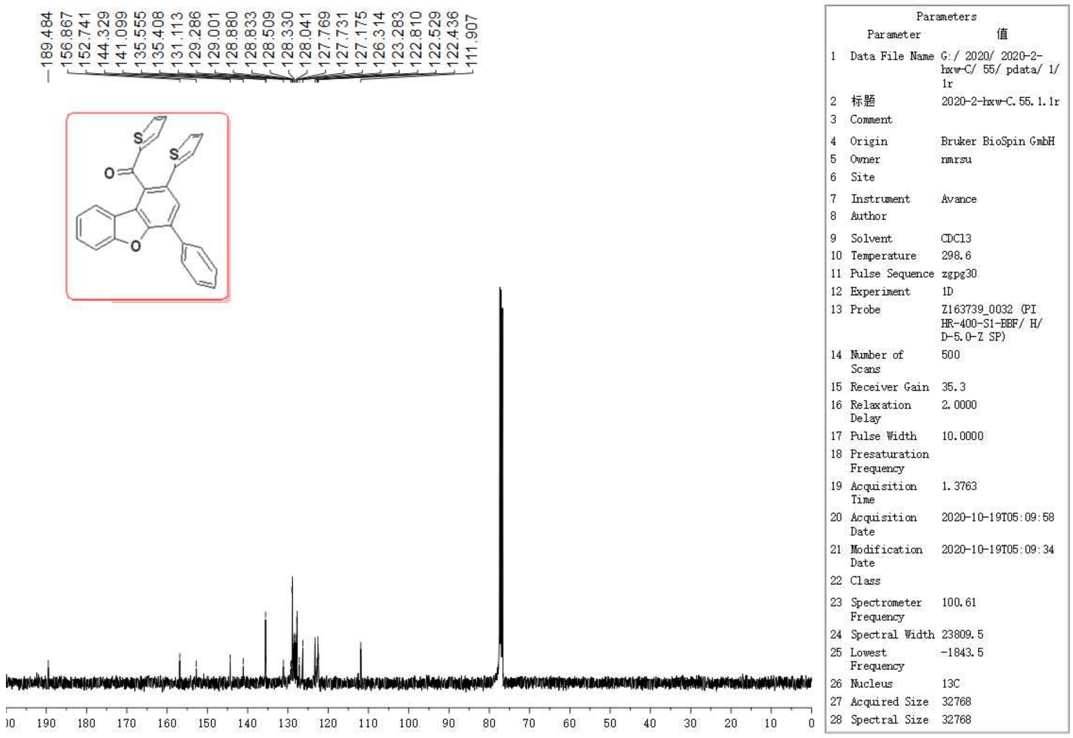

Supplementary Figure 72 ^{13}C NMR Spectra of compound 3ap

(2-(4-Methoxyphenyl)-4-phenyldibenzo[*b,d*]furan-1-yl)(4-(trifluoromethyl)phenyl)methanone (Figure 4, compound 3afm)

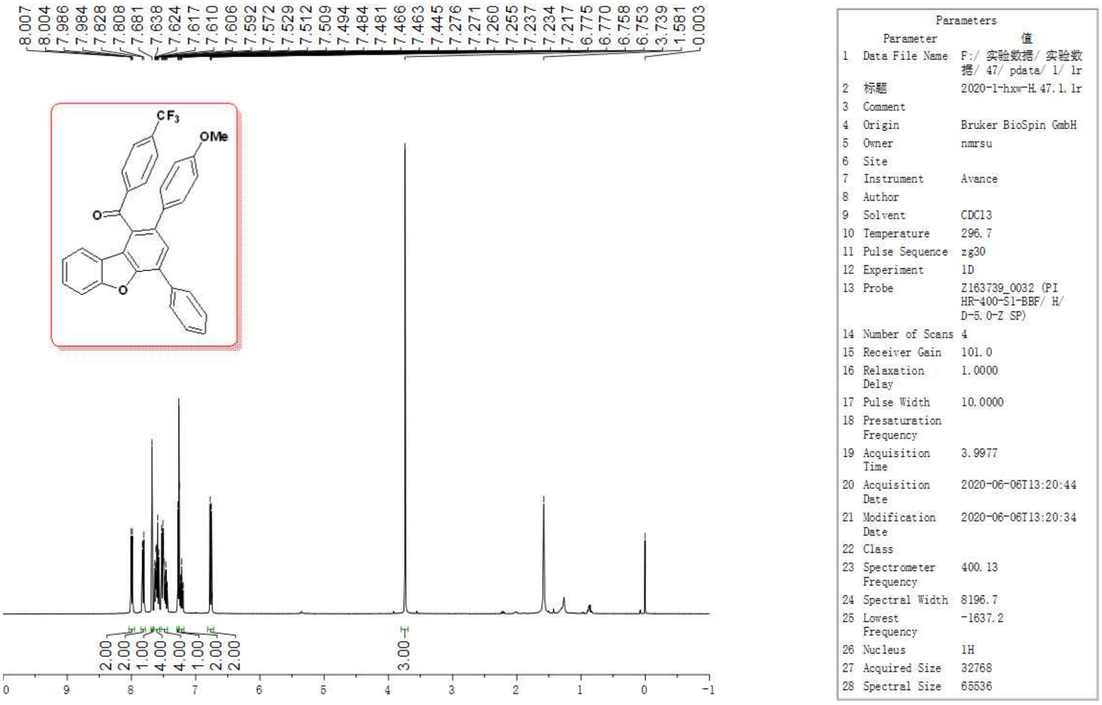

Supplementary Figure 73 ^{1}H NMR Spectra of compound 3afm

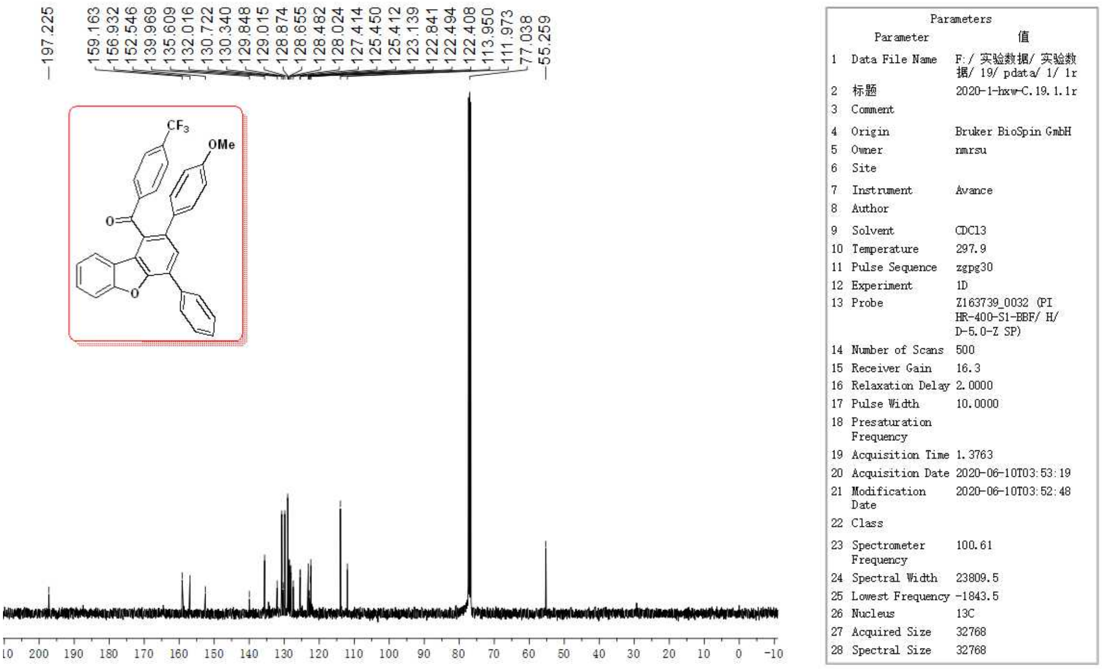

Supplementary Figure 74 ^{13}C NMR Spectra of compound 3afm

6,12-Diphenyl-12-(*p*-tolyl)-12*H*-fluoreno[2,1-*b*]benzofuran (Figure 5, compound 4aa)

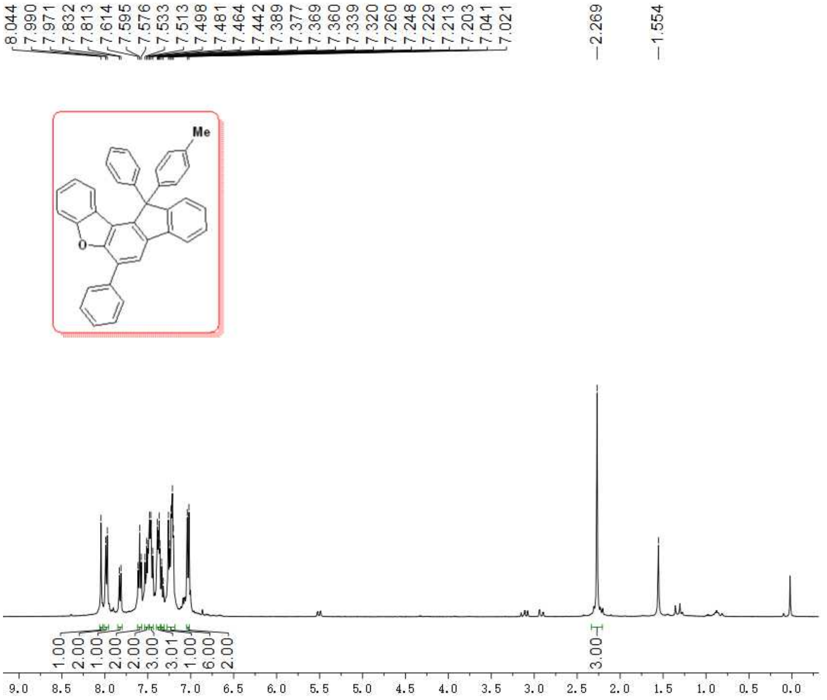

Parameters

	Parameter	值
1	Data File Name	F:/ 实验数据/ 实验数据/ 2019-2/ 161/ pdata/ 1/ 1r
2	标题	2019-2-hxw-H.161.1.1r
3	Comment	
4	Origin	Bruker BioSpin GmbH
5	Owner	nmrsu
6	Site	
7	Instrument	Avance
8	Author	
9	Solvent	CDCl3
10	Temperature	296.5
11	Pulse Sequence	zg30
12	Experiment	1D
13	Probe	Z163739_0032 (PI HR-400-S1-BBF/ H/ D-5.0-Z SP)
14	Number of Scans	4
15	Receiver Gain	101.0
16	Relaxation Delay	1.0000
17	Pulse Width	10.0000
18	Presaturation Frequency	
19	Acquisition Time	3.9977
20	Acquisition Date	2019-12-17T00:23:02
21	Modification Date	2019-12-17T00:23:00
22	Class	
23	Spectrometer Frequency	400.13
24	Spectral Width	8196.7
25	Lowest Frequency	-1637.2
26	Nucleus	1H
27	Acquired Size	32768
28	Spectral Size	65536

Supplementary Figure 75 ^{1}H NMR Spectra of compound 4aa

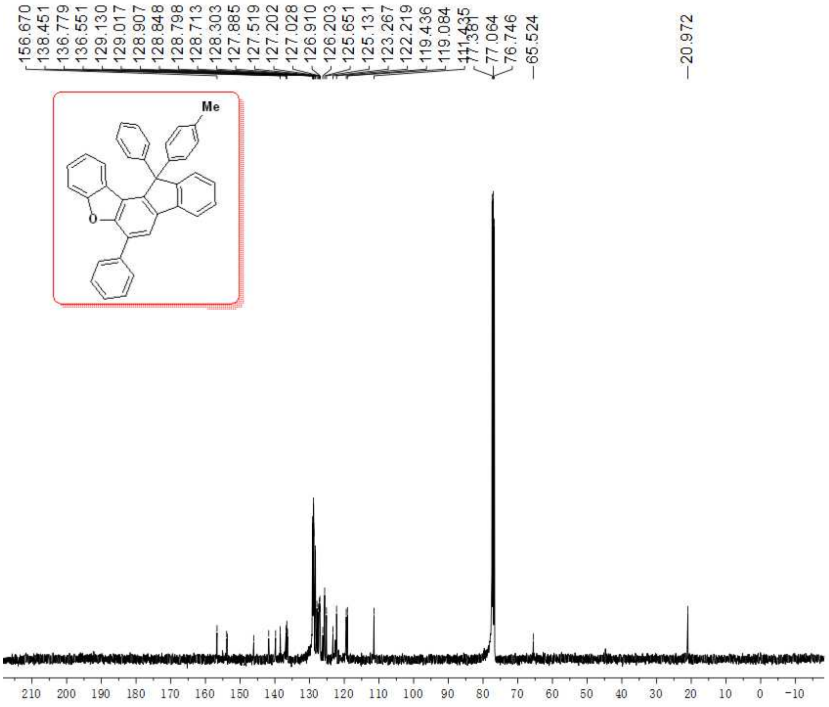

Parameters

	Parameter	值
1	Data File Name	F:/ 实验数据/ 实验数据/ 2019-2-C/ 70/ pdata/ 1/ 1r
2	标题	2019-2-hxw-C.70.1.1r
3	Comment	
4	Origin	Bruker BioSpin GmbH
5	Owner	nmrsu
6	Site	
7	Instrument	Avance
8	Author	
9	Solvent	CDCl3
10	Temperature	295.9
11	Pulse Sequence	zgpg30
12	Experiment	1D
13	Probe	Z163739_0032 (PI HR-400-S1-BBF/ H/ D-5.0-Z SP)
14	Number of Scans	1200
15	Receiver Gain	14.0
16	Relaxation Delay	2.0000
17	Pulse Width	10.0000
18	Presaturation Frequency	
19	Acquisition Time	1.3763
20	Acquisition Date	2019-12-18T14:56:53
21	Modification Date	2019-12-18T14:56:42
22	Class	
23	Spectrometer Frequency	100.61
24	Spectral Width	23809.5
25	Lowest Frequency	-1843.5
26	Nucleus	13C
27	Acquired Size	32768
28	Spectral Size	32768

Supplementary Figure 76 ^{13}C NMR Spectra of compound 4aa

6-(4-Methoxyphenyl)-12-phenyl-12-(*p*-tolyl)-12*H*-fluoreno[2,1-*b*]benzofuran (Figure 5, compound 4ca)

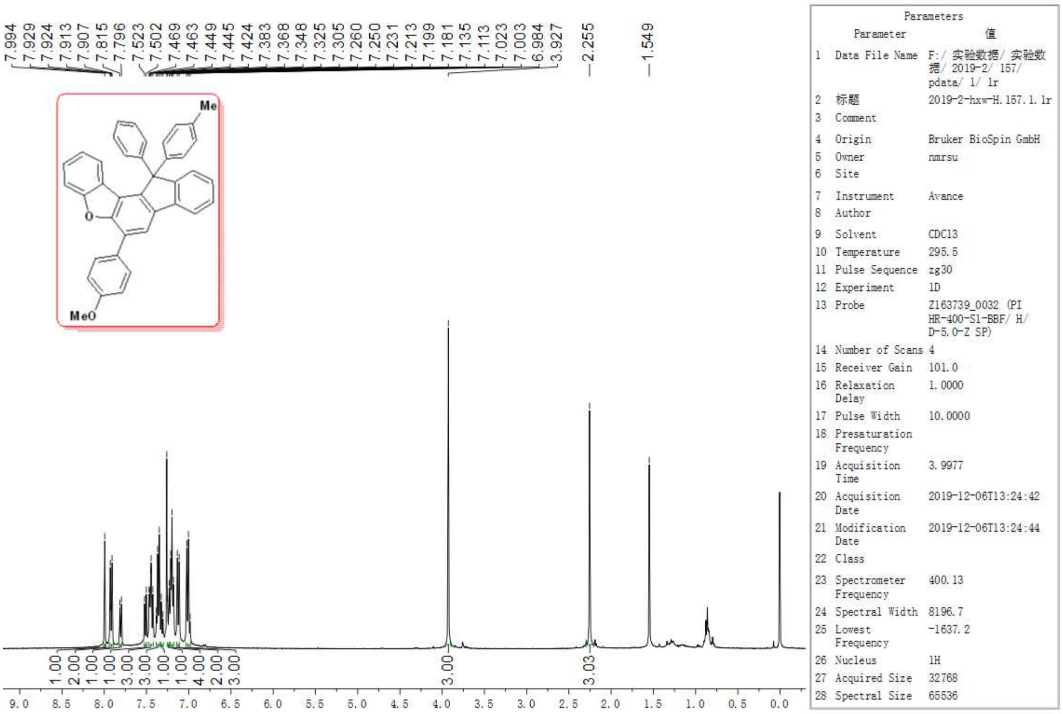

Supplementary Figure 77 ^{1}H NMR Spectra of compound 4ca

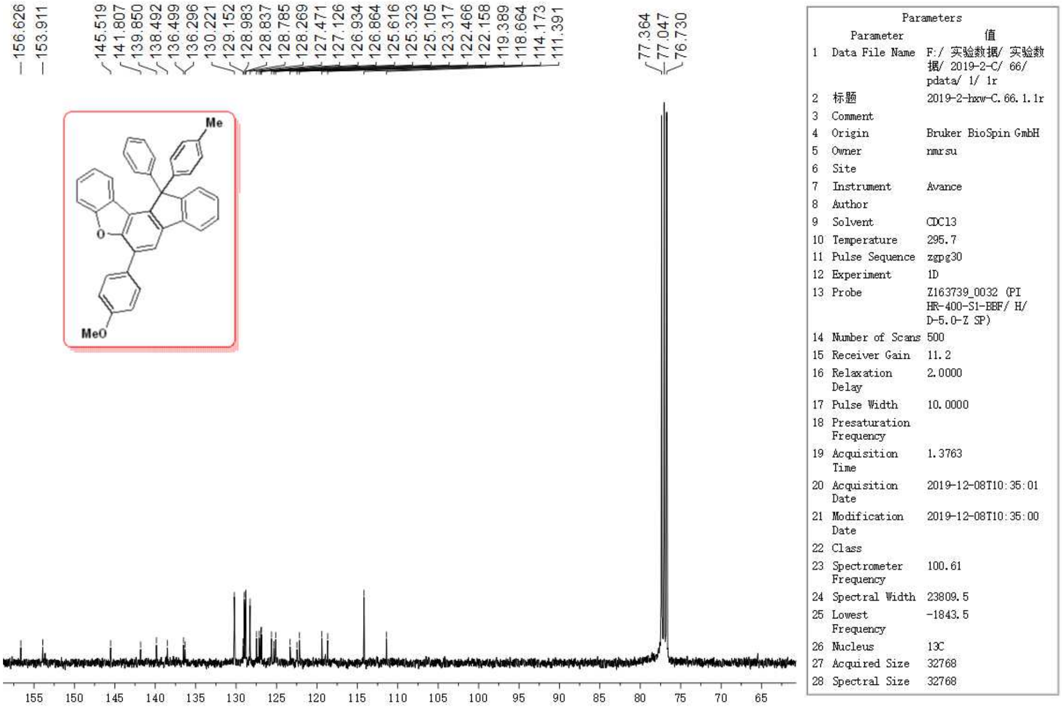

Supplementary Figure 78 ^{13}C NMR Spectra of compound 4ca

2-Chloro-6-(4-methoxyphenyl)-12-phenyl-12-(*p*-tolyl)-12*H*-fluoreno[2,1-*b*]benzofuran (Figure 5, compound 4ia)

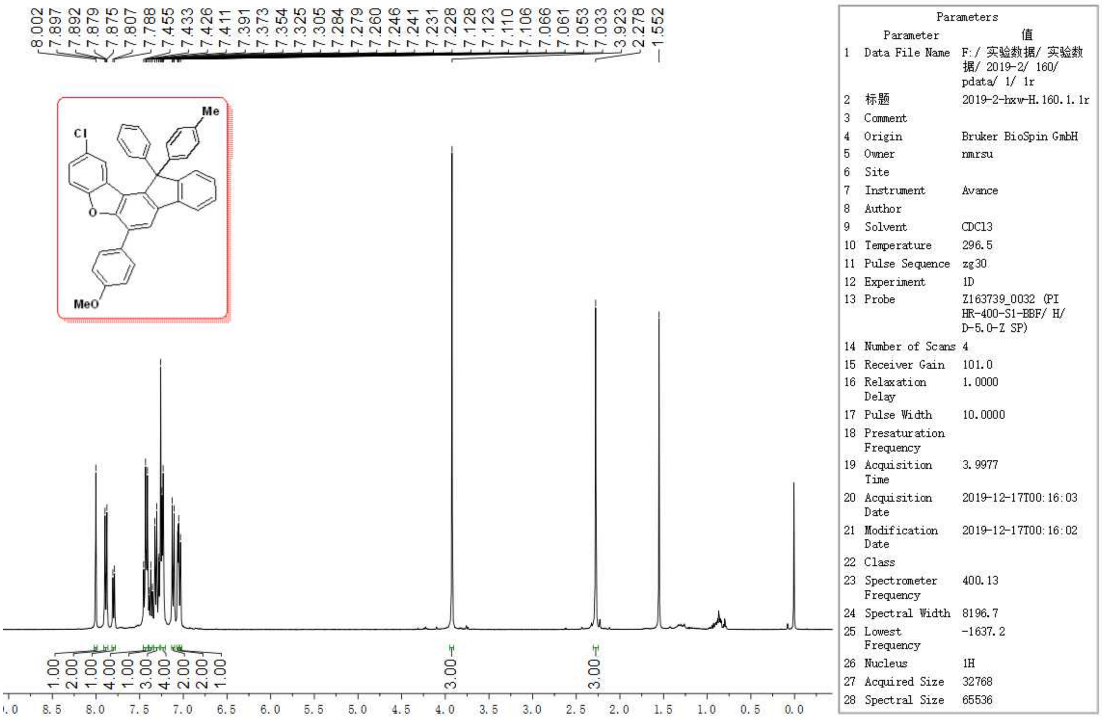

Supplementary Figure 79 ^{1}H **NMR Spectra of compound 4ia**

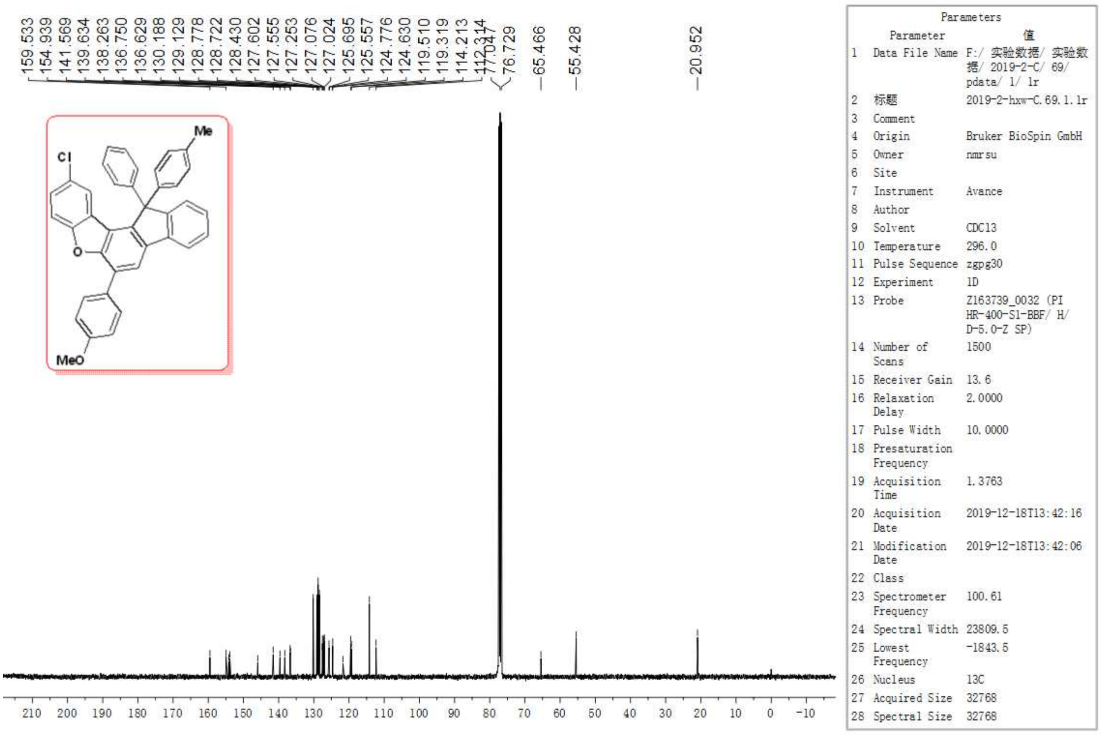

Supplementary Figure 80 ^{13}C **NMR Spectra of compound 4ia**

6,12-Diphenyl-12*H*-fluoreno[2,1-*b*]benzofuran-12-ol (Figure 5, compound 5aa)

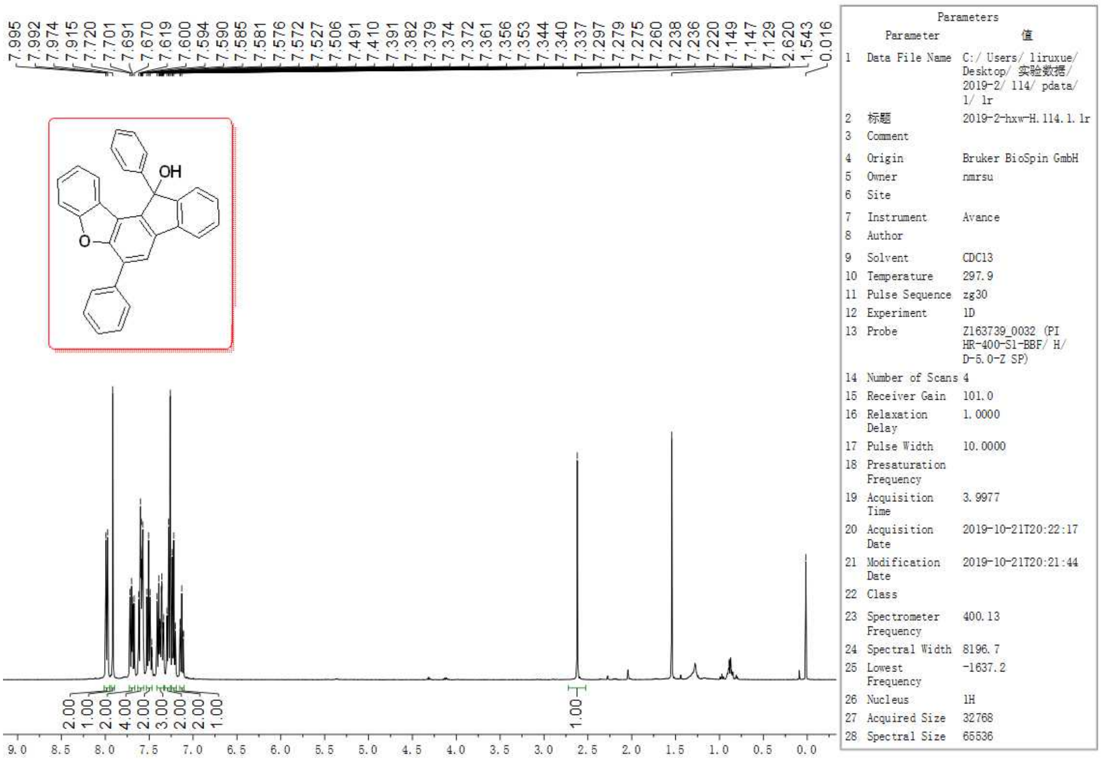

Supplementary Figure 81 ^{1}H NMR Spectra of compound 5aa

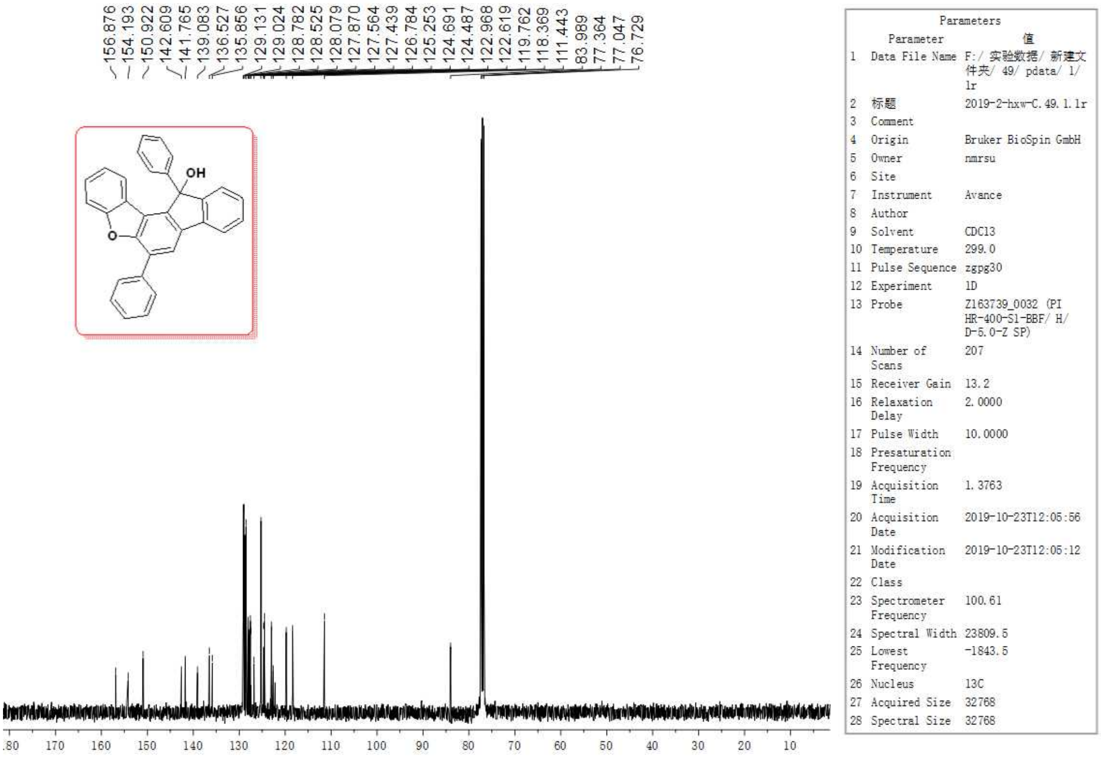

Supplementary Figure 82 ^{13}C NMR Spectra of compound 5aa

9-Methoxy-12-(3-methoxyphenyl)-6-phenyl-12*H*-fluoreno[2,1-*b*]benzofuran-12-ol (Figure 5, compound 5af)

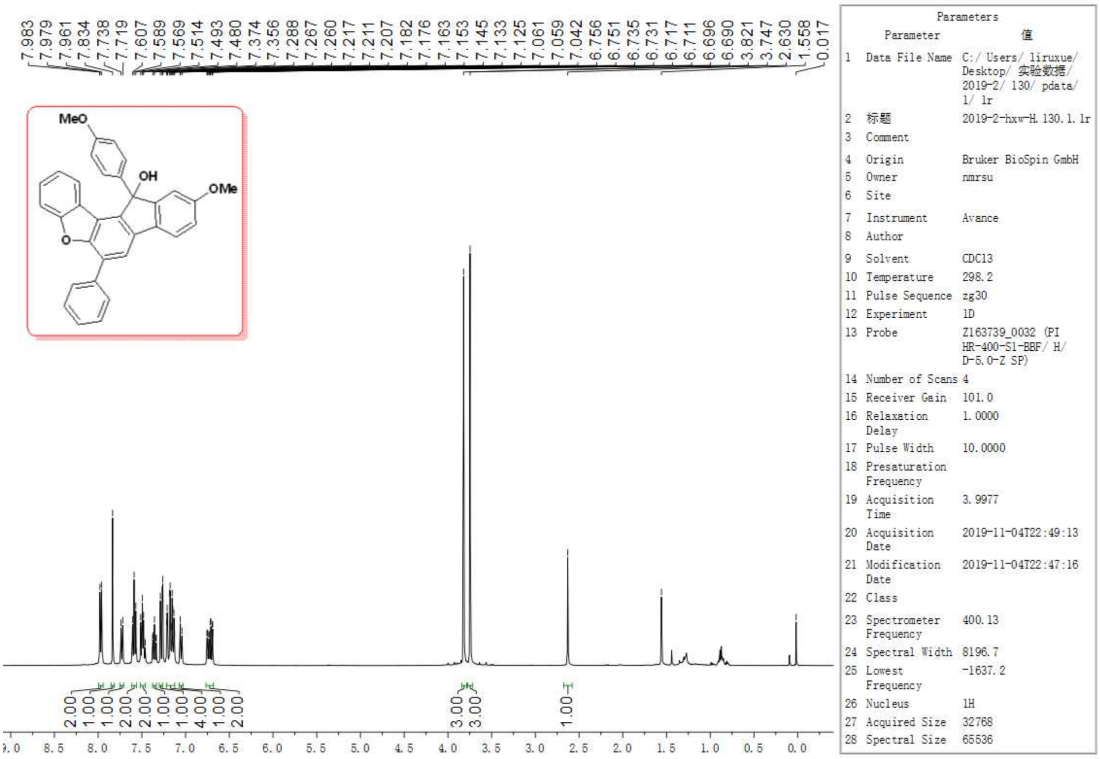

Supplementary Figure 83 ¹H NMR Spectra of compound 5af

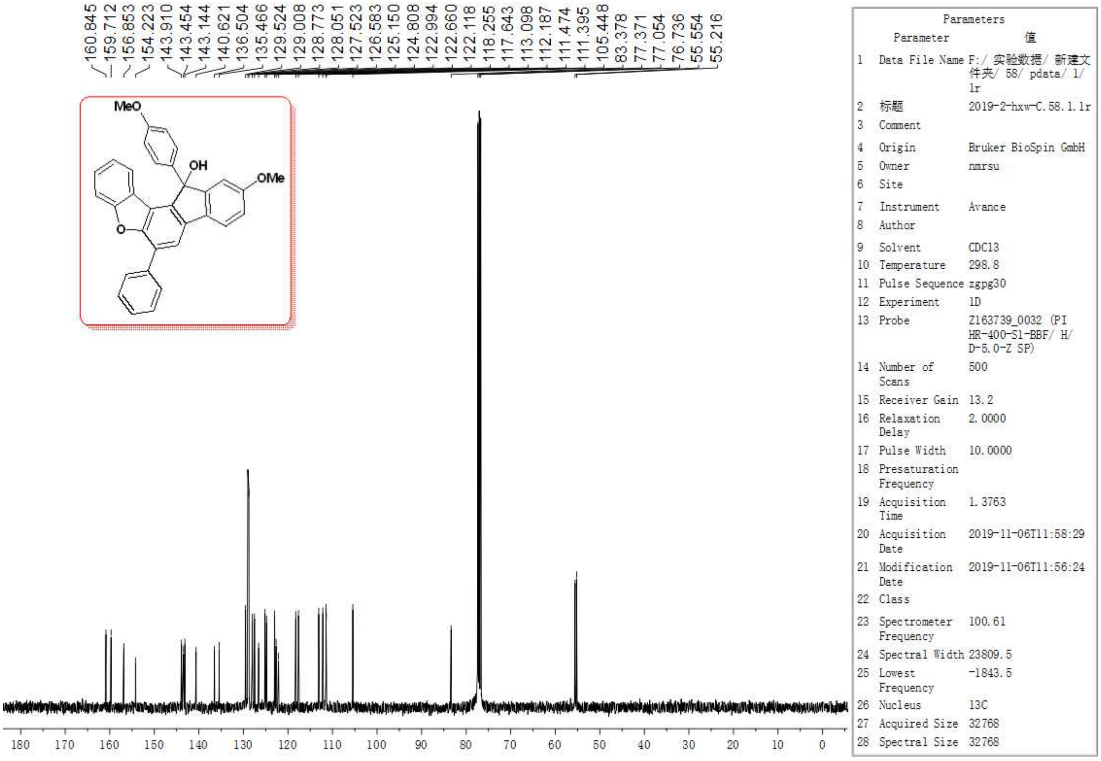

Supplementary Figure 84 ¹³C NMR Spectra of compound 5af

2-Phenyl-6-(*p*-tolyl)-12*H*-fluoreno[2,1-*b*]benzofuran-12-ol (Figure 5, compound 5ba)

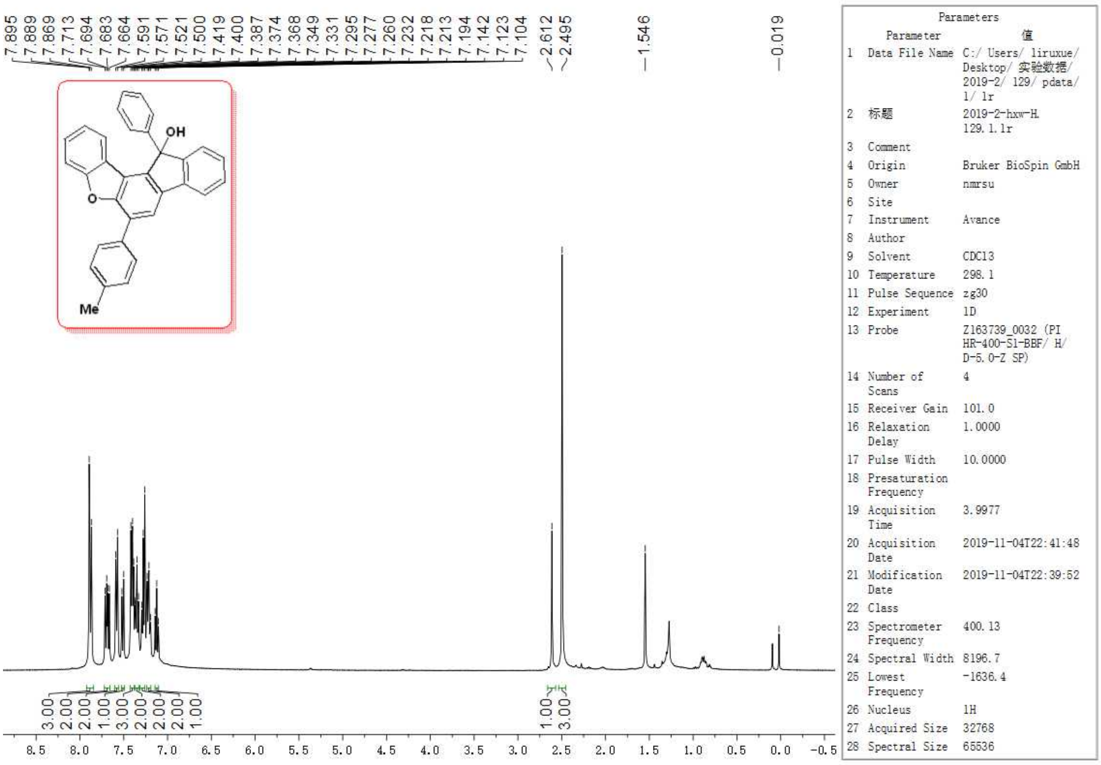

Supplementary Figure 85 ^{1}H NMR Spectra of compound 5ba

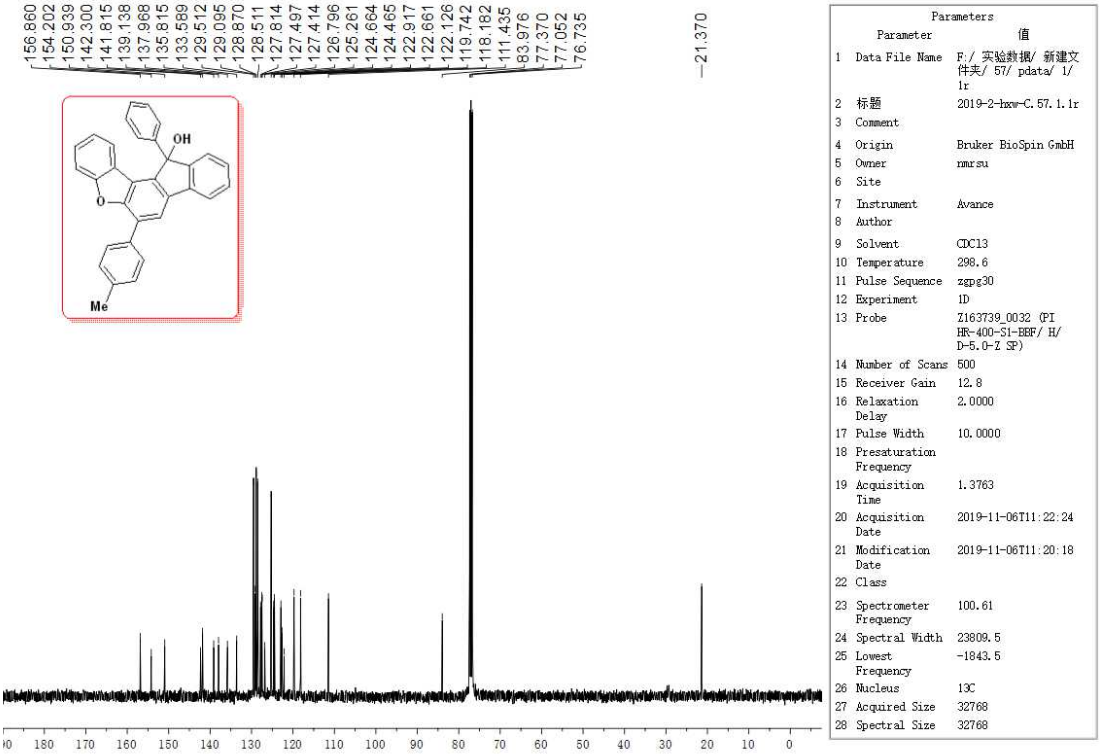

Supplementary Figure 86 ^{13}C NMR Spectra of compound 5ba

4-Bromo-12-phenyl-6-(*p*-tolyl)-12*H*-fluoreno[2,1-*b*]benzofuran-12-ol (Figure 5, compound 5oa)

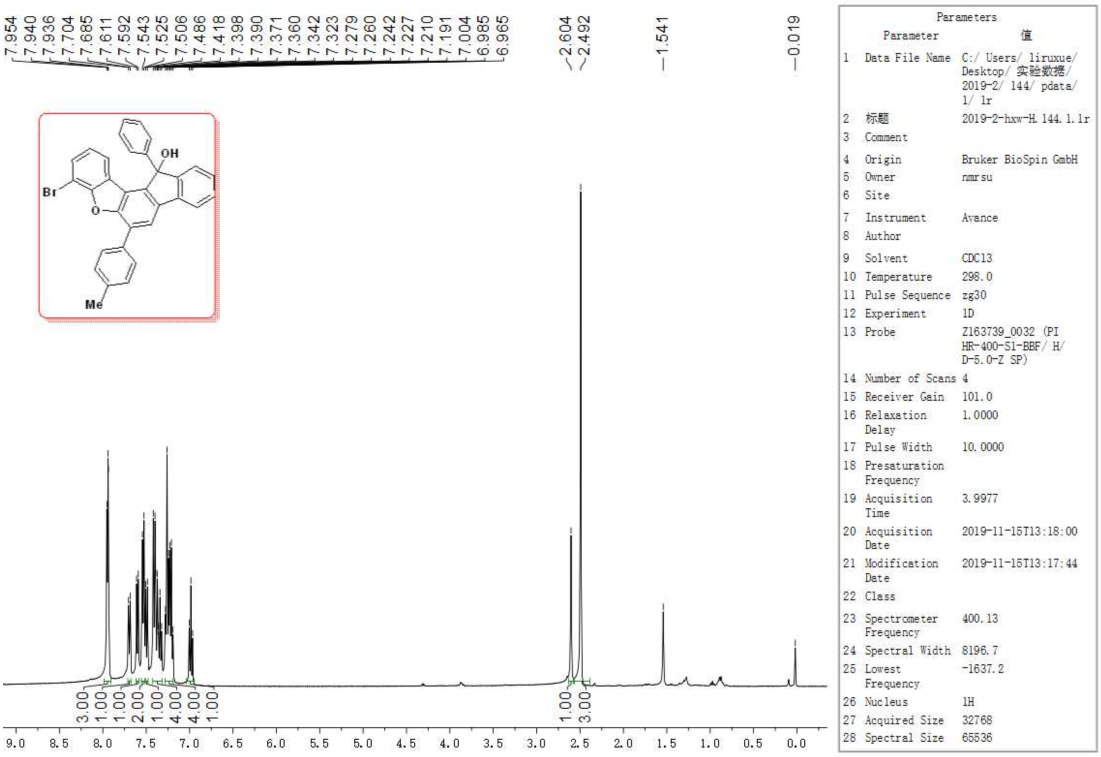

Supplementary Figure 87 ^{1}H NMR Spectra of compound 5oa

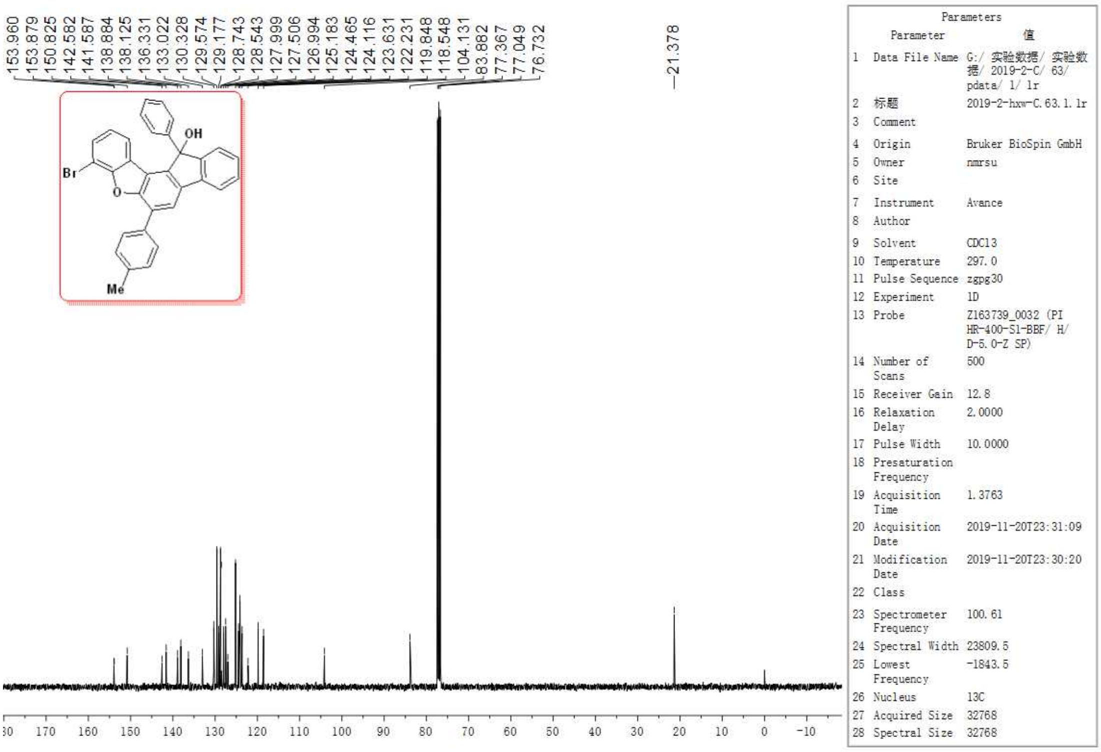

Supplementary Figure 88 ^{13}C NMR Spectra of compound 5oa

13.GC-MS spectra for mechanistic investigations

Intermediate *o*-AQM

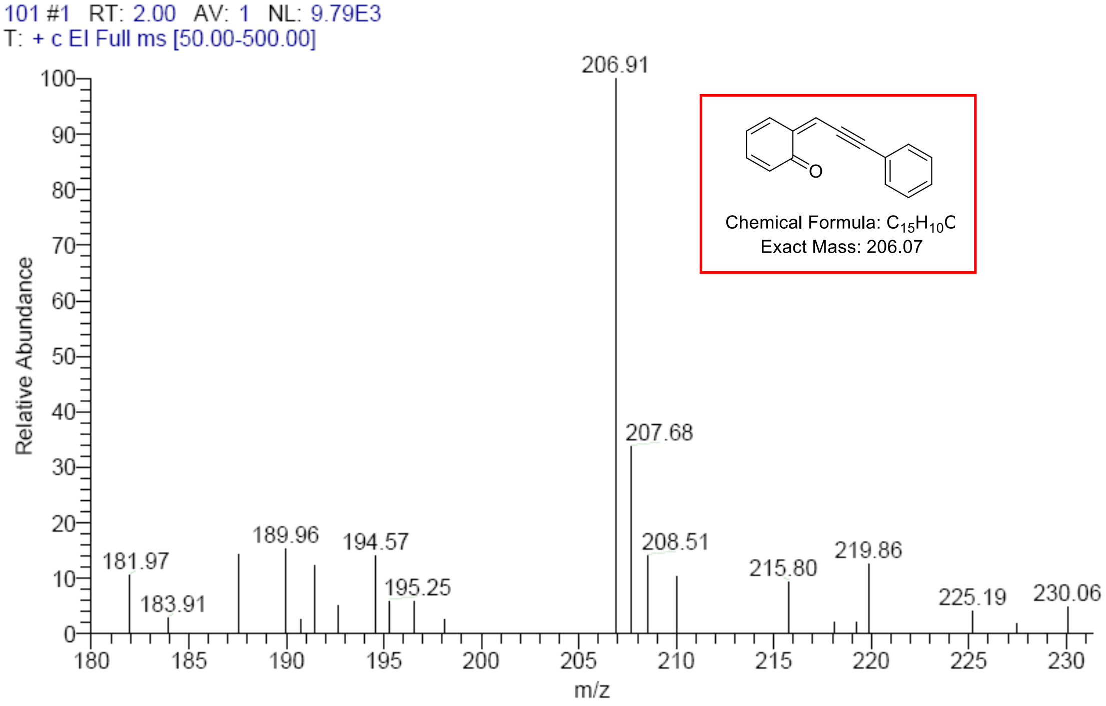

Supplementary Figure 89 GC-MS Spectra of possibly intermediate *o*-AQM

Intermediate A

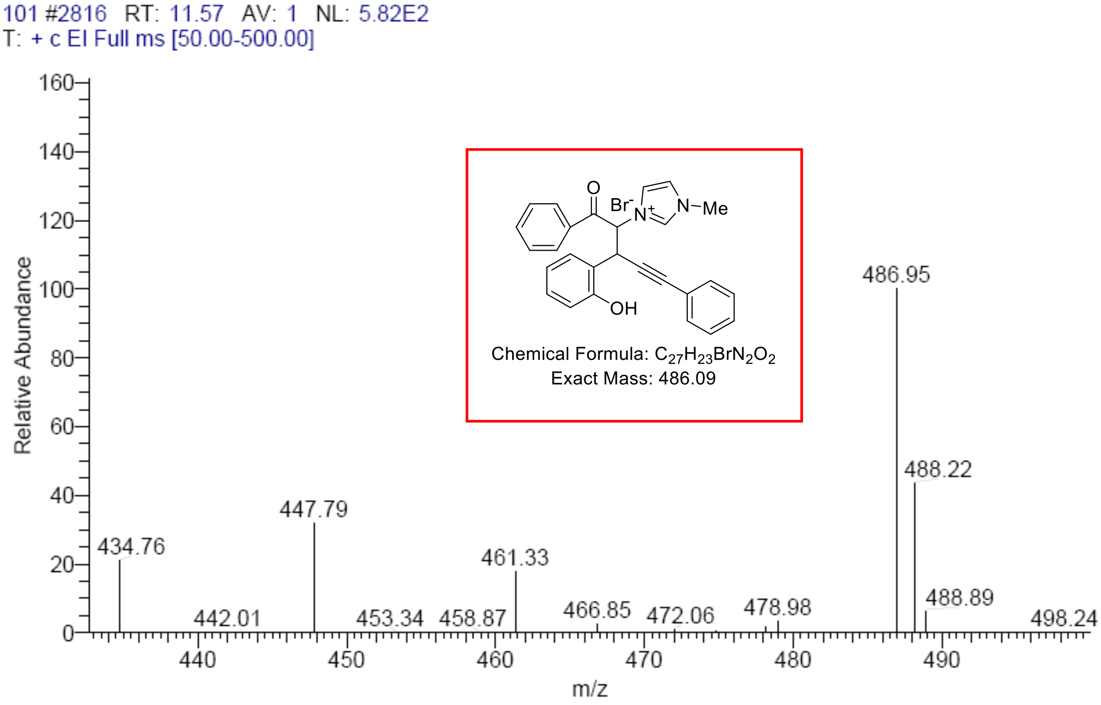

Supplementary Figure 90 GC-MS Spectra of possibly intermediate A

Intermediate B

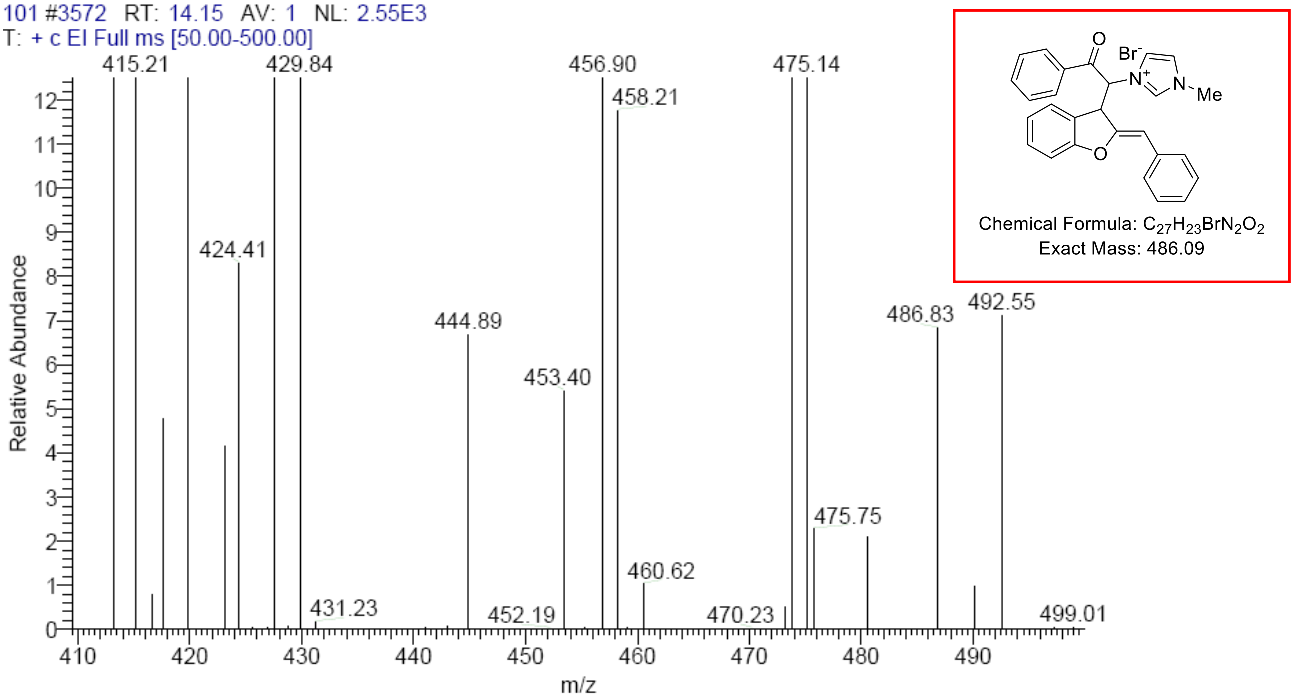

Supplementary Figure 91 GC-MS Spectra of possibly intermediate B

Intermediate C

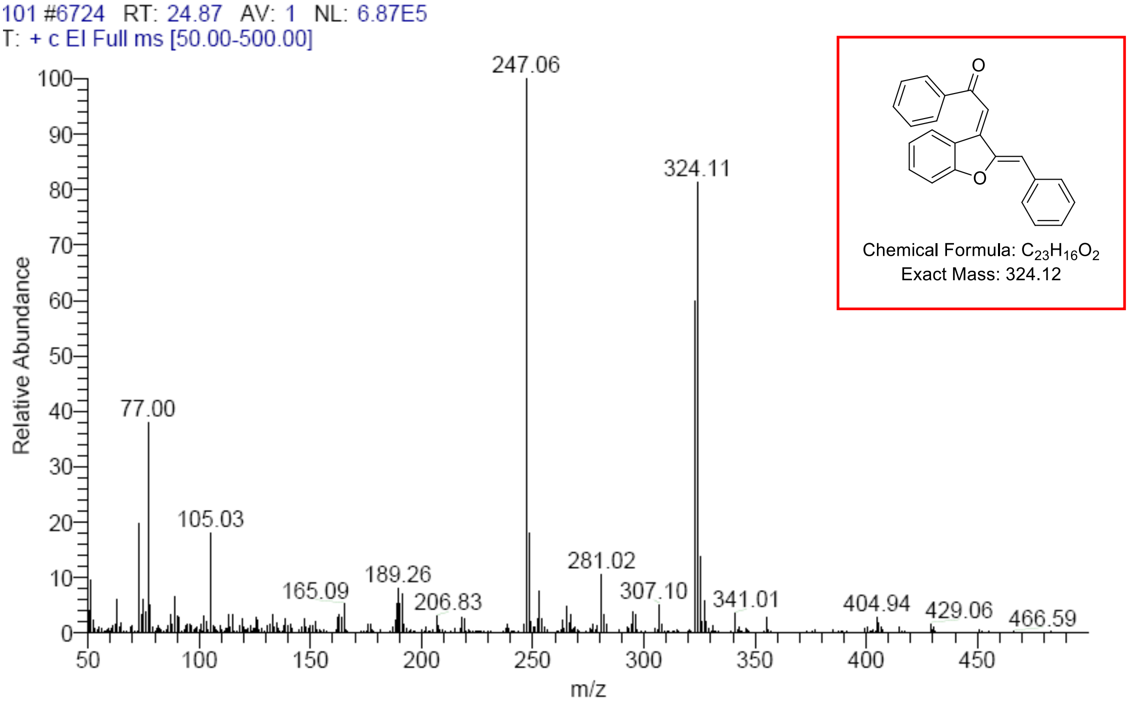

Supplementary Figure 92 GC-MS Spectra of possibly intermediate C

Intermediate E

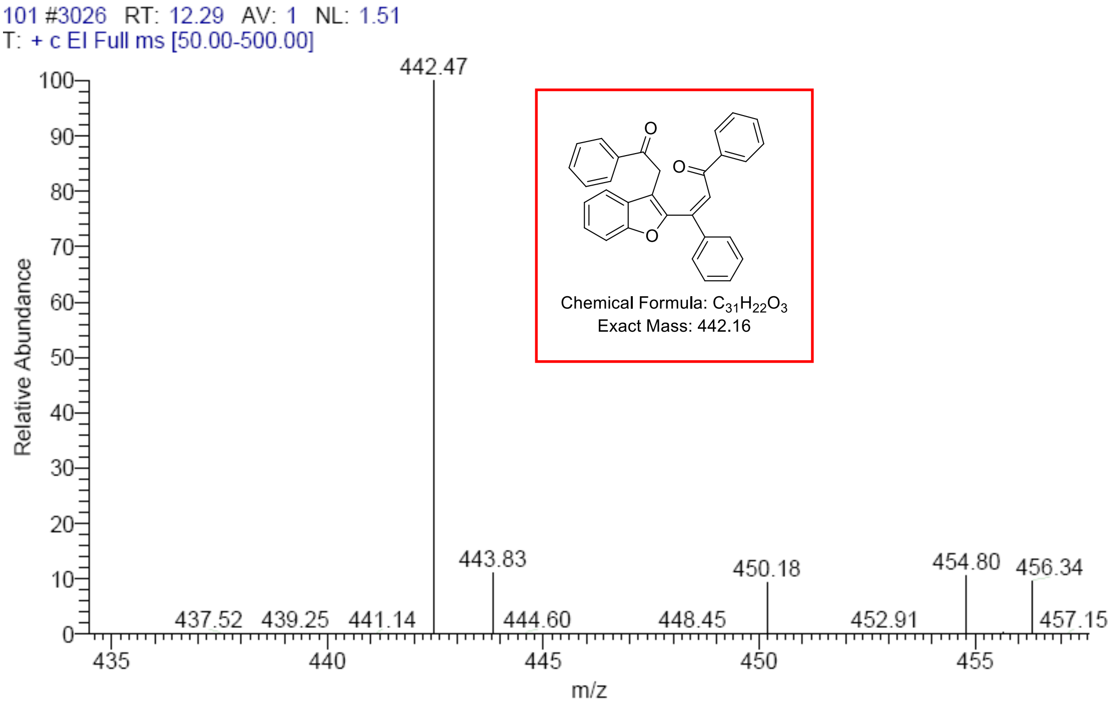

Supplementary Figure 93 GC-MS Spectra of possibly intermediate E

Intermediate F

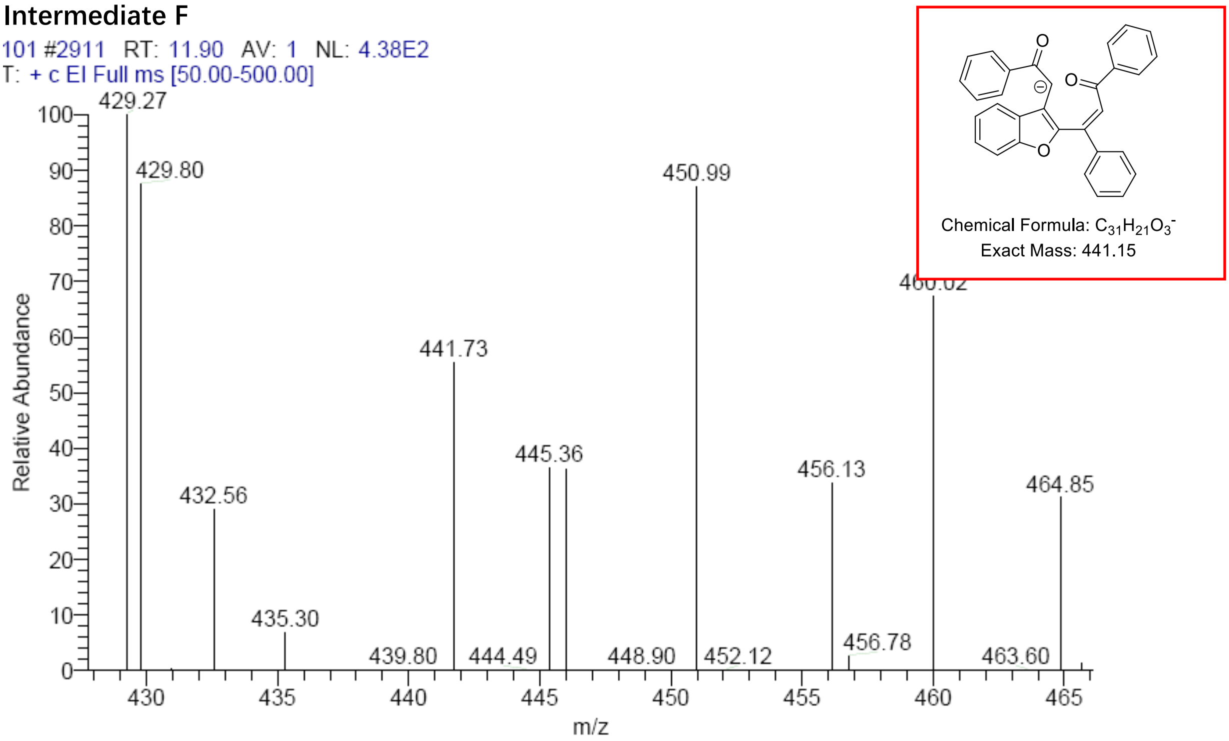

Supplementary Figure 94 GC-MS Spectra of possibly intermediate F

Intermediate G

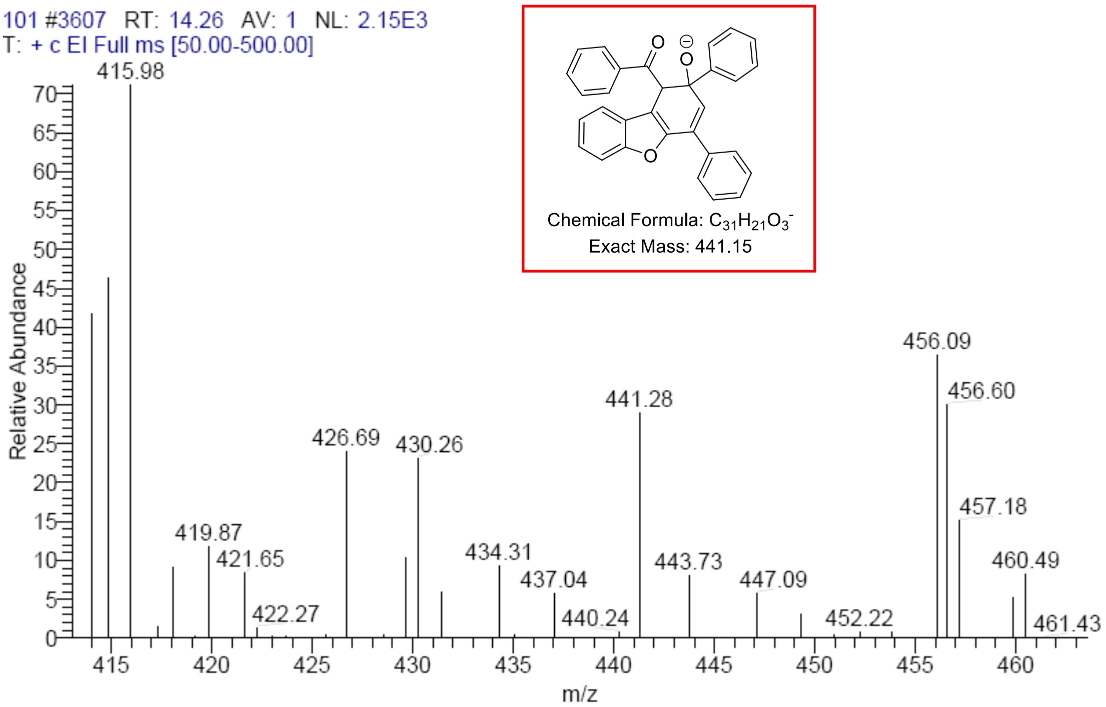

Supplementary Figure 95 GC-MS Spectra of possibly intermediate G

14.Supplementary References

1 Armarego, W. L. F. & Perrin, D. D. *Purification of Laboratory Chemicals*, 4th ed. Butterworth-Heinemann: Oxford, UK (1996).

2 He, X. et al. A ZnI_2-catalyzed regioselective cascade 1,4-conjugate addition/5-exo-dig annulation pathway for one-pot access to heterobiaryl frameworks. *Chem. Commun.* **55**, 15069-15072 (2019).
