# Supplementary material for: A cascade double 1,4-addition/intramolecular annulation strategy for expeditious assembly of unsymmetrical dibenzofurans
Source: Commun Chem. 2021 Mar 25;4:42. doi: 10.1038/s42004-021-00478-2 (PMC9814151; doi:10.1038/s42004-021-00478-2)
Supplement: Supplementary file 2 — Description of Additional Supplementary Files [file 42004_2021_478_MOESM2_ESM.pdf]

Description of Additional Supplementary Files

File Name: Supplementary Data 1
Description: Crystallographic information file for compound **3ha**.

File Name: Supplementary Data 2
Description: Crystallographic information file for compound **5aa**.
